# Inbred mouse strains reveal biomarkers that are pro-longevity, antilongevity or role switching

**DOI:** 10.1111/acel.12226

**Published:** 2014-05-23

**Authors:** Mark Moeller, Misa Hirose, Sarah Mueller, Catrin Roolf, Simone Baltrusch, Saleh Ibrahim, Christian Junghanss, Olaf Wolkenhauer, Robert Jaster, Rüdiger Köhling, Manfred Kunz, Markus Tiedge, Paul N Schofield, Georg Fuellen

**Affiliations:** 1 Institute for Biostatistics and Informatics in Medicine und Ageing Research, Rostock University Medical CenterRostock, Germany; 2 Department of Dermatology, University of LübeckLübeck, Germany; 3 Division of Gastroenterology, Department of Medicine II, Rostock University Medical CenterRostock, Germany; 4 Department of Hematology/Oncology/Palliative Medicine, Rostock University Medical CenterRostock, Germany; 5 Institute of Medical Biochemistry and Molecular Biology, Rostock University Medical CenterRostock, Germany; 6 Department of Systems Biology and Bioinformatics, University of RostockRostock, Germany; 7 Institute of Physiology, Rostock University Medical CenterRostock, Germany; 8 Department of Dermatology, Venereology and Allergology, University of LeipzigLeipzig, Germany; 9 Department of Physiology, Development and Neuroscience, University of CambridgeCambridge, UK

**Keywords:** aging, anti-aging, inflammation, lifespan, longevity, mice, senescence

## Abstract

Traditionally, biomarkers of aging are classified as either pro-longevity or antilongevity. Using longitudinal data sets from the large-scale inbred mouse strain study at the Jackson Laboratory Nathan Shock Center, we describe a protocol to identify two kinds of biomarkers: those with prognostic implication for lifespan and those with longitudinal evidence. Our protocol also identifies biomarkers for which, at first sight, there is conflicting evidence. Conflict resolution is possible by postulating a role switch. In these cases, high biomarker values are, for example, antilongevity in early life and pro-longevity in later life. Role-switching biomarkers correspond to features that must, for example, be minimized early, but maximized later, for optimal longevity. The clear-cut pro-longevity biomarkers we found reflect anti-inflammatory, anti-immunosenescent or anti-anaemic mechanisms, whereas clear-cut antilongevity biomarkers reflect inflammatory mechanisms. Many highly significant blood biomarkers relate to immune system features, indicating a shift from adaptive to innate processes, whereas most role-switching biomarkers relate to blood serum features and whole-body phenotypes. Our biomarker classification approach is applicable to any combination of longitudinal studies with life expectancy data, and it provides insights beyond a simplified scheme of biomarkers for long or short lifespan.

## Introduction

### Biomarkers of aging and animal studies

With the reduction in the burden of communicable disease and improvements in nutrition, attention is turning to longevity and the challenges that accompany increased longevity such as the desire for healthy aging (Lozano *et al*., 2012). The identification of biomarkers, which reveal information about the expected lifespan of an individual or predict the status of fitness or health in the near or distant future, is an important objective for aging research. As Baker & Sprott (1988) pointed out, chronological age is not necessarily a good predictor of functional or biological age, especially in later life. They defined a biomarker of aging as a measurable biological feature of an organism that *predicts* functional capacity at some later age better than chronological age. In many cases, the standard functional capacity to be predicted is life expectancy, measured in terms of lifespan. Biomarkers of aging then provide prognostic evidence, and they are derived from correlations between feature values at a certain age, or at a certain set of ages, and life expectancy. Finding prognostic biomarkers of aging thus requires the measurement of features in a (large) set of individuals exhibiting different lifespans, and such data are scarce. Even longitudinal animal data are few and far between and may not necessarily reflect the human situation (Zahn *et al*., 2007).

In animal experiments, some measurements are destructive (i.e. the animal must be sacrificed) or interventional (e.g. taking a sufficient amount of blood may markedly influence the physiology of an animal), so it is not always possible to determine the normal lifespan of the animal for which the measurements were taken. In that case, studies may be combined, with attendant problems of interpretation. If the animals used are genetically closely related groups such as inbred strains or breeds, then, for each strain, features can be measured at certain ages, and, independently, life expectancy can be estimated. For example, Urfer *et al*. (2011) enriched their breed-specific data on cataracts in dogs by mean life expectancy and body size derived from previous studies and reached the plausible conclusion that cataracts (as well as body size) are prognostic of lifespan. The Nathan Shock Center at the Jackson Laboratory has extensively characterized 32 commonly used inbred strains for aging-related phenotypes and made the primary data publicly available on the *Mouse Phenome Database* (Sundberg *et al*., 2011; Yuan *et al*., 2011; Maddatu *et al*., 2012). These data sets form the basis for the analyses presented here that permit investigation of prognostic biomarkers of aging by combining longitudinal studies of features (including blood count data, leucocyte data, blood chemistry, body composition, etc.) and a separate study of life expectancy, all done for about 30 strains. Standardization of environment, assays and strains means that this represents the most coherent and well-controlled lifelong study yet conducted in mammals. The same individuals were tracked allowing true longitudinal analysis, avoiding the interpretation problems of a cross-sectional study.

The strains of mice include a specific set of short-lived strains that are predisposed to specific diseases, triggering early death (Yuan *et al*., 2009). Our study includes these strains, but we also reanalysed our data excluding them. Moreover, we went further than (Yuan *et al*., 2009) by checking whether there are systematic differences between the longitudinal trends of the features, comparing short-lived to longer-lived mice. We find that there are few such differences. These observations imply that in general, the features we investigated change at a roughly similar speed in short-lived and longer-lived mice, and short-lived strains die early due to reasons not related to these features, whereas longer-lived strains age with slightly different speed towards the end of life. Our observations justify (*post hoc*) choosing the same chronological ages for all strains as time points for measurement and that measurements taken at a specific chronological age can be analysed by aggregating over all strains, no matter whether they are short-lived or longer-lived.

### Approaches to biomarker classification

As described, biomarkers of aging are often defined by their quality of being prognostic for life expectancy. As life expectancy data for many individuals or strains are scarce, however, many studies establish *biomarkers of age* instead of *biomarkers of aging*, and some even confuse both concepts, (Gavrilov & Gavrilova, 2006). The *biomarkers of age* concept is simply based on longitudinal or cross-sectional trends of features as a function of time. According to Gavrilov & Gavrilova, (2006), ‘the regular and progressive changes over time per se do not constitute aging unless they produce some deleterious outcome (failures)’. Using longitudinal evidence, biomarkers of age can therefore be *validated* as biomarkers of aging if something is known about their effects, using literature data to ascribe deleterious (negative) effects (or correlates thereof) to biomarkers whose values go up, and beneficial (positive) effects (or correlates thereof) to the ones whose values go down.

Importantly, prognostic evidence and validated longitudinal evidence for biomarkers of aging usually relate to an overlapping but not identical time span in the life of the animals. Any prognosis involves a later time point; therefore, prognostic biomarkers of aging tend to relate to *early* effects that have consequences upon the later life of the animals. In turn, longitudinal trends tend to involve *late* effects. Literature validation of the biomarkers we found based on longitudinal observations reveals that their upward or downward trend directly affects fitness at older ages. Our identification of role-switching biomarkers will mostly depend on the finding that a biomarker is classified as pro-aging (antilongevity) based on prognostic evidence, but as anti-aging (pro-longevity) based on validated longitudinal evidence. In these cases, the early effects that are relevant for prognosis are opposite to the late effects, where a longitudinal trend affects fitness. The situation is different in the clear-cut cases, where prognostic evidence and validated longitudinal evidence yield the same classification. Here, the direction of effects does not change as a function of time. We will discuss the role of early and late effects in more depth in the Discussion section.

In the analysis presented here, we search for biomarkers in the Nathan Shock Center study data set. For each feature, we first identified longitudinal trends by regression analysis. Such a regression is necessarily the same as the estimation of the correlation of the feature with the age of the animal. Longitudinal evidence without further validation yields *biomarkers of age*, so we checked the literature for deleterious or beneficial effects. Then, to check for prognostic evidence, we combined the data with the life expectancy data set. More specifically, validated longitudinal evidence and prognostic evidence allowed us to define a classification rule to distinguish three major classes of biomarkers: pro-longevity, pro-aging (antilongevity) and role-switching (antilongevity at early time points, pro-longevity at later time points). We do not necessarily suggest that the deleterious or beneficial effects we found in the literature provide mechanisms directly affecting longevity or aging; many of these should be considered correlative. Then again, as biological processes form deeply entangled networks, many of the effects may have both causative and correlative aspects.

## Results

### Clear-cut biomarkers of aging

**Figure 1:**
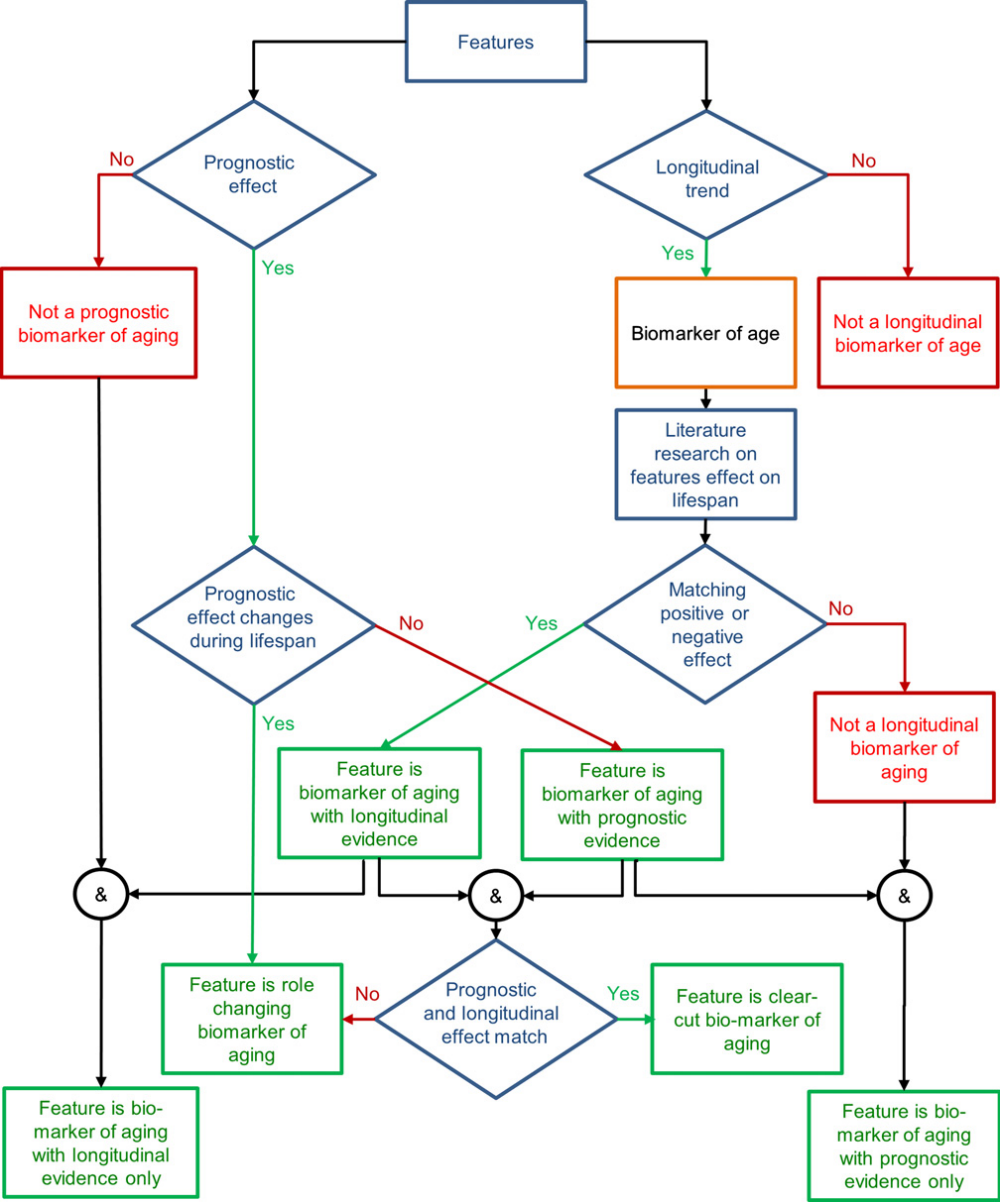
Protocol of our biomarker classification scheme.

We simultaneously performed regression to investigate the longitudinal trends of all data set features and correlation as well as Cox analyses to investigate their prognostic power for lifespan. From all data sets (Data S1 Table 1), we selected features as putative biomarkers if they had both validated longitudinal and prognostic evidence (see Fig. 1, Experimental procedures and Data S1 Table 3, for the rules employed). Table 1 summarizes the final classification of these biomarkers based on *both* their prognostic and longitudinal evidence. We note seven clear-cut cases where both kinds of evidence are corroborative. These are related to immune cells (B cells, lymphocytes; neutrophils), anaemia (red blood cell count and linked measurements) and inflammation (magnesium, neutrophils), and their classification as pro-longevity or antilongevity (neutrophils only) is clear cut. In these cases, there are potential mechanisms already reported that can be invoked to explain the observation. Specifically, the decrease in lymphocytes/B cells as protagonists of adaptive immunity and the increase in neutrophils suggest an age-related shift of the immune system cell composition from adaptive to innate, driven by cellular effects of proinflammatory cytokines and chemokines. The anti-inflammatory effects of magnesium are described in Barbagallo *et al*., (2009).

**Table 1:** Feature analysis results based on our biomarker classification protocol. Corroborative evidence exists if the prognostic and the longitudinal approaches to biomarker classification agree. Conflicting evidence exists if the prognostic data (usually concerning early effects with consequences later in life) trigger a different classification as compared to the validated longitudinal data (usually concerning late effects). Such conflicting evidence can be resolved by suggesting that the early effects of high biomarker values are opposite to the late effects. In case of thyroxine, this role switch is already found in the prognostic data alone. Qualifiers are indicating that observations are more pronounced for a specific gender (e.g. ‘pronounced in male’) or found (mostly) in specific age groups (6, 12, 18 or 24 M; M: months)

Feature	Abbrevation	Evidence		Known effect		Classification	
		Prognostic	Longitudinal	Early	Late	Early	Late
Corroborative evidence, clear-cut cases							
B cells, lymphocytes	B cells, LYMPH	Long lifespan (pronounced in female)	Down	Anti-immunosenescent		Pro-longevity	
Red blood cells, haemoglobin, hematocrit	RBC, HGB, HCT	Long lifespan (pronounced in male)	Down	Anti-anaemic		Pro-longevity	
Magnesium	Mg	Long lifespan (pronounced at 12 M)	Down (pronounced in female)	Anti-inflammatory		Pro-longevity	
Neutrophils	NEUT	Short lifespan	Up	Pro-inflammatory		Antilongevity	
Conflicting evidence, resolvable by a role switch							
Iron, also reticulocyte corpuscular haemoglobin	Fe, CHr	Short lifespan (pronounced at 12 M)	Down	Damaging, in particular by oxidative stress	Anti-anaemic	Antilongevity	Pro-longevity
Thyroxine	T4	Short lifespan (6 M, pronounced in male), long lifespan (12 M/18 M, pronounced in female)	Down	Pro-anabolic	Pro-robustness	Antilongevity	Pro-longevity
Body mass index, heart rate	BMI, HR	Short lifespan (pronounced at 6 M)	Down	Pro-anabolic	Pro-robustness	Antilongevity	Pro-longevity

### Role-switching biomarkers of aging

There are another five cases recorded in Table 1 where the two kinds of evidence are in conflict and a role switch is suggested. These are related to oxidative damage and anaemia (total serum iron and the CHr measurement that is closely linked (Mast, 2002), with a weaker pattern) or to metabolism and robustness (thyroxine, BMI, as well as heart rate with a signal at 6 M; M = months). The apparently paradoxical classification of the role-changing biomarkers as ‘early antilongevity, late pro-longevity’ is biologically plausible and may be resolved. For example, a switching role of serum iron may be explained from its known role as a protagonist of oxidative damage through catalytic generation of ROS (dominating its early effect) (Xu *et al*., 2010) and as a correlate of anaemia (dominating its late effect). The switching role of thyroxine (T4) follows already from its prognostic evidence alone. It is the only feature with a switch in prognosis, predicting short lifespan at 6 M and long lifespan at 12 M/18 M. Both cases may be explained by its well-established promotion of anabolism and growth. Accordingly, we suggest that its early ‘cellular hyperactivity’ confers metabolic and physiological decompensation in later life, such as obesity and insulin resistance. In contrast, its later activity contributes to a reasonable amount of overweight that yields a state of robustness. This pattern is reflected by body mass index and heart rate, with essentially the same rationale. In particular, body mass index (BMI) is discussed extensively in the literature. For a long time, a high body mass index defining overweight status was considered disadvantageous for young and old people alike. More recently, however, it was found that for older people, overweight (but not obesity) has advantageous effects, possibly due to the higher robustness it affords against disease (Auyeung & Lee, 2010; Flegal *et al*., 2013).

### Detailed analysis and visualization of blood-based biomarkers

**Figure 2:**
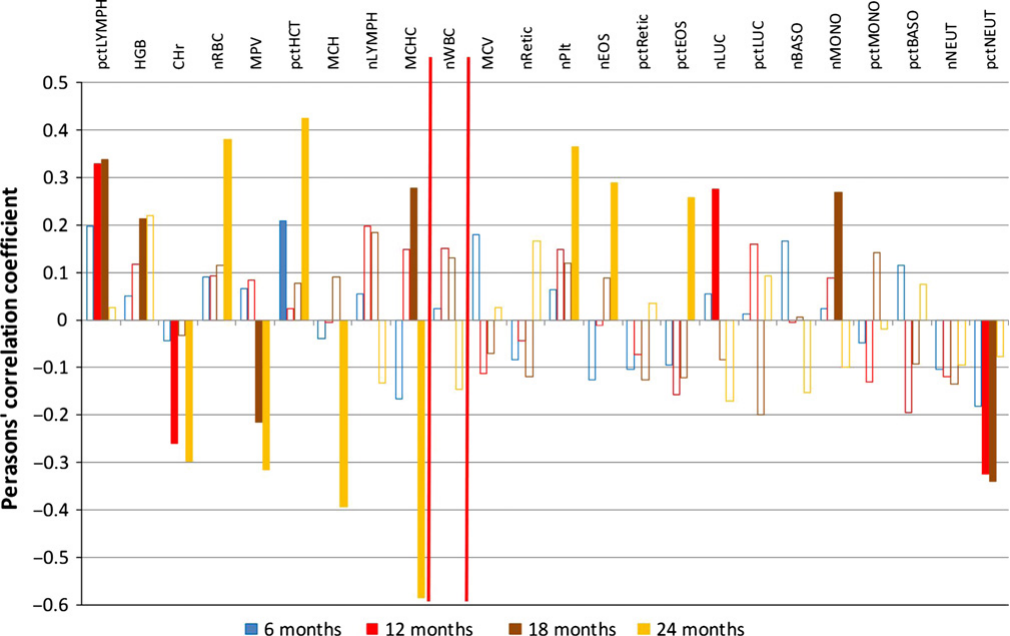
Correlation analysis – female complete blood count data (data set: Peters4); irrelevant values (correlation < |0.2| or/and *P*-value < 0.05) are presented by open bars. Two red delimiters allow distinguishing, from left to right, features with a significant downtrend, features with no longitudinal trend and features with a significant uptrend. nBASO, basophil count; pctBASO, basophil percentage; CHr, reticulocyte corpuscular haemoglobin; nEOS, eosinophil count; pctEOS, eosinophil percentage; HGB, haemoglobin; nLUC, large unstained cells count; pctLUC, large unstained cell percentage; nLYMPH, lymphocyte count; pctLYMPH, lymphocyte percentage; MCH, mean RBC corpuscular haemoglobin content; MCHC, mean RBC haemoglobin concentration; MCV, mean RBC corpuscular volume; nMONO, monocyte count; pctMONO, monocyte percentage; MPV, mean platelet volume; nNEUT, neutrophil count; pctNEUT, neutrophil percentage; pctHCT, hematocrit; nPlt, platelet count; nRBC, red blood cell count; nRetic, reticulocyte count; pctRetic, reticulocyte percentage; nWBC, white blood cell count.

**Figure 3:**
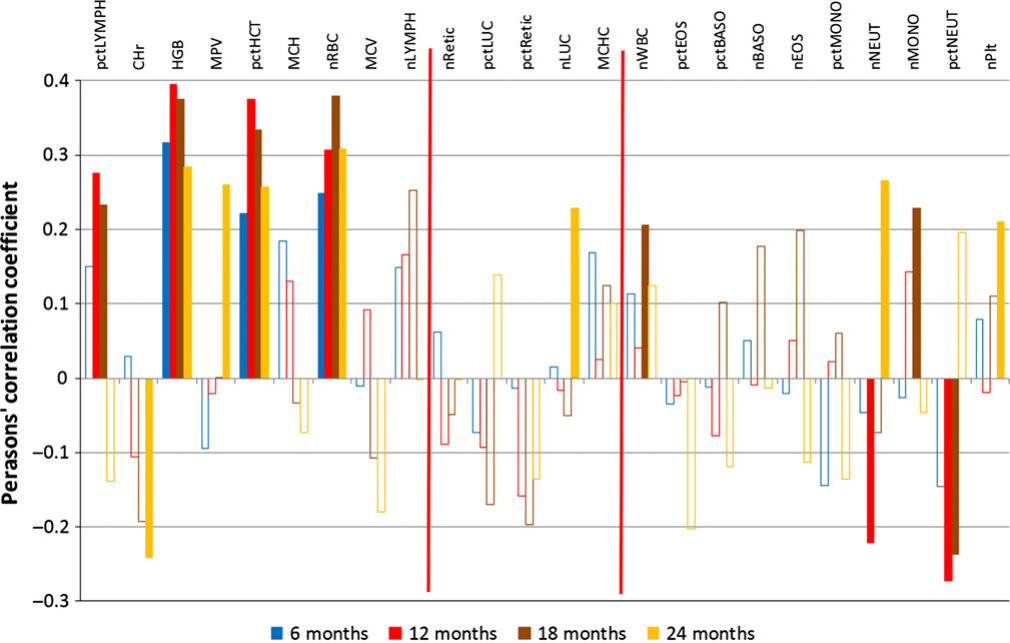
Correlation analysis – male complete blood count data (data set: Peters4). See Fig. 2 for further explanations.

**Figure 4:**
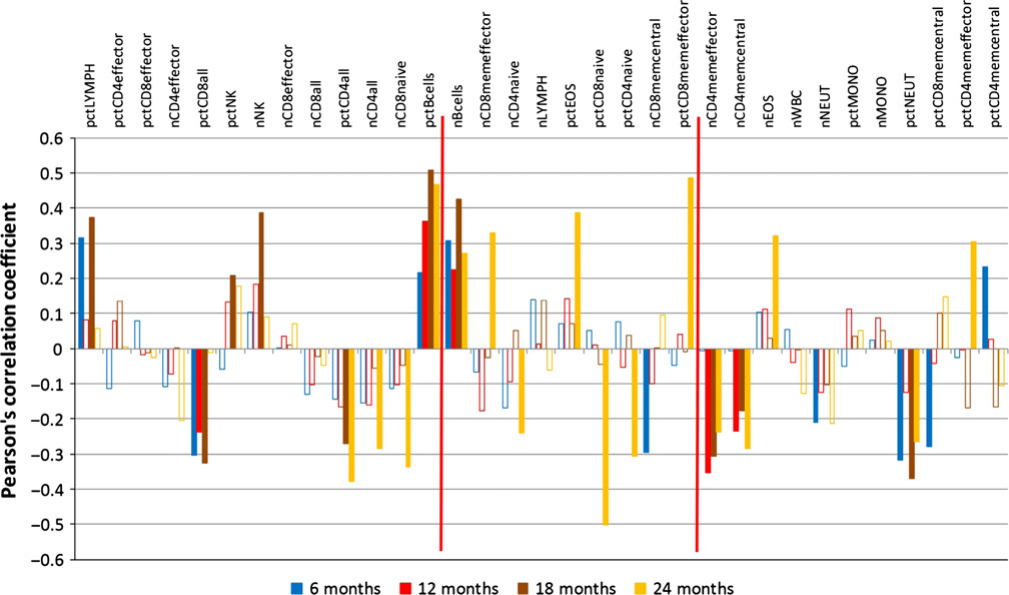
Correlation analysis – female leucocyte data (Data set: Petkova1); see Fig. 2 for details. The following abbreviations are used; for each abbreviation starting with ‘pct’ referring to a percentage, an analogous abbreviation with the prefix ‘n’ refers to the absolute count. pctBcells, B cells; pctCD4all, CD4 T-cells percentage; pctCD4effector, CD4 T-cell subtype – effector cells percentage; pctCD4memcentral, CD4 T-cell subtype – central memory cells percentage; pctCD4memeffector, CD4 T-cell subtype – effector memory cells percentage; pctCD4naive, CD4 T-cell subtype – naive cells percentage; pctCD8all, CD8 T-cells percentage; pctCD8effector, CD8 T-cell subtype – effector cells percentage; pctCD8memcentral, CD8 T-cell subtype – central memory cells percentage; pctCD8memeffector, CD8 T-cell subtype – effector memory cells percentage; pctCD8naive, CD8 T-cell subtype – naive cells percentage; pctEOS, eosinophil percentage; pctLYMPH, lymphocyte percentage; pctMONO, monocyte percentage; pctNEUT, neutrophil percentage; pctNK, NK cells percentage; nWBC, white blood cell count.

**Figure 5:**
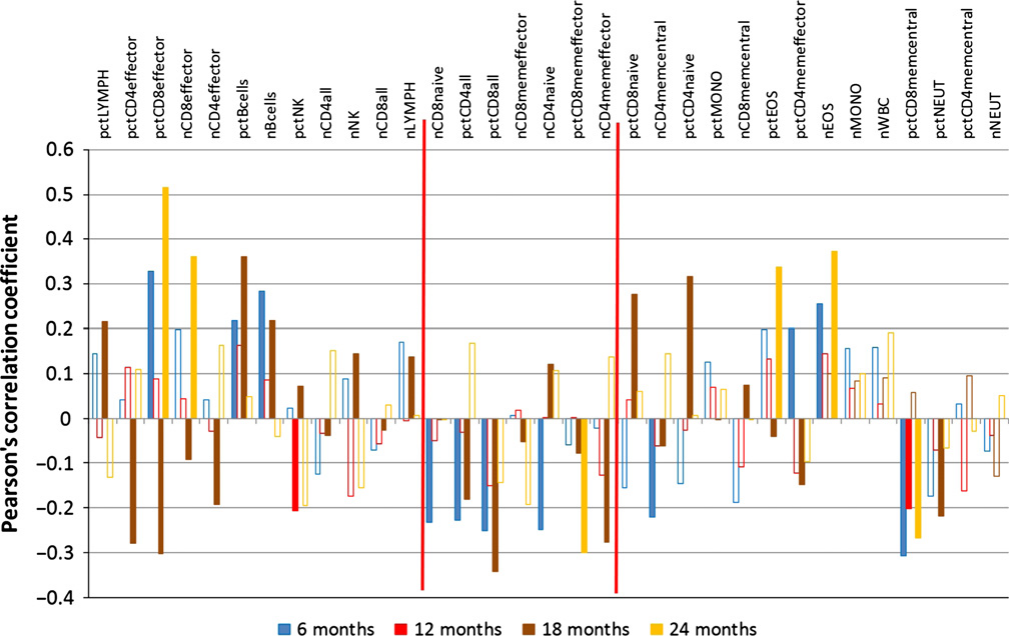
Correlation analysis – male leucocyte data (Data set: Petkova1). See Fig. 4 for further explanations.

**Figure 6:**
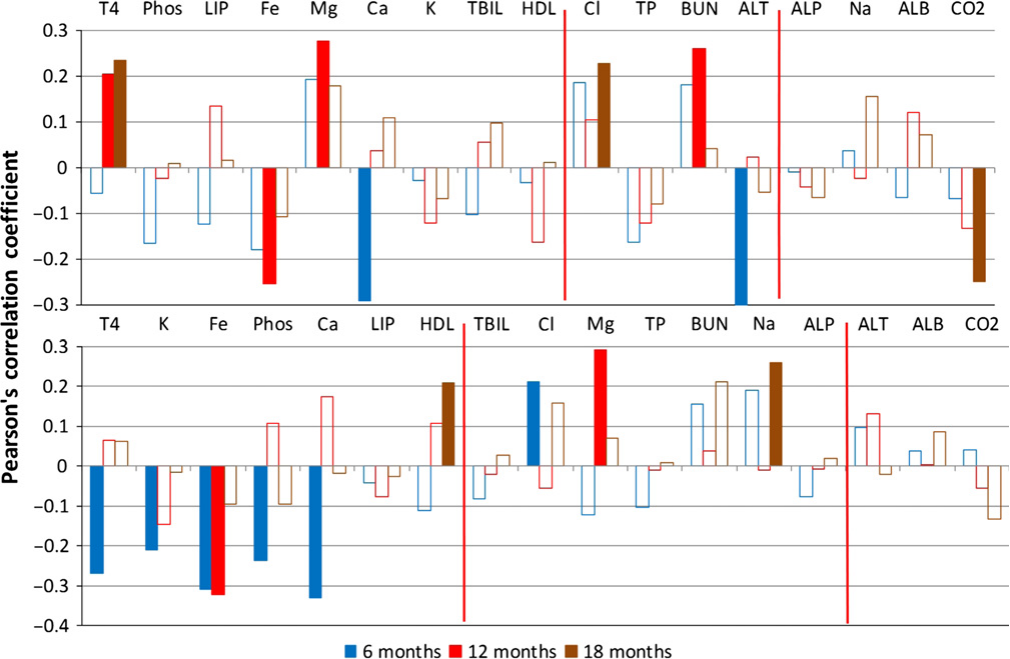
Correlation analysis – blood chemistry (data set: Yuan3); top: female, bottom: male; see Fig. 2 for details. ALB, plasma albumin; ALP, serum alkaline phosphatase; ALT, plasma alanine aminotransferase; BUN, plasma blood urea nitrogen; Ca, serum calcium; Cl, serum chloride; CO_2_, dissolved-ionized carbon dioxide; Fe, serum iron; HDL, plasma HDL cholesterol; K, serum potassium; LIP, plasma lipase; Mg, serum magnesium; Na, serum sodium; Phos, serum phosphorus; T4, serum thyroxine; TBIL, serum bilirubin; TP, plasma total protein.

Prognostic evidence in the blood count, leucocyte and blood serum chemistry data is described by correlation charts in Figs 2–6. Moreover, the order of features in these charts, from left to right, gives an indication of their longitudinal trends, from negative (going down) to positive (going up). The precise regression slopes for the longitudinal evidence are given in Figs S7, S8 and S13 of Data S1 (Supporting information). Charts for all other data sets are found in Data S1 (Supporting information) as well. For all data sets, selection and classification of features can proceed in their regression-based order, from left to right, checking the prognostic power of the leftmost and the rightmost features (up to the red delimiter) with a significant downtrend or uptrend, respectively. There are few gender-specific differences in the regression-based ordering of the features. For example, in the blood count data (Data S1 Fig. S7), there is only a notable difference for the platelet count (nPlt) that is not prognostic.

The *blood count* data of female mice (Fig. 2), and, more pronounced, of male mice (Fig. 3) display the clear-cut pattern we already reported for lymphocytes, red blood cells (and, related, haemoglobin and hematocrit) as well as for neutrophils (Table 1). Thus, features such as the red blood cell count on the left, whose values go down longitudinally, tend to be features with positive correlations to life expectancy, and vice versa (for the neutrophil count on the right). In other words, the clear-cut biomarkers with both validated longitudinal and prognostic evidence are clearly exposed. For the red blood count, prognostic evidence is much weaker for female than for male mice, possibly due to the stronger role of other factors (e.g. hormonal changes) in female aging processes. On the other hand, for the lymphocyte data (T cells and B cells, see the next paragraph), prognostic evidence is weaker in male compared with female mice, possibly due to ‘age-associated B cells’ (ABCs) that were recently found in female mice only and implicated in autoimmunity (Rubtsov *et al*., 2011; Kogut *et al*., 2012). ABCs are supposed to originate from mature naïve B-cell precursors, so we may hypothesize that ABCs and ‘normal’ B cells are, to a certain extent, mutually exclusive. Then, in female mice, ABCs are indicators of aging at the expense of ‘normal’ B cells, and the latter are prognostic for long lifespan. The absolute number of lymphocytes (nLYMPH) decreases slightly during aging and their percentage (pct-LYMPH) strongly, while at the same time, the numbers of cells of the innate immune system increase (i.e. monocytes, basophilic, eosinophilic and, in particular, neutrophilic granulocytes). Thus, as a consequence of age, the immune system composition shifts from the adaptive towards the innate. This reflects the processes of ‘inflammaging’ (Franceschi *et al*., 2006) and of ‘immunosenescence’ by involution of lymphocyte-producing thymic tissue, that is, the degeneration of the organ where these cells differentiate/mature (Domínguez-Gerpe & Rey-Méndez, 2003; Takahama, 2006; Chinn *et al*., 2012). Consistent with this argument, a ‘T-cell activation’ module has been recently identified in humans, ‘marking age-associated shifts in lymphocyte blood cell counts’ (Van den Akker *et al*., 2014). The increase of neutrophil numbers was also reported by others (Kovacs *et al*., 2009), as was an age-dependent increase in granulocyte/macrophage progenitor cells (GMPs) (Rossi *et al*., 2005). In terms of percentages, a compositional shift in our blood count analyses may be triggered if only one fraction changes in absolute numbers. However, both fractions (lymphocytes and neutrophils) change in absolute numbers, in the same direction as the percentages. The same measurements can also be found in the leucocyte data set, but with a stronger focus on neutrophil decline.

The *leucocyte* data set (Figs 4 and 5) closely matches the blood count data set, for the features included in both. Additionally, a pronounced decline is found in the leucocyte data set for many cells of the adaptive immune system (CD4 and CD8 T cells, common suffix ‘all’) and, in particular, for various effector cells (common suffix ‘effector’) and natural killer (NK) cells as well as B cells. Some other leucocyte percentages increase over time, such as memory cells (common suffix ‘mem’) and monocytes. The longitudinal increase but low prognostic value of most memory cells may have a straightforward cause: these cells simply accumulate as the immune system learns; they are just biomarkers of age. The CD4 memory (effector) cells (nCD4memeffector) are an exception, already noted by Miller *et al*. (1997). These cells are *prognostic* for a short lifespan in female mice (12, 18 and 24 M data), similarly to CD8 memory central cells (pctCD8memcentral) in male mice, and while their number increases longitudinally as expected for antilongevity biomarkers, we could not find a deleterious effect reported in the literature for either. In fact, Miller *et al*., (1997) extensively discussed three explanations of their prognostic value without reaching a conclusion. Moreover, these authors point out a shift from naïve/virgin T cells to memory cells. The loss of the former reflects immunosenescence. An age-related increase in CD4 memory effector cells has also been previously described (Saule *et al*., 2006). Finally, the percentage of all CD8 cells (pctCD8all) is declining longitudinally, but it is prognostic for a short lifespan, specifically in female mice (6, 12 and 18 M). As such, it is a candidate for a role-switching biomarker (‘antilongevity early, pro-longevity late’), but we could not find literature evidence for a beneficial effect on lifespan that may be reduced as it declines.

In *blood serum chemistry* (Fig. 6), apart from magnesium, thyroxine (T4) and iron as described above, we found some more interesting features, which had, however, at most isolated prognostic value, possibly because there is a strong influence of confounding factors, some of them related to intake and excretion. Findings for these blood chemistry features are described in Appendix S1 Results.

### Re-analysis of data using Cox regression

To validate our results by a different method, Cox regression was used as an alternative to lifespan correlation. Importantly, Cox regression analysis confirmed our results presented in Table 1 (Data S4); concordance of both types of analysis was already noted by Harper *et al*. (2004). In some cases, we even observed a stronger signal, with higher consistency between age groups and sexes: the percentages of lymphocytes and neutrophils as well as CHr are more concordant in 6-month-old female mice. This is also true for the red blood cell count (RBC) in females, which is also more concordant with male, and in both sexes, the platelet count (Plt) is more concordant with results obtained from longer-lived strains. In males, the CHr signal is also stronger, but the signal for lymphocytes and neutrophils is comparatively weaker. Natural killer cells and HDL (high-density lipoprotein) emerge as markers in females, while K (potassium) emerges as a marker in males. We also performed a multivariate Cox analysis of the biomarkers listed in Table 1. As shown in Data S5 (Supporting information), in 6-month-old mice, for example, the most significant features are the number of B cells in females, and the number of lymphocytes and the level of thyroxine (T4) in males.

### Re-analysis of data based on longer-lived strains only

The results of our re-analysis, excluding the short-lived strains, are documented in Data S2 (Supporting information). There are few relevant features gained or lost in the longer-lived strains, as follows. For the blood count data, the platelet count (Plt) surfaces as a strong pro-longevity prognostic marker, with a longitudinally increasing trend, particularly in males. The trend is counter to that seen in humans where there is a general trend downwards with age (Lai *et al*., 2010). Several strains have notably high platelet numbers at 18 and 24 M; these are WSB/EiJ, C57BL/6J and C57BL/10J. Such increased numbers may be of advantage in cases of bleeding, possibly associated with aggressive behaviour, or protective against cardiovascular events such as haemorrhagic stroke. In females, the number of eosinophils (nEOS) goes up longitudinally (Data S2 Fig. 6), associated with a long lifespan (Data S2 Fig. 7). This effect has so far not been described in the literature. A result that receives weaker support is the prognostic value of T4 in females at a later age. Furthermore, the support for B cells as pro-longevity markers is no longer as significant as before, particularly in females. However, many results receive stronger support if the short-lived strains are excluded. Among female mice, the affected features are CHr, pctHCT, nRBC, Fe and Mg, and among male mice, our observations regarding lymphocytes and neutrophils are now more strongly supported. In the body composition data, the BMI (as well as percentage fat) becomes prognostic for longevity of 20 M male mice, corroborating its contribution to robustness already discussed. Finally, the exclusion of the short-lived strains markedly increased the negative correlation between IGF and life expectancy, confirming the observation by Yuan *et al*. (2009).

### Comparison of longitudinal trends of short-lived and longer-lived mice

We finally investigated whether short-lived mice display the same longitudinal trends as longer-lived mice, and the results of our trend comparison are found in Data S3 (Supporting information). We note few systematic patterns as follows, in the body composition, leucocyte and blood chemistry data. AKR/J mice show faster downtrends in terms of body mass index (and some related measurements with negligible longitudinal trends), possibly due to the thymic lymphoma they develop by 18 M of age (Storer, 1966; Brayton *et al*., 2012). Regarding the leucocyte data, males of the SJL/J strain are singled out by enrichment in opposite trends. This strain is known to show aggressive behaviour in males, leading to injury and infection and truncation of lifespan. In case of blood chemistry, Calcium shows a faster trend in many strains, while HDL and TP (total protein) show an opposite trend, possibly due to liver malfunction.

## Discussion

The most specific feature of any data analyses in aging research is a focus on longitudinal data. We present here a detailed analysis of the most extensive longitudinal mouse phenotype data set published to date. We have integrated our findings with an extensive review of the literature; this was an essential component in our analysis pipeline and we present the analysis results with extensive links to observations already published, including pointers to observations in human. In the discussion that follows, we point out some more general aspects of our work. First, we relate our results to an interpretation of Miller *et al*. (1997), comparing feature values in young and old animals. Next, we relate our observations to work by Harper *et al*. (2004), on combining biomarkers. Finally, we discuss the perhaps most far-reaching aspect of our analysis. Specifically, we suggest that we could demonstrate that a critical assumption of many previous longitudinal analyses, that is, the validity of selecting the same measurement time points for all individuals (strains) of a study, irrespective of differences in individual longevity due to specific early-onset diseases, may indeed be justified, by showing that (i) data from the short-lived strains did not affect the major conclusions of our study and (ii) the trends of the features in the short-lived strains generally agree with the ones in the longer-lived strains.

### Biomarker values in young animals compared with values in older animals

We have described a biomarker analysis protocol that is applicable whenever there is access to longitudinal features, corresponding life expectancy data and literature knowledge. Applied to the *Nathan Shock Center* data set of the *Mouse Phenome Database*, our protocol enabled us to detect meaningful pro-longevity, antilongevity and role-changing biomarkers, the former with prognostic and longitudinal lines of evidence. We have already discussed in the Introduction that these two lines of evidence for biomarkers of aging usually relate to an overlapping but not identical time span in the life of the animals. In case of clear-cut longitudinally declining pro-longevity biomarkers, their early prognostic role (high values predict a long lifespan) could in fact be based on large amounts of effectors (cells) with a positive effect throughout life (despite the decline), and their late longitudinal role could be based on their gradual loss at old age. This interpretation reflects the hypothesis of Miller *et al*. (1997) that young mice, whose feature values resemble those of old mice, tend to have a short life expectancy and vice versa. High biomarker values at young age may thus enable mice to ‘resist’ any loss and are prognostic of longevity; if a younger mouse features biomarker values indicating that it can compensate the normal age-based trend of a feature when it becomes older, this mouse has a good chance of living longer. Examples for this hypothesis in the blood count data are the lymphocyte, B-cell and red blood cell counts, as well as the neutrophil counts. The latter reflect ‘inflamm-aging’ as described. We note that such chronic inflammatory processes are common to aging mice (Ray *et al*., 2010; Pettan-Brewer & Treuting, 2011), consistent with similar findings in aging humans (Krabbe *et al*., 2004). Elevation of neutrophil numbers in younger mice may be due to several specific causes, see the Appendix S1 Discussion.

### Biomarker combination

Biomarkers may be combined across data sets for optimal prediction of lifespan. Corroborating our analysis, Harper *et al*., (2004) used (i) T-cell features (CD4 and CD8 cells, CD4 and CD8 memory cells as well as CD4 and CD8 cells featuring P-glycoprotein, from 8- and 18-month-old mice), (ii) body weight (from 3-month-old mice) and (iii) thyroxine and leptin levels (from 4-month-old mice) to construct a combined classifier with better prognostic value than classifiers based only on a subset of these features. The most prognostic T-cell feature in young mice turned out to be the percentage of CD8 memory cells in the pool of all CD8 cells. Inclusion of thyroxine was based on its prognostic value in young male mice. The biomarkers they used are thus closely related to the biomarkers we documented. In particular, in the data based on male mice we studied, CD8 memory central cells were found to be prognostic of short lifespan and going up longitudinally, but without a known deleterious effect. Insofar as the biomarkers are based on young mice only (in case of body mass and thyroxine), we documented them as role-switching markers, with an early antilongevity effect.

### Biomarkers in short-lived vs. longer-lived animals

We included data from short-lived strains in the analyses presented. It is possible that the specific diseases affecting these strains bias the results of analyses aiming to find biomarkers of aging. However, our re-analysis based on all longer-lived strains, excluding the short-lived ones, yielded essentially the same results (Data S2); a similar procedure carried out for the same reasons by Yuan *et al*. (2009) was shown to result in only small changes in the correlation of IGF1 levels with longevity. Moreover, we observed that the features we investigated display very similar longitudinal trends in short-lived and longer-lived mice (Data S3). This result provides a justification for comparing measurements according to age groups in the first place. In the extreme case, if the features in short-lived mice were changing ‘twice as fast’ as in longer-lived mice due to their specific diseases, 6 M measurements of the former would need to be compared to 12 M measurements of longer-lived mice, and 12 M measurements of the former to 24 M measurements of the latter. More generally, measurement timelines would need to be aligned, and this task is difficult or impossible in hindsight. However, in our comparison of trends, such systematic patterns are rare, so features change at a roughly similar speed in short-lived and longer-lived mice. Thus, we suggest that along their life, mice age at a roughly similar speed, and early death is usually due to genetic predisposition to *specific diseases*. In turn, late death is influenced significantly by *other effects (that relate closely to aging) and an increasing burden of chronic disease and co-morbidities*. That is, only towards the end of life, the speed of aging becomes markedly slower and more diverse in the longer-lived strains.

We have confirmed and extended current knowledge in our detection of clear-cut biomarkers. Our insights into role-switching biomarkers consolidate previous knowledge, related to inflammation and immunity on one hand and to anaemia and body mass on the other. Interestingly, connecting both aspects is a focus of recent research. For example, diet-induced obesity is known to affect the adaptive immune system negatively (Dixit, 2012). Also, the role of trade-offs was recently discussed in terms of robustness, citing inflammation and body size as examples (Kriete, 2013). We believe that role-switching is an important point to consider in biomarker analysis. Such considerations may also have practical consequences, as biomarkers may point to possible intervention points. Accordingly, interventions designed to promote longevity may be useful, useless or harmful depending on the age at intervention. While the data set we analysed is the most comprehensive one publicly available at this time, analyses and conclusions are of course limited by the coverage it provides, with a focus on blood. However, with decreasing costs for high-throughput analysis of the genome, biofluids, gut microbiome, etc., our strategy can be easily extended to such data sets.

## Experimental procedures

### Data

In our analysis, we used seven longitudinal studies of the *Nathan Shock Center data set*, available as of July 2013 from the Jackson Laboratory *Mouse Phenome Database* (Maddatu *et al*., 2012) (Data S1 Table S1). For details, we referred to the webpage of the *Mouse Phenome Database* (http://phenome.jax.org). All studies used between 29 and 33 inbred mouse strains raised and taken care of under similar conditions. Data included life expectancy, as well as blood features, bone mineral density and body composition, DNA damage, urine values and electrocardiogram data.

### Simple linear regression to detect longitudinal evidence

To identify the longitudinal trend of a feature over the lifespan of individuals, we performed simple linear regression modelling using the measured values as the dependent variable and the age as the explanatory variable. To be able to compare the slopes between different features, we standardized the data by subtraction of the mean and division by the standard deviation. Regression was performed using the method *lm* for linear models, available as part of an R package. For each data set, the measured slopes *m* can be converted into Pearson’s correlation coefficients *r* by the formula 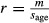, where *s*_age_ denotes the standard deviation, taken over all ages, of the distribution of the normalized values for the measurements. The regression analyses we performed are therefore equivalent to correlation analyses between the features and the age the measurement was taken. We considered only slopes as significant for which *P*-values (resulting from a two sided *t*-test) were smaller than 0.05 (i.e. α = 0.05). We used regression charts to visualize the results of the regression analyses, sorting features by slope. Originally, for the blood count data, only the absolute number of white blood cells and the percentages of the other cell types were given in the *Mouse Phenome Database*. For completeness, we calculated the absolute cell numbers from percentages for all cell types before performing the regression. Sample regression analyses are visualized for the features RBC (red blood cell count) and NEUT (neutrophil count) in Fig. S12 of Data S1 (Supporting information).

### Correlation of features and life expectancy, and Cox regression to detect prognostic evidence

No longitudinal data set included the life expectancy of the individual mice, so we added the respective life expectancy from data set Yuan2 to each data record in all other data sets. Again, we standardized the data by subtraction of the mean and division by the standard deviation. Then, we calculated Pearson’s correlation coefficient as implemented in the method *cor.test*, available within the r package *stats*, between feature value and life expectancy. This was done separately for each time point available (i.e. age groups 6, 12, 18, 20 and 24 M). Notably, a positive correlation means that high feature values are prognostic for a long lifespan, that is, longevity and vice versa. We only consider features as *relevant* in terms of their prognostic evidence, if Pearson’s correlation coefficient |*r*| ≥ 0.2 and *P*-value < 0.05 holds. In a correlation chart, the correlation coefficients are plotted as histograms. Open bars represent irrelevant values. The horizontal ordering of the features in each correlation chart was taken directly from the regression analysis of the same-sex mice of the same data set. Two vertical red delimiters delineate, from left to right, features with a significant downtrend, features with no clear trend and features with a significant uptrend. This enables a visual impression of how the longitudinal evidence relates to the prognostic evidence of the same feature and eases the selection of putative biomarkers as described in the next paragraph. Univariate and multivariate Cox regression were then done using the r package ‘survival’. In Data S4 (Supporting information), for each measured value, we present its univariate Cox regression coefficient, which would give rise to a hazard ratio by exponentiation. A negative Cox coefficient results in a hazard ratio smaller than 1 (i.e. a risk decrease) and a positive value in a risk increase. Thus, we inverted the *y*-axis to allow direct comparison with the corresponding correlation chart data. Multivariate Cox regression was applied to the biomarkers of Table 1, exploiting data from 10 to 27 strains (depending on sex and time point) for which no values were missing, see Data S5 (Supporting information).

### Biomarker selection and classification

We performed a detailed analysis of all relevant features that may qualify as biomarkers of aging based on the following two criteria: (i) the regression analysis yielded a significant slope, that is, the feature displays a longitudinal trend; (ii) the correlation analysis also demonstrated that the feature has prognostic evidence, at least for some age groups. For each feature we selected, we then conducted a literature search for what is known about its effects. Longitudinal evidence indicates a biomarker of aging (not just of age) if there is validation by a matching effect (i.e. a beneficial effect for features with a downtrend and vice versa) (Gavrilov & Gavrilova, 2006). Based on regression, correlation and literature data, biomarkers were then classified by the rules described in Fig. 1 and Table S3 in Data S1 (Supporting information), and the results were recorded in Table 1. The entire classification protocol, including the final designation as a clear-cut or role-changing biomarker, is given in Fig. 1. Specifically, biomarkers prognostic of a long lifespan are called pro-longevity, and biomarkers prognostic of a short lifespan are called antilongevity. Biomarkers with a validated longitudinal uptrend are also called antilongevity, and biomarkers with a validated longitudinal downtrend are called pro-longevity. Biomarkers attract a clear-cut classification where there is corroborative evidence. In particular, features whose values *go down longitudinally, with a matching beneficial effect, and predict long lifespan* or vice versa are thus clearly classified as pro-longevity or antilongevity, respectively. In case of conflicting evidence, we classified the features as role-switching. The classification scheme in Fig. 1 is formulated for an ideal case. In reality, the patterns of feature regression and correlation are more or less pronounced, due to biological variability. We therefore added some qualifiers regarding the resulting classification of biomarkers (Table 1). Moreover, isolated or inconsistent prognostic evidence of the 18, 20 and 24 M age groups was ignored, because data for these age groups are clearly the most volatile. In particular, due to the different life expectancies of the mouse strains (Data S1 Table S1), only one-third of all strains contributed data to the 24 M age group. Re-analysis of data based on longer-lived strains only and comparison of longitudinal trends of short-lived and longer-lived mice are described in Appendix S1 Experimental Procedures.

## Supporting Information

**Appendix S1** Supplementary experimental procedures, results and discussion, with references.

**Data S1** Supplementary tables and figures: linear regression and correlation charts.

**Data S2** Linear regression and correlations charts excluding short-lived strains.

**Data S3** Comparison of longitudinal trends.

**Data S4** Supplementary figures: univariate Cox regression.

**Data S5** Supplementary tables and figures: multivariate Cox regression.
