# Supplementary material for: Inbred mouse strains reveal biomarkers that are pro-longevity, antilongevity or role switching
Source: Aging Cell. 2014 May 23;13(4):729–38. doi: 10.1111/acel.12226 (PMC4326954; doi:10.1111/acel.12226)
Supplement: Supplementary file 1 — Appendix S1 Supplementary experimental procedures, results and discussion, with references. [file acel0013-0729-sd1.pdf]

Supplementary Experimental Procedures, Results and Discussion

Supplementary Experimental Procedures.

Re-analysis of data based on longer-lived strains only. We re-analyzed the data by the procedures described in the main text, but excluding, in a sex-specific way, all strains with expected lifespans of less than 600 days. This manipulation was found in the study by (Yuan et al. 2009) to improve the correlation between IGF-I levels and longevity, and is designed to answer the question whether these short lived strains bias the overall analysis because they suffer from high penetrance genetic susceptibility to defined lethal disease early in life. In particular, short-lived strains are female CAST/EiJ, MRL/MpJ, NZO/HILtJ, PL/J and SJL/J as well as male BTBR T+tf/J, BUB/BnJ, CAST/EiJ, FVB/NJ, NZO/HILtJ, PL/J and SJL/J. Results are presented in Supplement II.

Comparison of longitudinal trends of short-lived and longer-lived mice. We also checked for systematic bias that may confound the analysis as presented, checking whether the features of the short-lived strains behave significantly different from features of the longer-lived ones, as follows. We divided the strains into two groups according to their life expectancy (short-lived, < 600 days; longer-lived, > 600 days). For each combination of strain, sex and measurement we performed the simple linear regression as described before, but separately for the short- and the longer-lived strains, and compared the slopes using a two sided t–test (α=0.05). To cope with the different sample sizes, we used the Welch–Satterthwaite equation to calculate an approximation of each effective degree of freedom (that is used by the t-test) and applied the Benjamini-Hochberg procedure to adjust for multiple testing. The significant observations were then classified as (a) feature measurements among the short-lived strains that are decreasing or increasing stronger compared to the longer-lived strains (“faster trend”), (b) feature measurements among the short-lived strains that are decreasing or increasing weaker compared to the longer-lived strains (“slower trend”) and (c) feature measurements among the short-lived strains that are decreasing if the measurements among the longer-lived strains are increasing or vice versa (“opposite trend”). Results are presented in Supplement III.

Supplementary Results.

Blood serum data include some interesting features not covered in the main text. Calcium levels at six months in both males and females show an interesting predictive pattern with high levels at 6 months predicting short lifespan. Severe hypercalcemia is a potentially lethal condition, but chronic or possible spiking acute episodes of hypercalcemia have been implicated in degenerative human disease. Elevation of serum calcium levels is associated with a predisposition to cardiovascular disease (Bolland et al. 2010; Bristow et al. 2013). A possible link between high calcium levels in serum and longevity might be through the enhanced disposition to cardiovascular calcinosis, and in particular dystrophic cardiac calcinosis (DCC) is known to be a significant pathology in some strains. However the pattern we see is not sufficiently consistent to implicate this mechanism. C3H/He and 129/S1 mice both show high serum calcium levels and are predisposed to dystrophic cardiac calcinosis. They possess the TT splice variant in the ABCC6 genes (dyscalcin1 allele) (Aherrahrou et al. 2008). However MRL/MpJ mice show the highest serum calcium levels at 6 months of all strains investigated, but do not show DCC. KK mice show a high incidence of DCC (Berndt et al. 2013) yet show average (female) or low (male) levels of serum calcium. A search of the literature does not implicate other high calcium strains in high levels of calcinosis. Lipase activity is known to decline (Laugier et al. 1991), but it is not prognostic. HDL has isolated prognostic evidence for a long lifespan for 18 M male mice only, possibly reflecting its generally accepted role of protecting blood vessels. Notable biomarkers of age going up longitudinally are the liver enzyme ALT (in males), and CO_2. Increased values of ALT are known to indicate damaged liver cells, and we indeed find isolated prognostic evidence for a short lifespan of 6 M females with high ALT values. The increased CO_2 in old mice likely reflects loss of ventilation capacity and hence decrease in pulmonary CO_2 expiration, with corresponding respiratory acidosis. Known pulmonary conditions in inbred strains of mice include hyalinosis and acidophilic macrophage pneumonia, which may be subclinical or fatal, and increase with incidence with age (Hoenerhoff et al. 2006) as do pulmonary neoplasias. Such an indicator of ‘lung damage’ late in life is not, however, prognostic. Notable biomarkers of age going down longitudinally are

phosphate concentrations and lipase activity, as well as HDL. Phosphate has isolated prognostic evidence for a short lifespan for 6 M male mice only, and high values are indeed implicated in ageing (Kuro-o 2010). The similarity between female serum phosphate and serum calcium effects might be expected because of the tight co-regulation of these two ions (Peacock 2010). It is not clear why there is no co-indication for phosphate in females, though, but this may reflect sex specific differences in renal calcium clearance and reabsorption (Lee et al. 2009).

Role-switching biomarkers based on prognostic evidence alone. In the manuscript, T4 is described as the single marker suggesting a role switch based on prognostic data alone. The other role-switching markers from Table 1, based on validated longitudinal as well as prognostic evidence, are missing significance if we only consider prognostic evidence. However, *no observations of statistical significance* are in conflict with the proposed role switches, with the exception of CHr, which predicts a low lifespan at 24 months, while a role switch would suggest that it predicts a long lifespan at this time point (Figs. 1 and 2). As indicated in the main text, however, data for 24 months are based on few strains, 11 in this case. Regarding the other markers, iron predicts a low lifespan at 6 and 12 months, but the prediction is insignificant at 18 months (Fig. 5); BMI (Suppl. Figure 2) predicts a low life span at 6 months (also at 12 months for females), but the prediction is insignificant at 20 months and HR (heart rate, Suppl. Figure 9) predicts a low life span at 6 months, but the predictions are insignificant at 12 and 20 months.

Supplementary Discussion.

Elevation of neutrophil numbers in younger mice is an observation not discussed in the main text. It may be due to causes such as aggressive behavior and wounding, early stages of rectal prolapse, or periodontal infections caused by food fragments or wear and breakage of the incisors. The propensity for the latter infections shows a genetic predisposition in mice but is clearly also dependent on husbandry conditions (Baker & Roopenian 2002). However, there is clear epidemiological data in man that chronic periodontal disease (PD), for example, is prognostic for major systemic degenerative diseases, such as atherosclerosis, and reduction in life expectancy in man (Xu & Lu 2011), consistent with the effects of chronic systemic inflammation as evidenced by elevation of inflammation markers (high C-reactive protein, white cell count and fibrinogen) in men with severe PD compared to men without PD.

Corroborating observations in human. For inbred mouse strains, the average lifespan of the strain closely reflects the lifespan of each individual mouse from that strain because of genetic identity and environmental homogeneity. (A panel of, for example, 30 inbred strains can therefore be treated like a panel of 30 genetically highly diverse individual humans with each measurement being made in multiple replicates for each individual.) No such estimation of lifespan is possible for human, so human cross-sectional studies do not allow correlation of lifespan with marker measurements. On the other hand, longitudinal studies in human rarely cover entire lifespans like mouse data do. They are difficult to standardize, too, because measurement protocols need to be kept consistent over more than half a century. Thus, we are not aware of any lifelong longitudinal studies where the overlap with the Jackson lab data is more than minimal (such as, e.g., BMI measurements). Therefore, validation of our markers in human is anecdotal, expanding upon the references already provided in the manuscript. From the literature we collected the following, ordered by biomarker as in Table 1, and including also references for other mammals.

- ***B cells, and lymphocytes*** in general, are affected by 'immunosenescence', due to thymic involution that is observed in many mammalian species including human (Malaguarnera et al. 2001; Franceschi et al. 2000; Maue et al. 2009).
- Low values of ***red blood cells, hemoglobin and hematocrit*** are signs of anemia, see, e.g., (Andrews 2000).
- The anti-inflammatory effects of ***magnesium*** are described in, e.g., (Barbagallo et al. 2009), with a focus on human, but including a few references to rodents. For rodents, we also refer to (Tam et al. 2003).
- The increase of ***neutrophil*** numbers indicates 'inflammaging', a process best studied in humans (Franceschi et al. 2000), but conserved evolutionarily (Salminen et al. 2008).

- On one hand, ***iron*** is implicated in anemia in humans and rodents alike (Andrews 2000). On the other hand, it is associated with oxidative stress and damage also in human (Kell 2009; Andrews 2000). Moreover, overall high iron load is known to be a risk factor for myocardial infarction (Tuomainen et al. 1998) and type 2 diabetes (Bao et al. 2012) in human populations.
- Regarding ***thyroxine*** (T4), a recent review (Bowers et al. 2013) connects thyroid hormone status with metabolism and also with oxidative damage and inflammation.
- A high ***body-mass index*** is considered disadvantageous, but for older people, a slightly higher BMI (not obesity) has advantageous effects, possibly due to the higher robustness against disease it affords (Auyeung & Lee 2010; Flegal et al. 2013).

References.

Aherrahrou Z, Doehring LC, Ehlers E-M, Liptau H, Depping R, Linsel-Nitschke P, Kaczmarek PM, Erdmann J & Schunkert H (2008) An alternative splice variant in Abcc6, the gene causing dystrophic calcification, leads to protein deficiency in C3H/He mice. *J. Biol. Chem.* 283, 7608–15.

Andrews NC (2000) Iron metabolism: iron deficiency and iron overload. *Annu. Rev. Genomics Hum. Genet.* 1, 75–98.

Auyeung T & Lee J (2010) Survival in older men may benefit from being slightly overweight and centrally obese—a 5-year follow-up study in 4,000 older adults using DXA. *Journals …* 65, 99–104.

Bao W, Rong Y, Rong S & Liu L (2012) Dietary iron intake, body iron stores, and the risk of type 2 diabetes: a systematic review and meta-analysis. *BMC Med.* 10, 119.

Barbagallo M, Belvedere M & Dominguez L (2009) Magnesium homeostasis and aging. *Magnes. Res.* 22, 235–246.

Berndt A, Li Q, Potter CS, Liang Y, Silva KA, Kennedy V, Uitto J & Sundberg JP (2013) A single-nucleotide polymorphism in the Abcc6 gene associates with connective tissue mineralization in mice similar to targeted models for pseudoxanthoma elasticum. *J. Invest. Dermatol.* 133, 833–6.

Bolland MJ, Avenell A, Baron JA, Grey A, MacLennan GS, Gamble GD & Reid IR (2010) Effect of calcium supplements on risk of myocardial infarction and cardiovascular events: meta-analysis. *BMJ* 341.

Bowers J, Terrien J, Clerget-Froidevaux MS, Gothié JD, Rozing MP, Westendorp RGJ, van Heemst D & Demeneix BA (2013) Thyroid hormone signaling and homeostasis during aging. *Endocr. Rev.* 34, 556–89.

Bristow SM, Bolland MJ, Maclennan GS, Avenell A, Grey A, Gamble GD & Reid IR (2013) Calcium supplements and cancer risk: a meta-analysis of randomised controlled trials. *Br. J. Nutr.* 110, 1384–93.

Flegal KM, Kit BK, Orpana H & Graubard BI (2013) Association of all-cause mortality with overweight and obesity using standard body mass index categories: a systematic review and meta-analysis. *JAMA* 309, 71–82.

Franceschi C, Bonafè M & Valensin S (2000) Human immunosenescence: the prevailing of innate immunity, the failing of clonotypic immunity, and the filling of immunological space. *Vaccine* 18, 1717–20.

Hoenerhoff MJ, Starost MF & Ward JM (2006) Eosinophilic crystalline pneumonia as a major cause of death in 129S4/SvJae mice. *Vet. Pathol.* 43, 682–8.

Kell DB (2009) Iron behaving badly: inappropriate iron chelation as a major contributor to the aetiology of vascular and other progressive inflammatory and degenerative diseases. *BMC Med. Genomics* 2, 2.

Kuro-o M (2010) A potential link between phosphate and aging--lessons from Klotho-deficient mice. *Mech. Ageing Dev.* 131, 270–5.

Laugier R, Bernard JP, Berthezene P & Dupuy P (1991) Changes in pancreatic exocrine secretion with age: pancreatic exocrine secretion does decrease in the elderly. *Digestion* 50, 202–11.

Lee G-S, Choi K-C & Jeung E-B (2009) K+-dependent Na+/Ca2+ exchanger 3 is involved in renal active calcium transport and is differentially expressed in the mouse kidney. *Am. J. Physiol. Renal Physiol.* 297, F371–9.

Malaguarnera L, Ferlito L, Imbesi RM, Gulizia GS, Di Mauro S, Maugeri D, Malaguarnera M & Messina A (2001) Immunosenescence: a review. *Arch. Gerontol. Geriatr.* 32, 1–14.

Maue AC, Yager EJ, Swain SL, Woodland DL, Blackman MA & Haynes L (2009) T-cell immunosenescence: lessons learned from mouse models of aging. *Trends Immunol.* 30, 301–5.

Peacock M (2010) Calcium metabolism in health and disease. *Clin. J. Am. Soc. Nephrol.* 5 Suppl 1, S23–30.

Salminen A, Huuskonen J, Ojala J, Kauppinen A, Kaarniranta K & Suuronen T (2008) Activation of innate immunity system during aging: NF-kB signaling is the molecular culprit of inflamm-aging. *Ageing Res. Rev.* 7, 83–105.

Tam M, Gómez S, González-Gross M & Marcos A (2003) Possible roles of magnesium on the immune system. *Eur. J. Clin. Nutr.* 57, 1193–7.

Tuomainen TP, Punnonen K, Nyyssönen K & Salonen JT (1998) Association between body iron stores and the risk of acute myocardial infarction in men. *Circulation* 97, 1461–6.

Yuan R, Tsaih S, Petkova SB, Marin de Evsikova C, Xing S, Marion MA, Bogue MA, Mills KD, Peters L, Bult CJ, Rosen CJ, Sundberg JP, Harrison DE, Churchill GA & Paigen B (2009) Aging in inbred strains of mice: study design and interim report on median lifespans and circulating IGF1 levels. *Aging Cell* 8, 277–87.
