# Supplementary material for: Inbred mouse strains reveal biomarkers that are pro-longevity, antilongevity or role switching
Source: Aging Cell. 2014 May 23;13(4):729–38. doi: 10.1111/acel.12226 (PMC4326954; doi:10.1111/acel.12226)
Supplement: Supplementary file 2 — Data S1 Supplementary tables and figures: linear regression and correlation charts. [file acel0013-0729-sd2.pdf]

Supplementary Data I

Supplementary Tables & Figures, Linear Regression and Correlation Charts

1. Nathan Shock Center datasets from the Mouse Phenome Database ____ 2
2. Abbreviations ____ 2
3. Biomarker Classification ____ 4
4. Ackert1 - Bone mineral density and body composition ____ 5
   4.1. Linear regression ____ 5
   4.2. Life expectancy correlation analysis ____ 6
5. Korstanje1 - Urine albumin and creatinine ____ 7
   5.1. Linear regression ____ 7
   5.2. Life expectancy correlation analysis ____ 7
6. Mills1 - Chromosome instability and DNA Repair ____ 8
   6.1. Linear Regression ____ 8
   6.2. Life expectancy correlation analysis ____ 8
7. Linear Regression: Blood hematology in 30 inbred strains of mice (Dataset: Peters4) ____ 9
8. Linear Regression: Peripheral blood leukocytes (PBL profiles) in 32 inbred strains of mice (Dataset: Petkova1) ____ 9
9. Xing1 - Electrocardiogram ____ 10
   9.1. Linear Regression ____ 10
   9.2. Life expectancy correlation analysis ____ 10
10. Yuan1 - IGF-1 and bodyweight ____ 11
    10.1. Linear Regression ____ 11
    10.2. Life expectancy correlation analysis ____ 11
11. Linear regression: Blood chemistry for 32 inbred strains of mice (Dataset: Yuan3) ____ 12
12. Sample regression analyses ____ 12

1. Nathan Shock Center datasets from the Mouse Phenome Database

Data File	Study	N(strains)	6 M	12 M	18 M	20 M	24 M
Ackert1	Bone mineral density and body composition of 32 inbred strains of mice	32	X	X	-	X	-
Korstanje1	Urine albumin and creatinine in 30 inbred strains of mice	30	-	X	X	-	X
Mills1	Chromosome instability and apoptosis in 30 inbred strains of mice	30	X	X	-	X	-
Peters4	Blood hematology in 30 inbred strains of mice	30	X	X	X	-	X
Petkova1	Peripheral blood leukocytes (PBL profiles) in 32 inbred strains of mice	32	X	X	X	-	X
Xing1	Electrocardiogram for 29 inbred strains of mice	29	X	X	-	X	-
Yuan1	IGF-1 and body weight for 33 inbred strains of mice	33	X	X	X	-	-
Yuan3	Aging study: Blood chemistry for 32 inbred strains of mice	32	X	X	X	-	-
Yuan2	Lifespan and survival curves for 33 inbred strains of mice*	32	-	-	-	-	-
number of strains with individuals still alive at age of measurement:		female	32	32	29	26	11
		male	32	32	27	22	13

Supplementary Table 1: Nathan Shock Center datasets from the *Mouse Phenome Database*, including time points (age groups) available [X], and an indication of the colors used in the correlation charts accompanying this text. M: months. (*Despite the name of the study, Yuan2 data on life expectancies are available only for 32, not 33, inbred strains).

2. Abbreviations

In Dataset	Abbreviation used	Measurement [original units]
Ackert1	BL	body length (tip of nose to base of tail) [cm]
Ackert1	BMC	Bone mineral content (g)
Ackert1	BMD	Bone mineral density = BMC ÷ Bone-area [g/cm^2]
Ackert1	BMI	body mass index (BMI) [kg/m^2]
Ackert1	bone_area	Bone area (cm^2)
Ackert1	BW	body weight [g]
Ackert1	fatwt	weight of fat portion of body mass [g]
Ackert1	LTM	weight of lean tissue mass [g]
Ackert1	pctfat	percent fat [%]
Ackert1	total_area	total body area without head [cm^2]
Ackert1	TTM	total tissue mass [g]
Korstanje1	ACR	albumin-creatinine ratio (urine ACR) [mg/g]
Korstanje1	CREA	creatinine (urine CREA) [mg/dL]
Korstanje1	MA	microalbumin (urine MA) [mg/dL]
Mills1	all apoptotic	all apoptotic splenocytes [%] = early ap. spl. + late ap. spl.
Mills1	early_apoptotic	early apoptotic splenocytes [%]
Mills1	late_apoptotic	late apoptotic splenocytes [%]
Mills1	RBC micronucl	red blood cells (RBC) with micronuclei [%]
Mills1	retic micronucl	reticulocytes with micronuclei [%]
Mills1	all_micronucl	non-nucleated peripheral blood cells with micronuclei (retic+RBC) [%]
Peters4	nBASO	basophil count (BASO; units per volume x 10^3) [n/µL]
Peters4	pctBASO	basophil differential (percent of total WBC) [%]
Peters4	CHr	reticulocyte corpuscular hemoglobin (CHr) [pg]
Peters4	nEOS	eosinophil count (EOS; units per volume x 10^3) [n/µL]
Peters4	pctEOS	eosinophil differential (percent of total WBC) [%]
Peters4	HGB	hemoglobin (HGB) [g/dL]

In Dataset	Abbreviation used	Measurement [original units]
Peters4	nLUC	large unstained cells count (LUC; units per volume x 10^3) [n/µL]
Peters4	pctLUC	large unstained cell differential (percent of total WBC) [%]
Peters4	nLYMPH	lymphocyte count (LYMP; units per volume x 10^3) [n/µL]
Peters4	pctLYMPH	lymphocyte differential (percent of total WBC) [%]
Peters4	MCH	mean RBC corpuscular hemoglobin content (MCH) [pg]
Peters4	MCHC	mean RBC hemoglobin concentration (MCHC) [g/dL]
Peters4	MCV	mean RBC corpuscular volume (MCV) [fL]
Peters4	nMONO	monocyte count (MONO; units per volume x 10^3) [n/µL]
Peters4	pctMONO	monocyte differential (percent of total WBC) [%]
Peters4	MPV	mean platelet volume (MPV) [fL]
Peters4	nNEUT	neutrophil count (NEUT; units per volume x 10^3) [n/µL]
Peters4	pctNEUT	neutrophil differential (percent of total WBC) [%]
Peters4	pctHCT	hematocrit (HCT) [%]
Peters4	nPlt	platelet count (PLT; units per volume x 10^3) [n/µL]
Peters4	nRBC	red blood cell count (RBC; per volume x 10^6) [n/µL]
Peters4	nRetic	reticulocyte count (Retic; units per volume x 10^9) [n/L]
Peters4	pctRetic	reticulocyte differential (percent of total RBC) [%]
Peters4	nWBC	white blood cell count (WBC; per volume x 10^3) [n/µL]
Petkova1	pctB-cells	B cells [% of LYM]
Petkova1	pctCD4_all	CD4 T cells [% of LYM]
Petkova1	pctCD4_effector	CD4 T cell subtypes [% of CD4_all] - effector cells
Petkova1	pctCD4 mem central	CD4 T cell subtypes [% of CD4_all] - central memory cells
Petkova1	pctCD4 mem effector	CD4 T cell subtypes [% of CD4_all] - effector memory cells
Petkova1	pctCD4 naive	CD4 T cell subtypes [% of CD4_all] - naive cells
Petkova1	pctCD8 all	CD8 T cells [% of LYM]
Petkova1	pctCD8 effector	CD8 T cell subtypes [% of CD8_all] - effector cells
Petkova1	pctCD8 mem central	CD8 T cell subtypes [% of CD8_all] - central memory cells
Petkova1	pctCD8 mem effector	CD8 T cell subtypes [% of CD8_all] - effector memory cells
Petkova1	pctCD8 naive	CD8 T cell subtypes [% of CD8_all] - naive cells
Petkova1	pctEOS	eosinophil differential [% of WBC]
Petkova1	pctLYMPH	lymphocyte differential [% of WBC]
Petkova1	pctMONO	monocyte differential [% of WBC]
Petkova1	pctNEUT	neutrophil differential [% of WBC]
Petkova1	pctNK	NK cells [% of LYM]
Petkova1	nWBC	white blood cell count (WBC) [$n \cdot 10^3/\mu L$]
Xing1	HR	heart rate [n/min]
Xing1	PR	intervals [ms]
Xing1	QRS	intervals [ms]
Xing1	QT	intervals [ms]
Xing1	RR	intervals [ms]
Xing1	ST	intervals [ms]
Yuan1	BW	body weight [g]
Yuan1	IGF1	insulin-like growth factor 1 (serum IGF-1) [ng/mL]
Yuan3	ALB	albumin (plasma Alb) [g/dL]
Yuan3	ALP	alkaline phosphatase (serum ALP) [IU/L]
Yuan3	ALT	alanine aminotransferase (plasma ALT) [IU/L]
Yuan3	BUN	blood urea nitrogen (plasma BUN) [mg/dL]
Yuan3	Ca	calcium (serum Ca) [mg/dL]

In Dataset	Abbreviation used	Measurement [original units]
Yuan3	Cl	chloride (serum Cl) [mmol/L]
Yuan3	CO2	dissolved-ionized carbon dioxide (CO_2) [mmol/L]
Yuan3	Fe	iron (serum Fe) [mmol/L]
Yuan3	HDL	HDL cholesterol (plasma HDL) [mg/dL]
Yuan3	K	potassium (serum K) [mmol/L]
Yuan3	LIP	lipase (plasma LIP) [U/L]
Yuan3	Mg	magnesium (serum Mg) [mmol/L]
Yuan3	Na	sodium (serum Na) [mmol/L]
Yuan3	Phos	phosphorus (serum P) [mg/dL]
Yuan3	T4	thyroxine (serum T4) [µg/dL]
Yuan3	TBIL	bilirubin (serum TBIL) [µmol/L]
Yuan3	TP	total protein (plasma TP) [g/dL]

Supplementary Table 2: Abbreviations

3. Biomarker Classification

**Prognostic evidence**	**Evidence:** **prognostic for …**	→	**Resulting classification**		**Known effect on lifespan (explanatory)**
Case A	long life span	→	(early) pro-longevity		positive (at least early)
Case B	short life span	→	(early) anti-longevity		negative (at least early)
**Prognostic evidence, role-changing biomarker**	**Evidence:** **prognostic for …**	→	**Resulting classification**		**Known effect on lifespan (explanatory)**
Case C	long life span (early in life)<br>short life span (late in life)	→	(early) pro-longevity,<br>(late) anti-longevity		positive (early),<br>negative (late)
Case D	short life span (early in life)<br>long life span (late in life)	→	(early) anti-longevity,<br>(late) pro-longevity		negative (early),<br>positive (late)
**Longitudinal evidence**	**Evidence:** **Longitudinal trend**	**+**	**Known effect on lifespan**	→	**Resulting classification**
Case E	up	+	negative (at least late)	→	(late) anti-longevity
Case F	down	+	positive (at least late)	→	(late) pro-longevity

Supplementary Table 3: Biomarker classification, using prognostic evidence (cases A-D) or longitudinal evidence (cases E, F). Prognostic evidence (cases A, B) is usually consistent across age groups (for exceptions see cases C, D). Prognostic evidence of high biomarker values in terms of life span may be *explained* by their known effects on lifespan. These are usually early effects, with consequences later in life; hence prognostic. Prognostic evidence of role-changing biomarkers is found in cases C, D. Extending upon cases A and B, a role-switch of biomarkers is derived from inconsistent prognostic data, e.g., predicting short life span for 6 M data (early in life), and long life span for 12 M/18 M data (late in life). In the dataset we analyzed, only one example (of the second case) was found. In this case, late in life, the biomarker is still prognostic, with consequences even later. Longitudinal evidence (cases E, F) *must be validated* by late known matching effects on life span. Specifically, a biomarker of age is also a biomarker of ageing (anti-longevity) if it triggers a deleterious effect.

4. Ackert1 - Bone mineral density and body composition

4.1. Linear regression

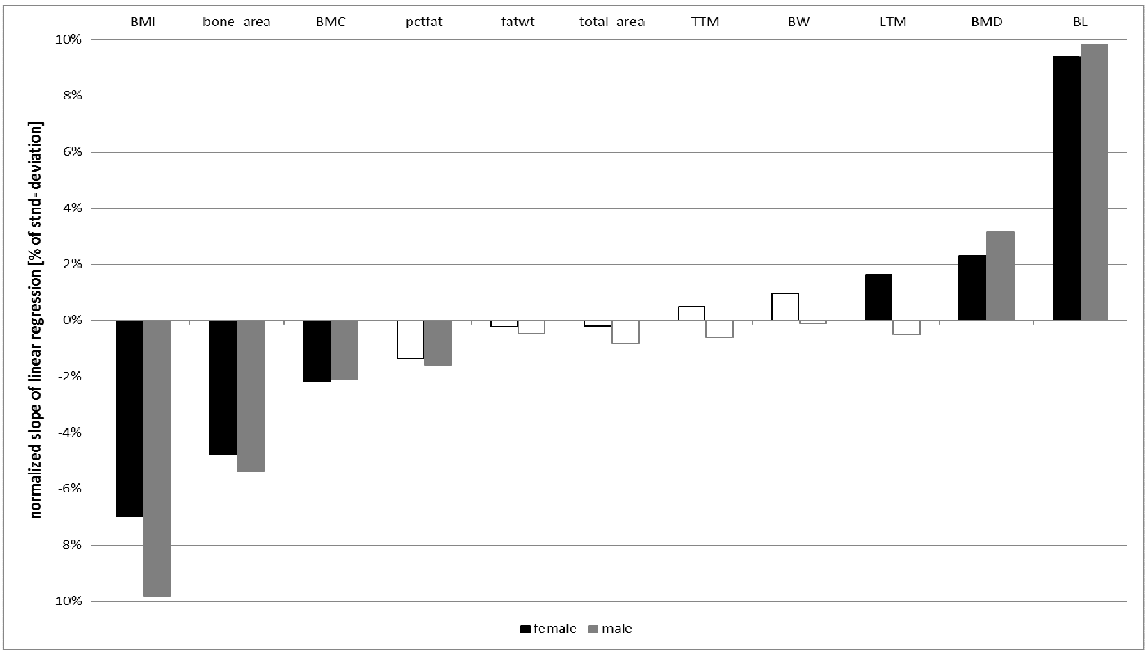

Supplementary Figure 1: Linear Regression on Bone mineral density and body composition (dataset: Ackert1) sorted ascending by female values; statistically insignificant values are presented by open bars. *Abbreviations of all features are indicated in* **Supplementary Table 2**.

4.2. Life expectancy correlation analysis

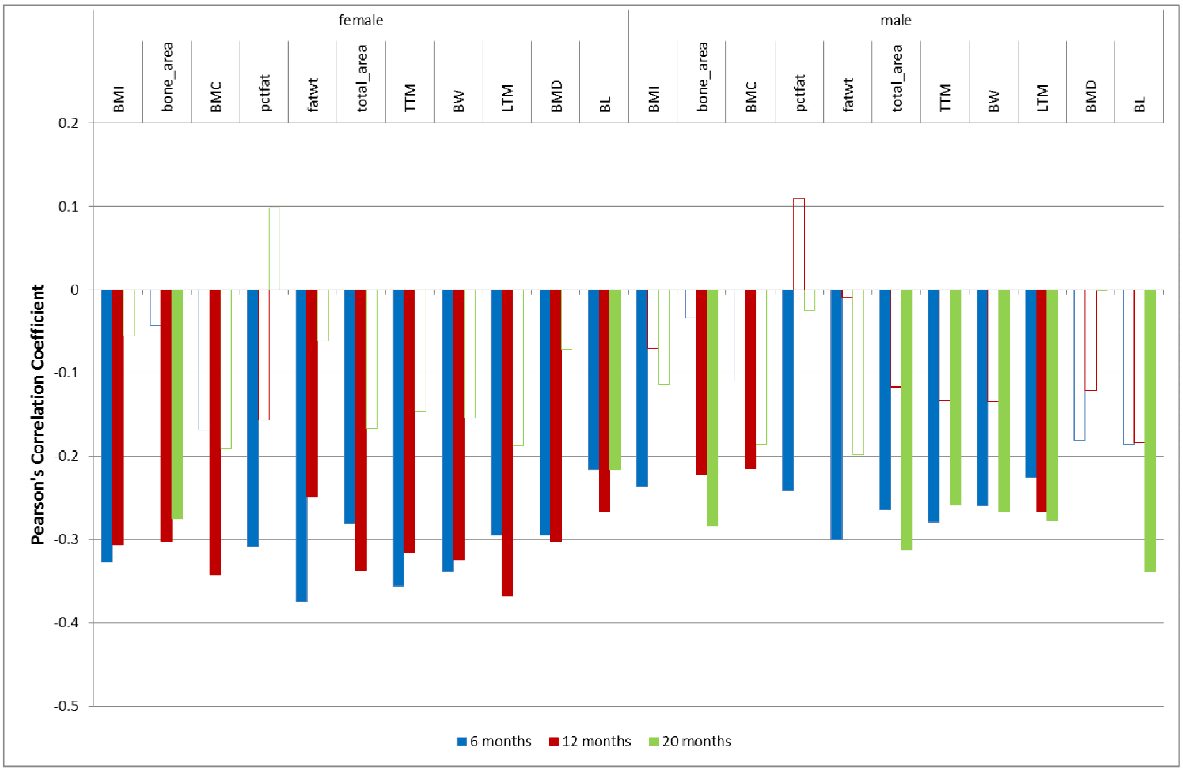

Supplementary Figure 2: Correlation Analysis - Bone mineral density and body composition (dataset: Ackert1); irrelevant values (<│0.2│ or / and p-value < 0.05) are presented by open bars. *Abbreviations of all features are indicated in* **Supplementary Table 2**.

5. Korstanje1 - Urine albumin and creatinine

5.1. Linear regression

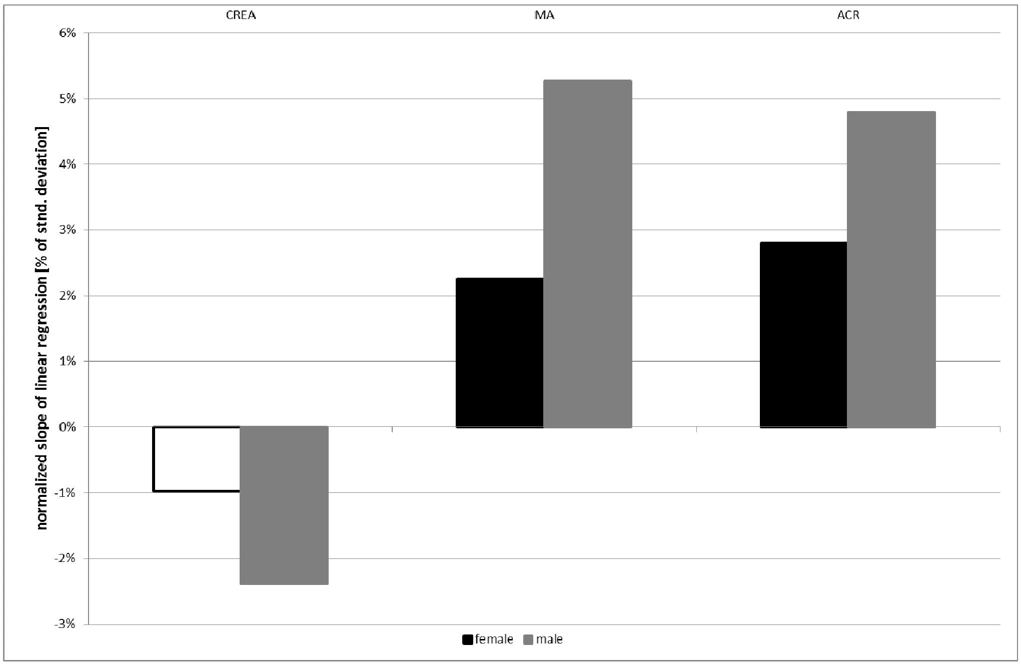

Supplementary Figure 3: Linear Regression on Urine Albumin and Creatinine (dataset: Korstanje1) sorted ascending by female values; statistically insignificant values are presented by open bars. *Abbreviations of all features are indicated in* **Supplementary Table 2**.

5.2. Life expectancy correlation analysis

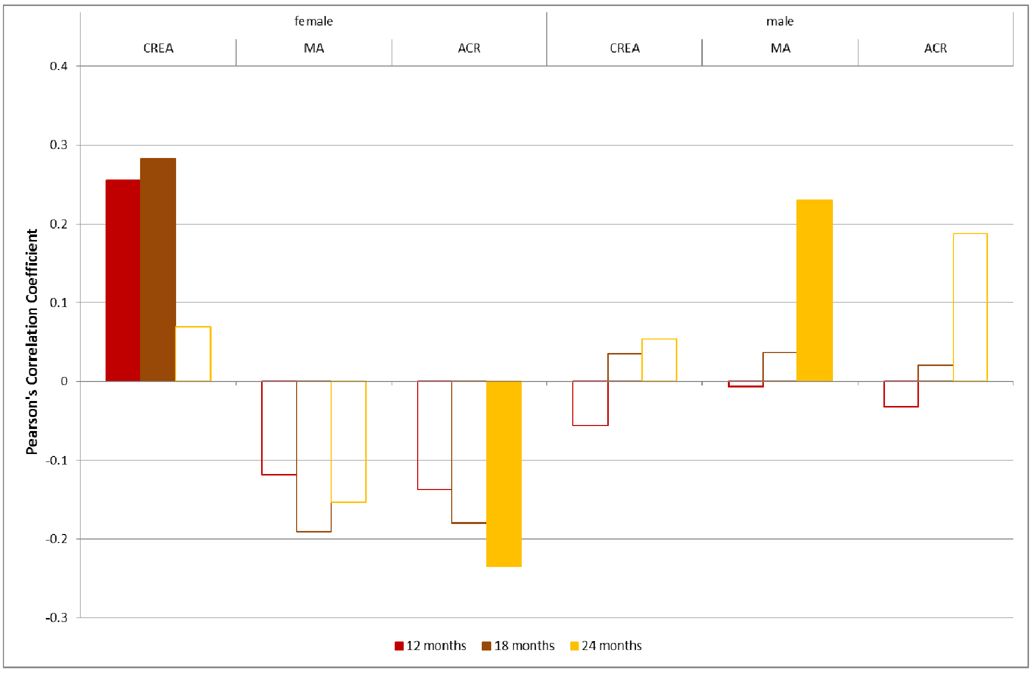

Supplementary Figure 4: Correlation Analysis - Urine Albumin and Creatinine (dataset: Korstanje1); irrelevant values (<│0.2│ or / and p-value < 0.05) are presented by open bars. *Abbreviations of all features are indicated in* **Supplementary Table 2**.

6. Mills1 - Chromosome instability and DNA Repair

6.1. Linear Regression

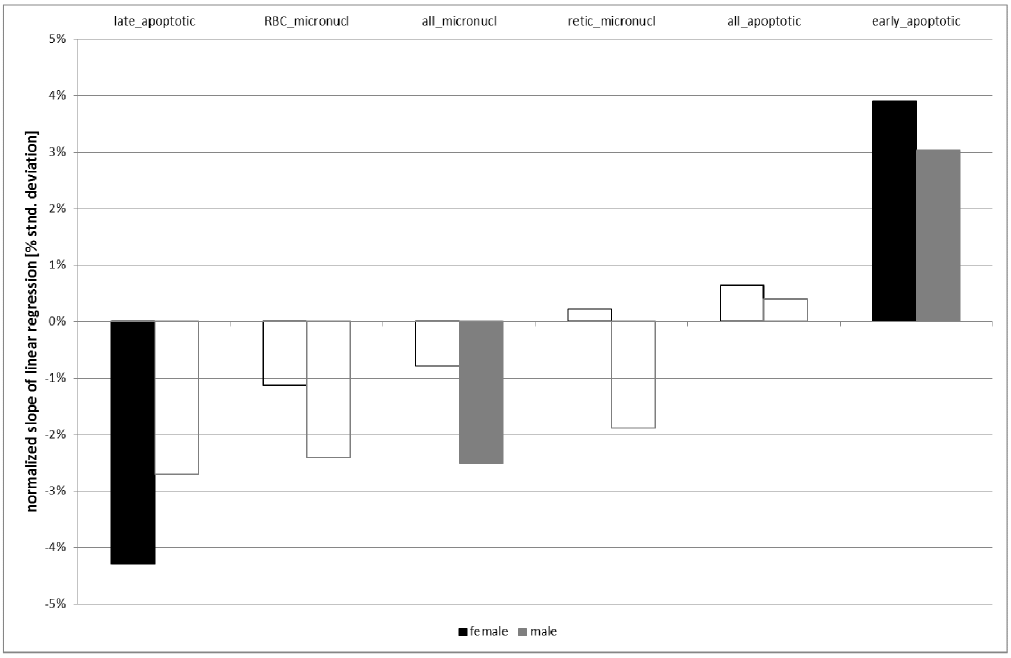

Supplementary Figure 5: Linear Regression on - Chromosome Instability and DNA Repair (dataset: Mills1) sorted ascending by female values; statistically insignificant values are presented by open bars. *Abbreviations of all features are indicated in* **Supplementary Table 2**.

6.2. Life expectancy correlation analysis

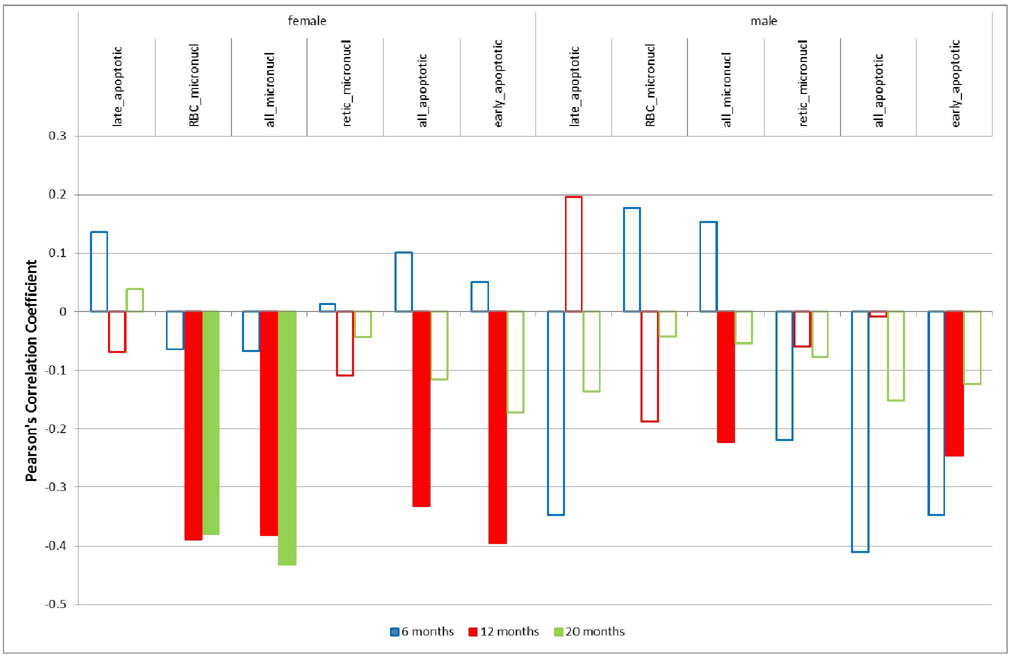

Supplementary Figure 6: Correlation Analysis - Chromosome Instability and DNA Repair (dataset: Mills1); irrelevant values (<│0.2│ or / and p-value < 0.05) are presented by open bars. *Abbreviations of all features are indicated in* **Supplementary Table 2**.

7. Linear Regression: Blood hematology in 30 inbred strains of mice (Dataset: Peters4)

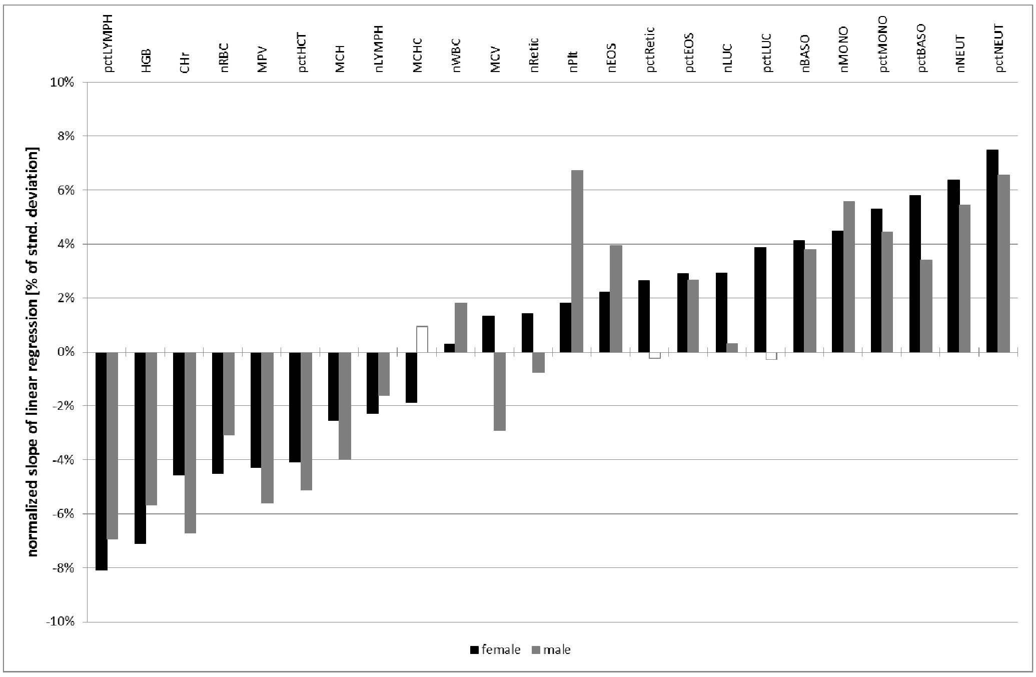

Supplementary Figure 7: Linear Regression on Blood cell count data (dataset: Peters4) sorted ascending by female values; statistically insignificant values are represented by open bars. *Abbreviations of all features are given in* **Supplementary Table 2.**

8. Linear Regression: Peripheral blood leukocytes (PBL profiles) in 32 inbred strains of mice (Dataset: Petkova1)

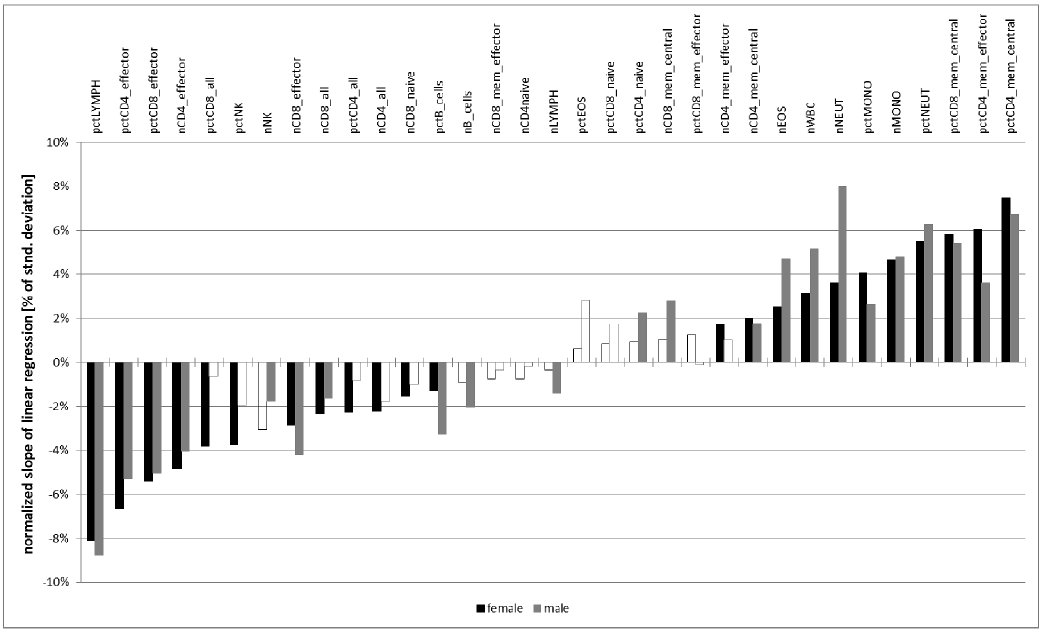

Supplementary Figure 8: Linear Regression on leukocyte data (dataset: Petkova1) sorted ascending by female values; statistically insignificant values are presented by open bars. *Abbreviations of all features are given in* **Supplementary Table 2.**

9. Xing1 - Electrocardiogram

9.1. Linear Regression

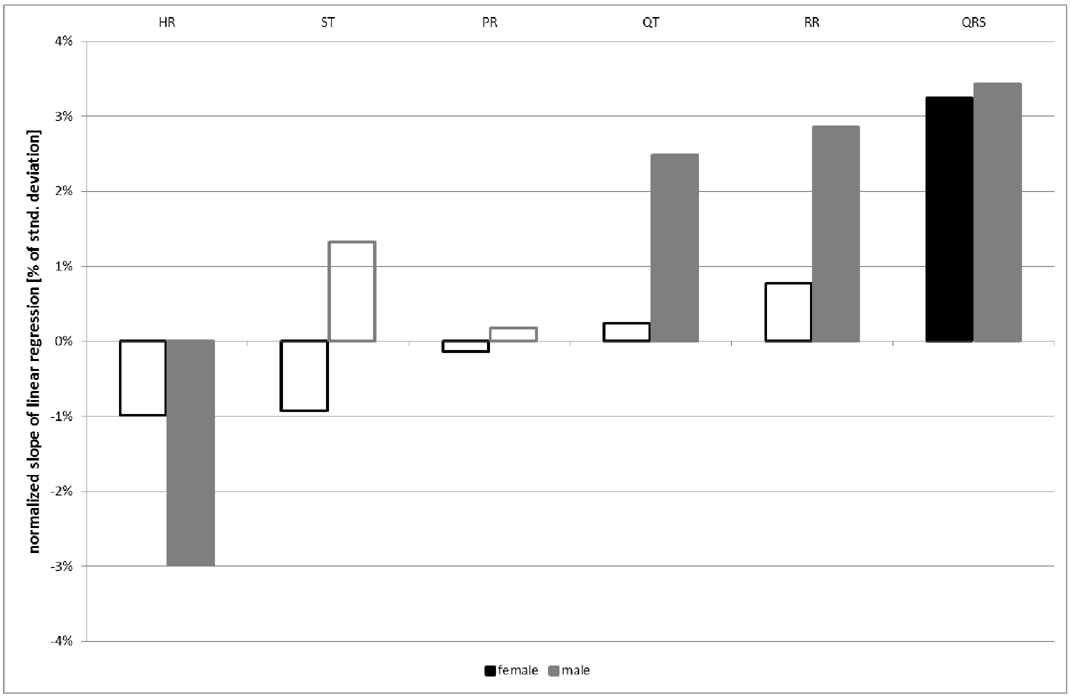

Supplementary Figure 9: Linear Regression on Electrocardiogram data (dataset: Xing1) sorted ascending by female values; statistically insignificant values are presented by open bars. Abbreviations of all features are indicated in **Supplementary Table 2**.

9.2. Life expectancy correlation analysis

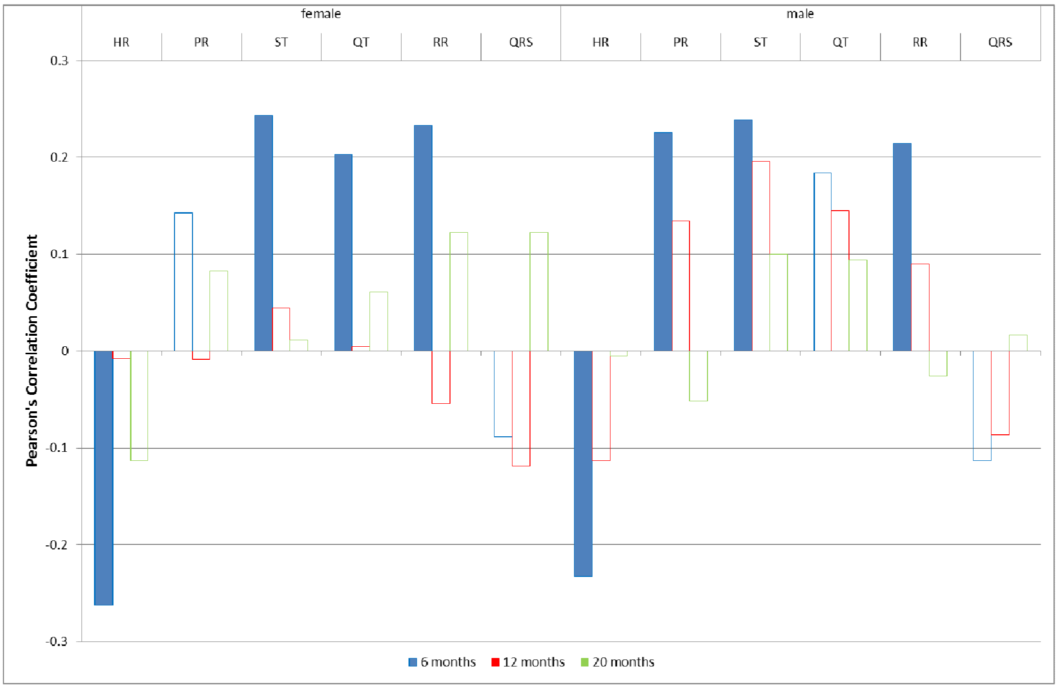

Supplementary Figure 10: Correlation Analysis - Electrocardiogram (dataset: Xing1); irrelevant values (<│0.2│ or / and p-value < 0.05) are presented by open bars. *Abbreviations of all features are indicated in* **Supplementary Table 2**.

10. Yuan1 - IGF-1 and bodyweight

10.1. Linear Regression

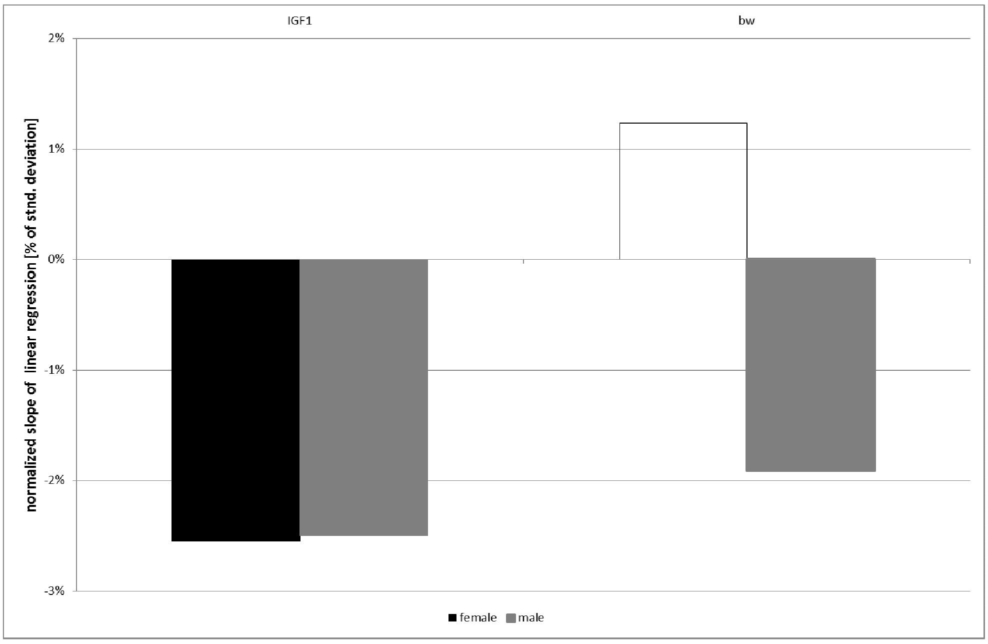

Supplementary Figure 11: Linear Regression on - IGF-1, bodyweight data (dataset: Yuan1) sorted ascending by female values; statistically insignificant values are presented by open bars. *Abbreviations of all features are indicated in* **Supplementary Table 2.**

10.2. Life expectancy correlation analysis

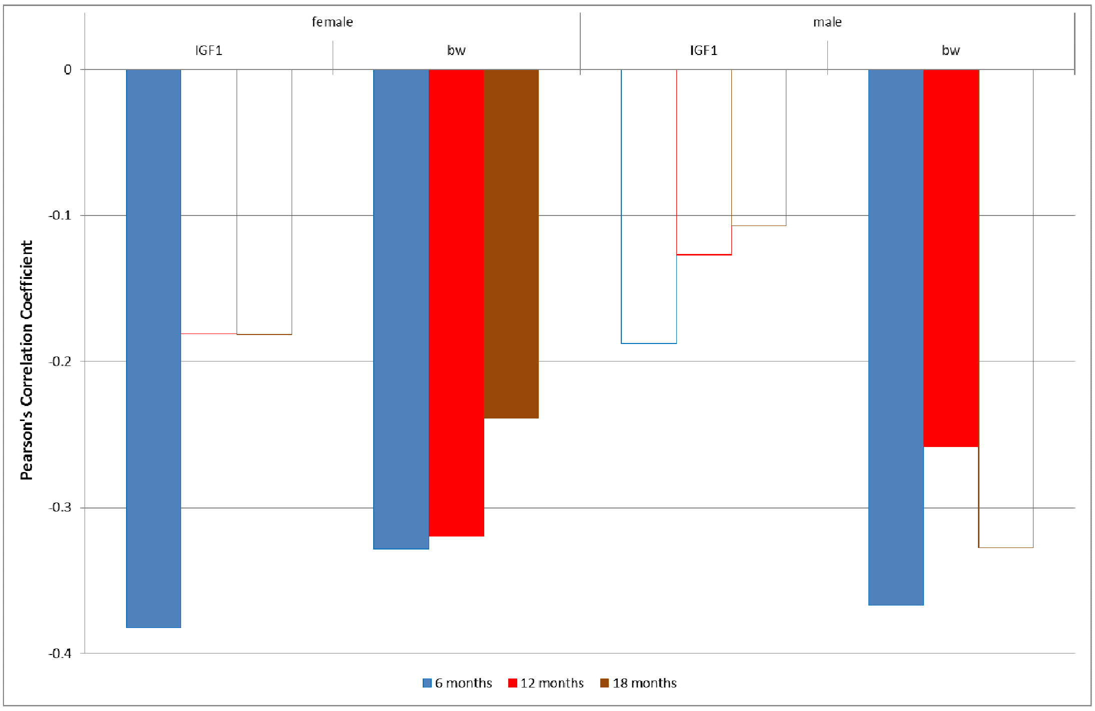

Supplementary Figure 12: Correlation Analysis - IGF-1, bodyweight (dataset: Yuan1); statistically insignificant values (<│0.2│ or / and p-value < 0.05) are presented by open bars (strains excluded: Pohn/Deh). *Abbreviations of all features are indicated in* **Supplementary Table 2***.*

11. Linear regression: Blood chemistry for 32 inbred strains of mice (Dataset: Yuan3)

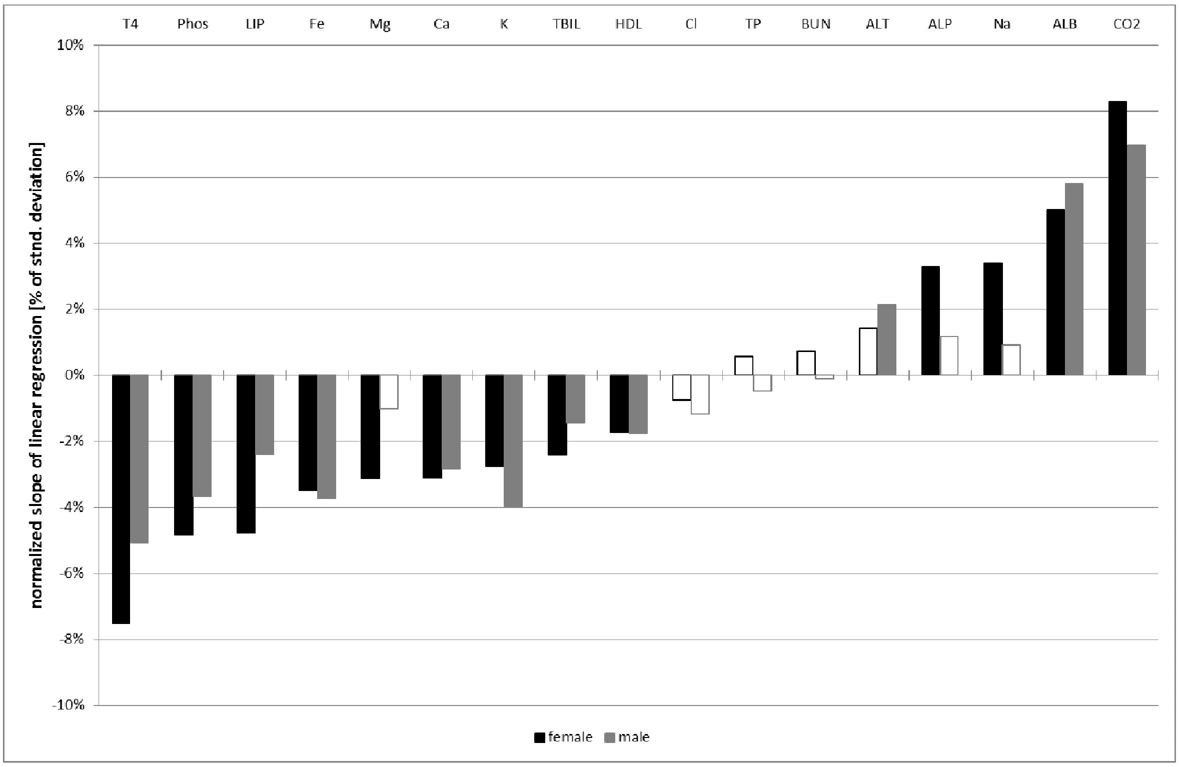

Supplementary Figure 13: Linear Regression on Blood Chemistry (dataset: Yuan3) sorted ascending by female values; statistically insignificant values are presented by open bars. *Abbreviations of all features are indicated in* **Supplementary Table 2.**

12. Sample regression analyses

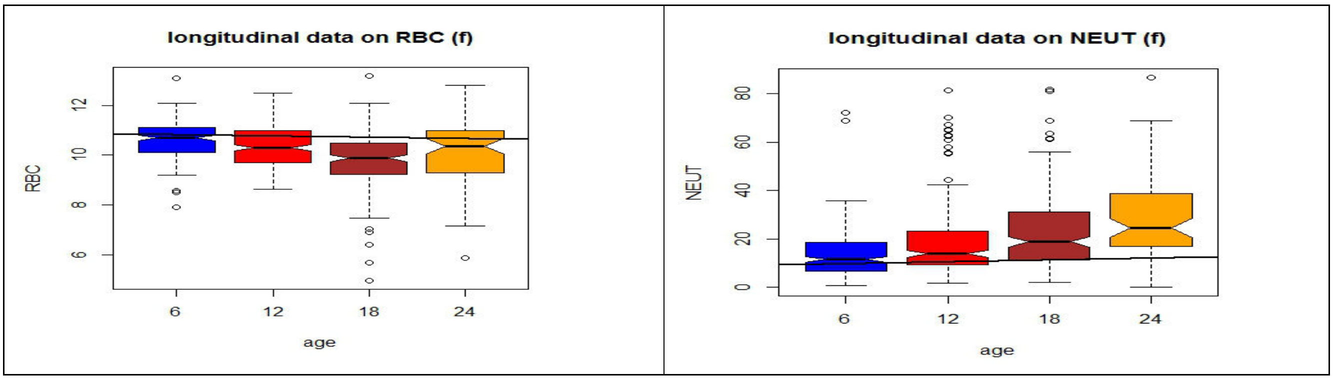

Supplementary Figure 14: Sample regression analyses are given for the features RBC (nRBC, red blood cell count, regression slope: -0.045, p-value: 3.11×10^{-13}) and NEUT (nNEUT, neutrophil count, regression slope: 0.064, p-value: 9.48×10^{-26}).
