# Supplementary material for: Inbred mouse strains reveal biomarkers that are pro-longevity, antilongevity or role switching
Source: Aging Cell. 2014 May 23;13(4):729–38. doi: 10.1111/acel.12226 (PMC4326954; doi:10.1111/acel.12226)
Supplement: Supplementary file 3 — Data S2 Linear regression and correlations charts excluding short-lived strains. [file acel0013-0729-sd3.pdf]

Supplement II

Simple linear regression and life span correlation analyses excluding short-lived strains (life expectancy < 600 days)

1 Ackert1 – Body Composition 2

1.1 Regression Analysis – Ackert1 2

1.2 Correlation Analysis – Ackert1 3

2 Peters4 – Blood Count 4

2.1 Regression Analysis – Peters4 4

2.2 Correlation Analysis – Peters4 5

2.2.1 Correlation Analysis – Female – Peters4 5

2.2.2 Correlation Analysis – Male – Peters4 6

3 Petkova1 - Leukocytes 7

3.1 Regression Analysis - Petkova1 7

3.2 Correlation Analysis - Petkova1 8

3.2.1 Correlation Analysis – Female – Petkova1 8

3.2.2 Correlation Analysis – Male – Petkova1 9

4 Yuan1 - IGF 10

4.1 Regression Analysis – Yuan1 10

4.2 Correlation Analysis – Yuan1 11

5 Yuan3 – Blood Chemistry 12

5.1 Regression Analysis – Yuan3 12

5.2 Correlation Analysis – Yuan3 13

5.2.1 Correlation Analysis – Female – Yuan3 13

5.2.2 Correlation Analysis – Male – Yuan3 14

1 Ackert1 – Body Composition

1.1 Regression Analysis – Ackert1

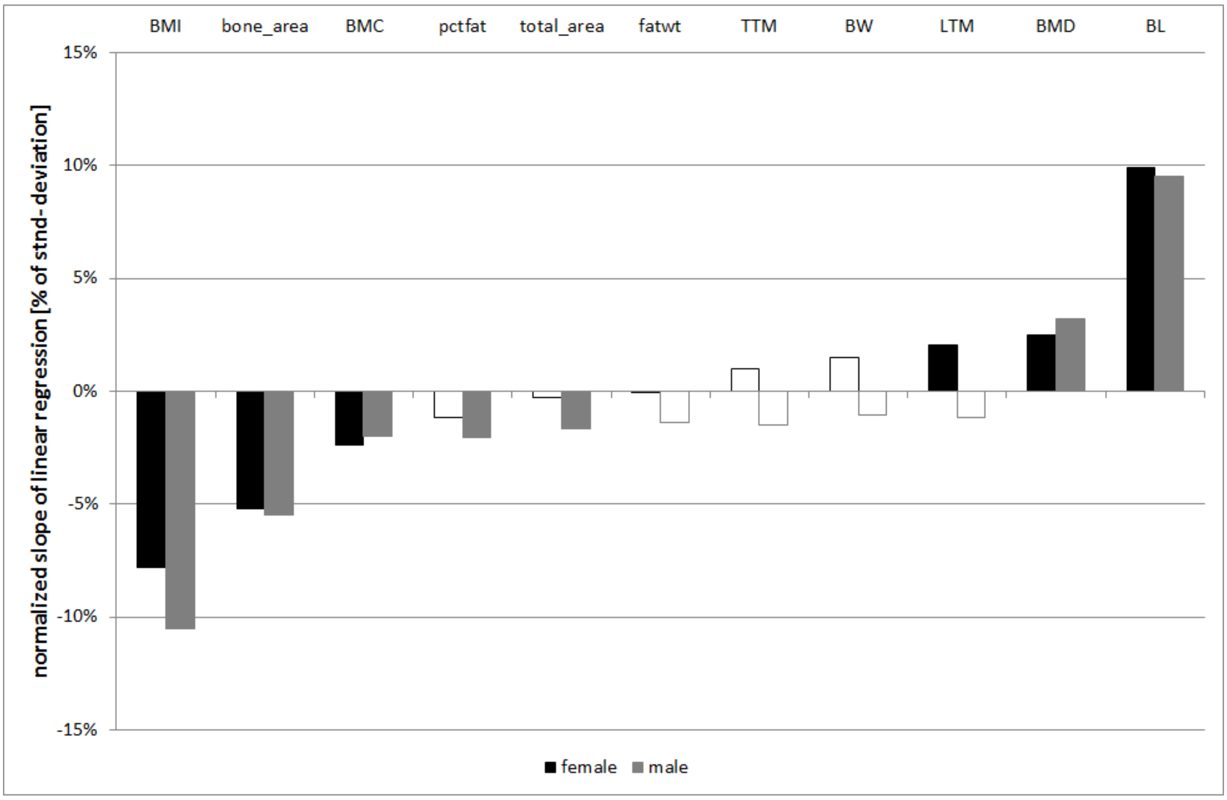

Suppl.II-Figure 1: Linear Regression on bone mineral density and body composition (dataset: Ackert1); statistically insignificant values are presented by open bars.

1.2 *Correlation Analysis – Ackert1*

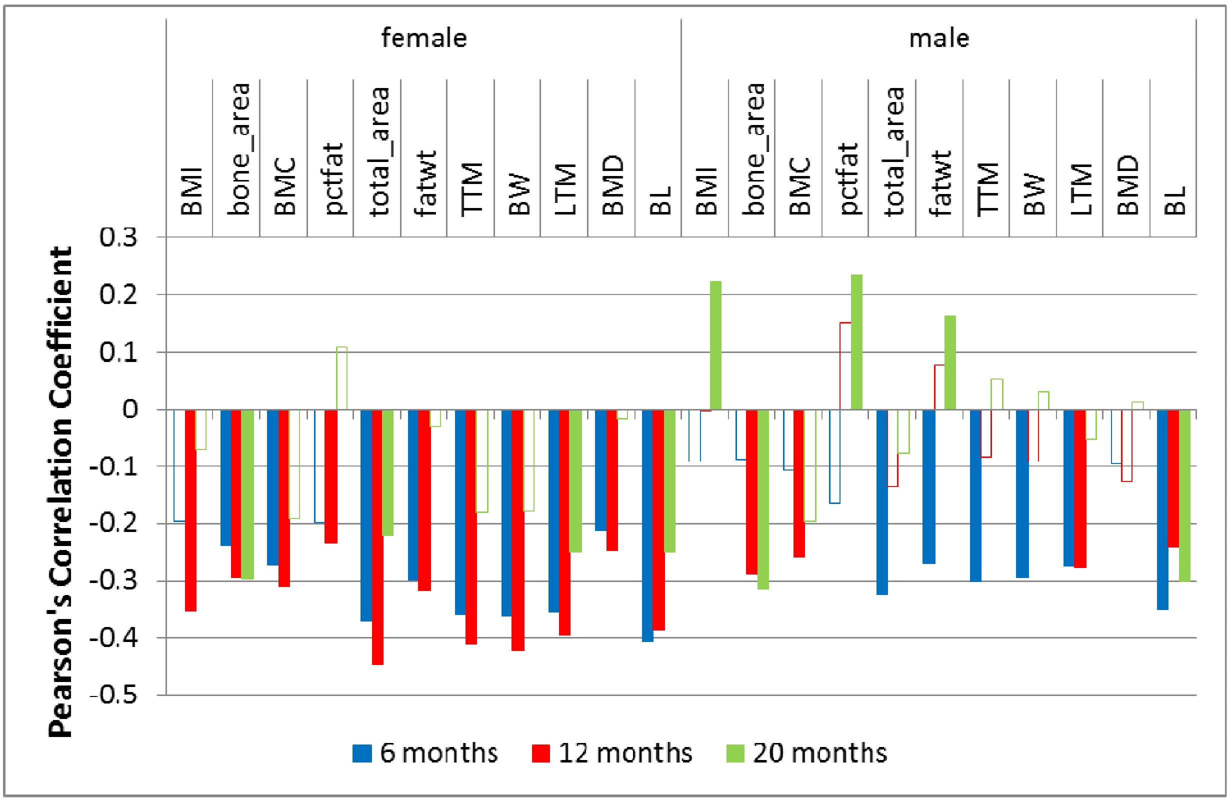

Suppl.II-Figure 2: Correlation Analysis on bone mineral density and body composition (dataset: Ackert1); statistically insignificant values (<|0.2| or / and p-value < 0.05) are presented by open bars.

2 Peters4 – Blood Count

2.1 Regression Analysis – Peters4

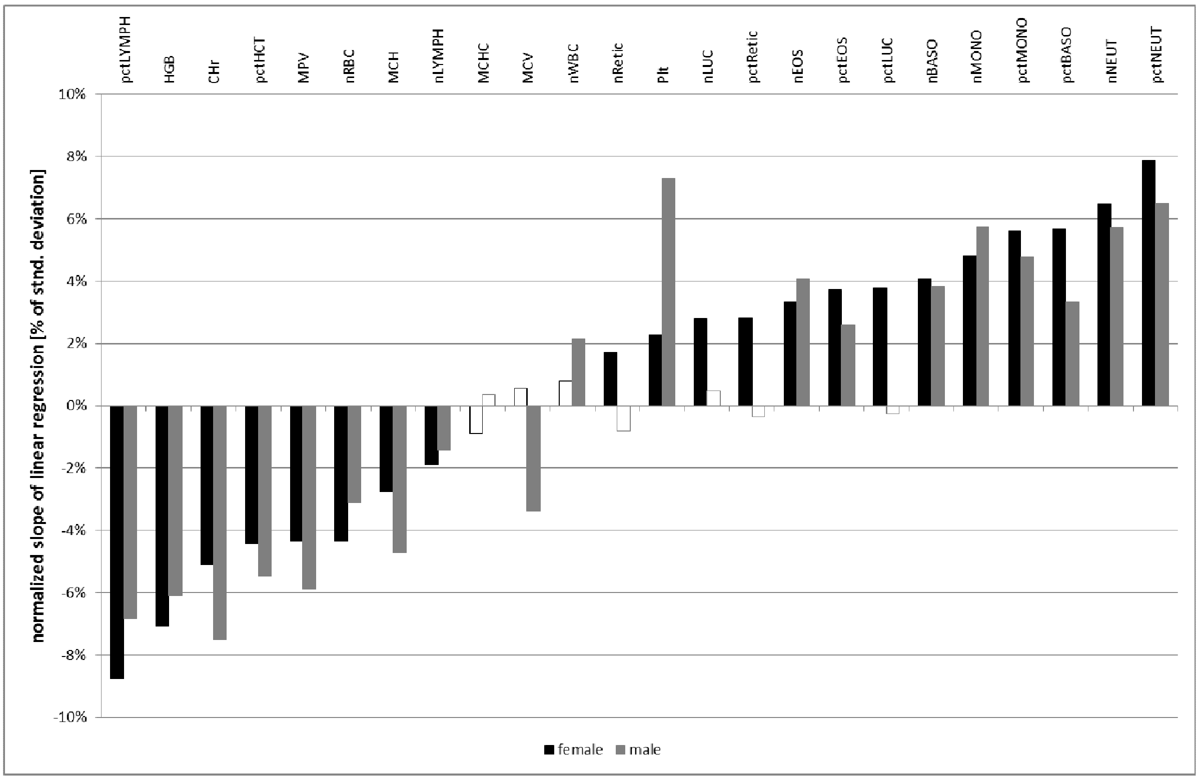

Suppl.II-Figure 3: Linear Regression on blood cell count data (dataset: Peters4); statistically insignificant values are represented by open bars.

2.2 *Correlation Analysis – Peters4*

2.2.1 Correlation Analysis – Female – Peters4

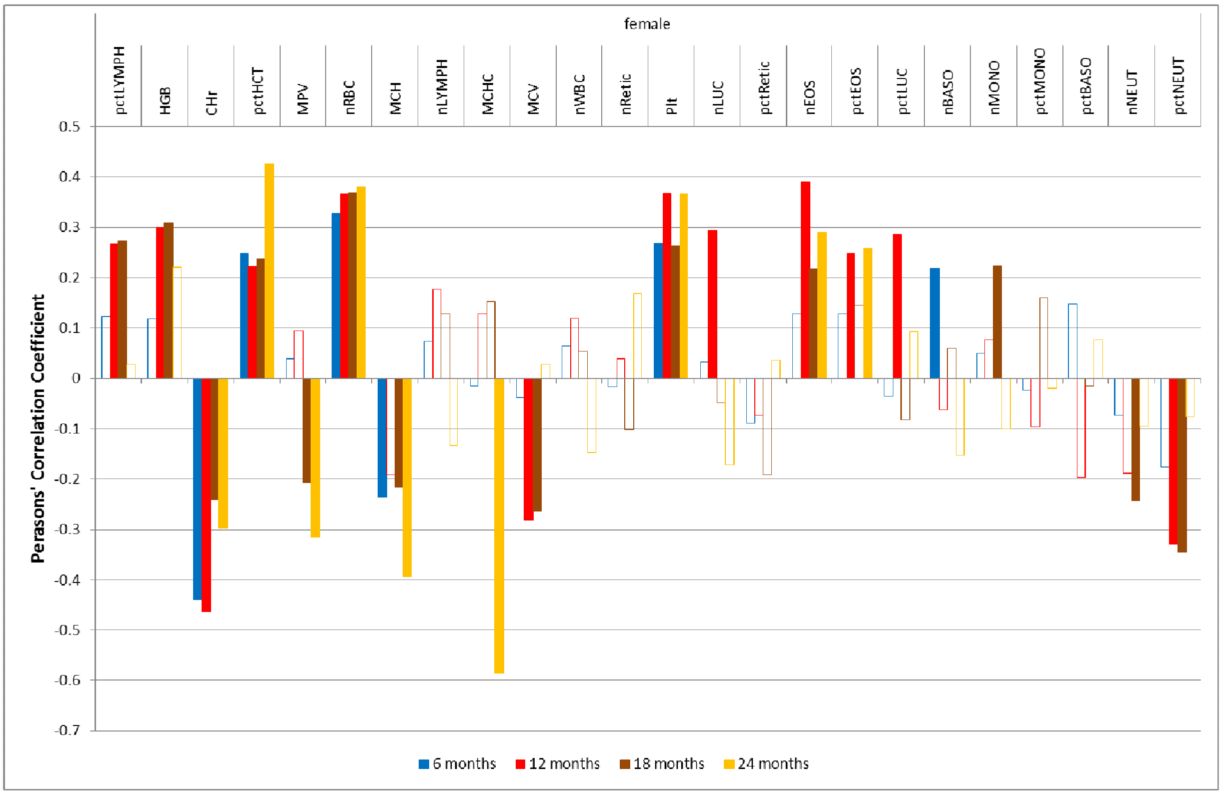

Suppl.II-Figure 4: Correlation Analysis on female blood cell count data (dataset: Peters4); irrelevant values (<|0.2| or / and p-value < 0.05) are presented by open bars

2.2.2 Correlation Analysis – Male – Peters4

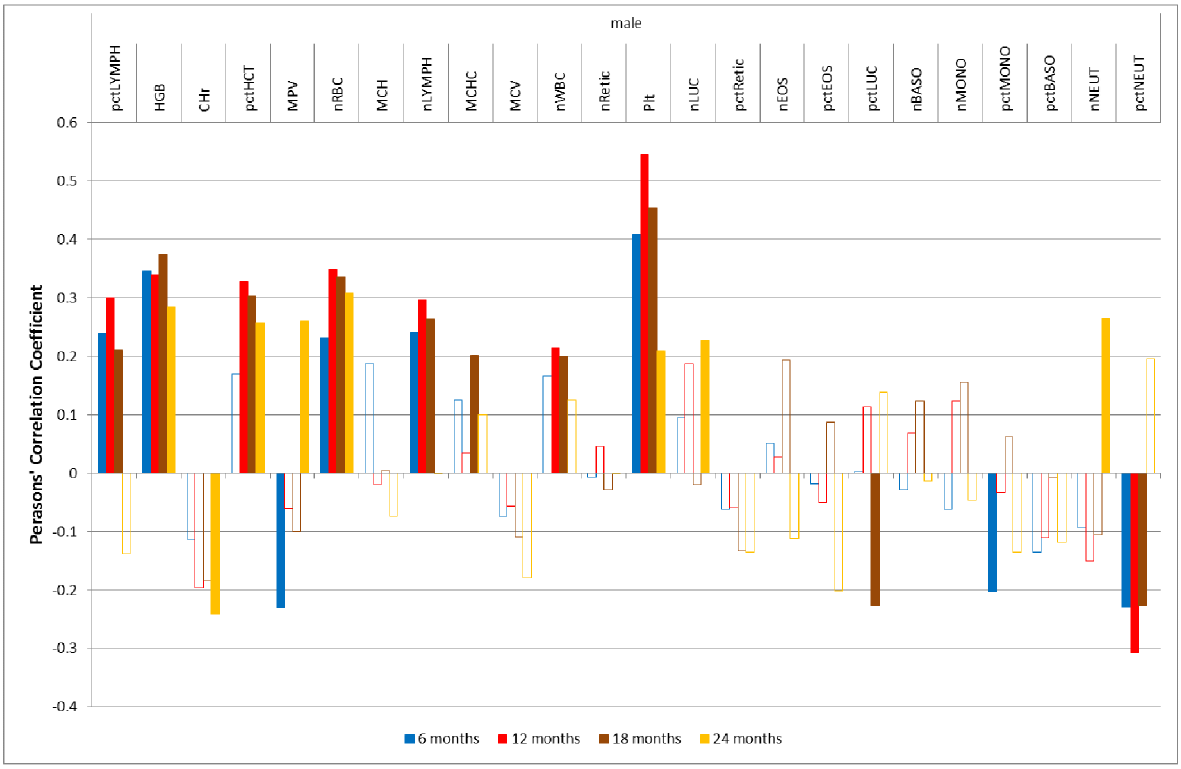

Suppl.II-Figure 5: Correlation Analysis on male blood cell count data (dataset: Peters4); irrelevant values (<|0.2| or / and p-value < 0.05) are presented by open bars.

3 Petkova1 - Leukocytes

3.1 Regression Analysis - Petkova1

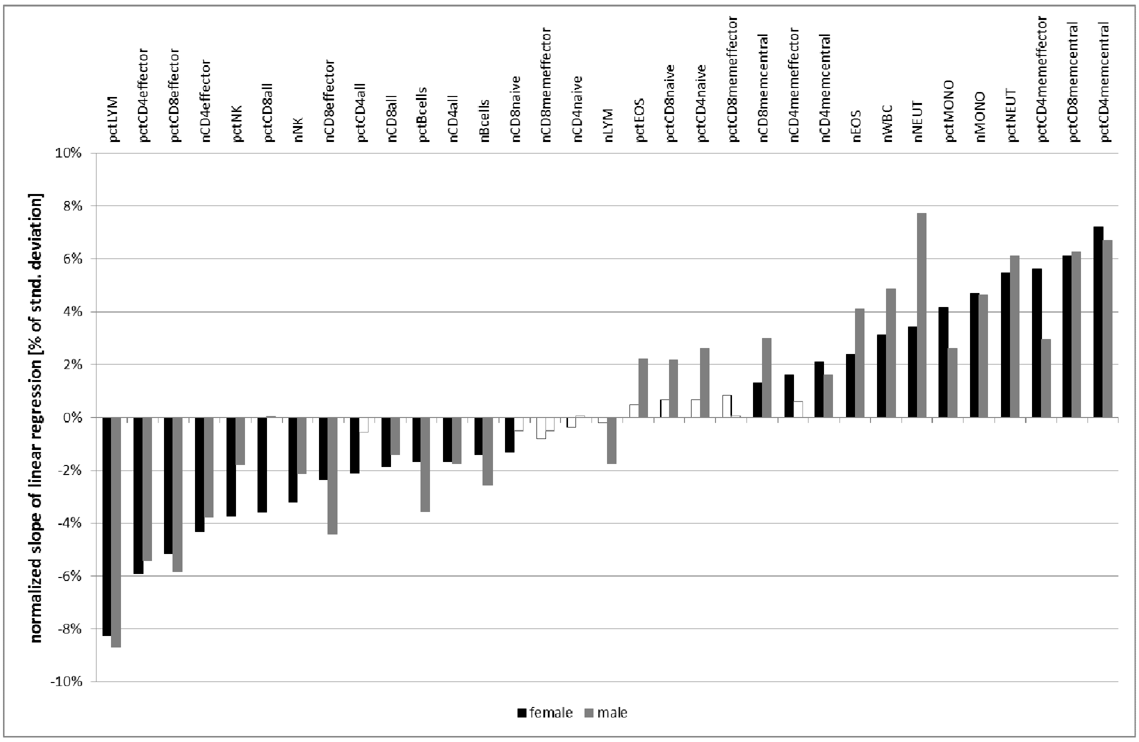

Suppl.II-Figure 6: Linear Regression on leukocyte data (dataset: Petkova1); statistically insignificant values are presented by open bars.

3.2 *Correlation Analysis - Petkova1*

3.2.1 Correlation Analysis – Female – Petkova1

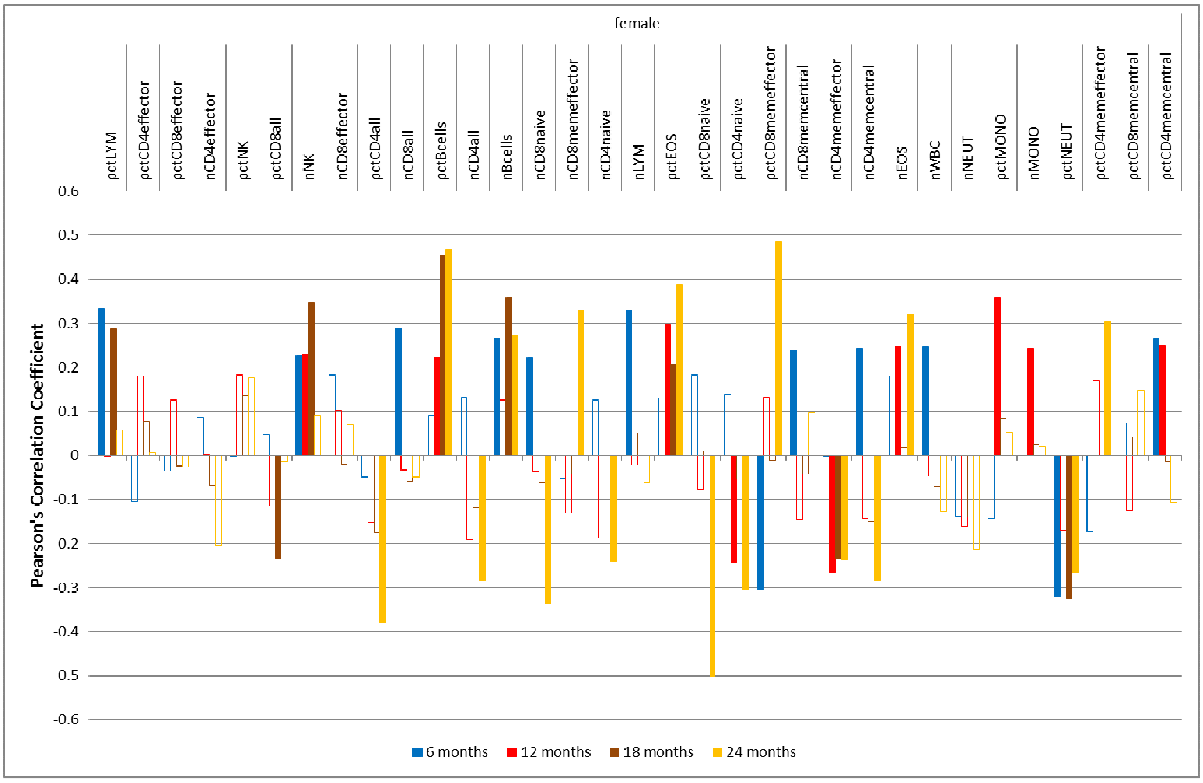

Suppl.II-Figure 7: Correlation Analysis on female leukocyte data (dataset: Petkova1); irrelevant values (<|0.2| or / and p-value < 0.05) are presented by open bars

3.2.2 Correlation Analysis – Male – Petkova1

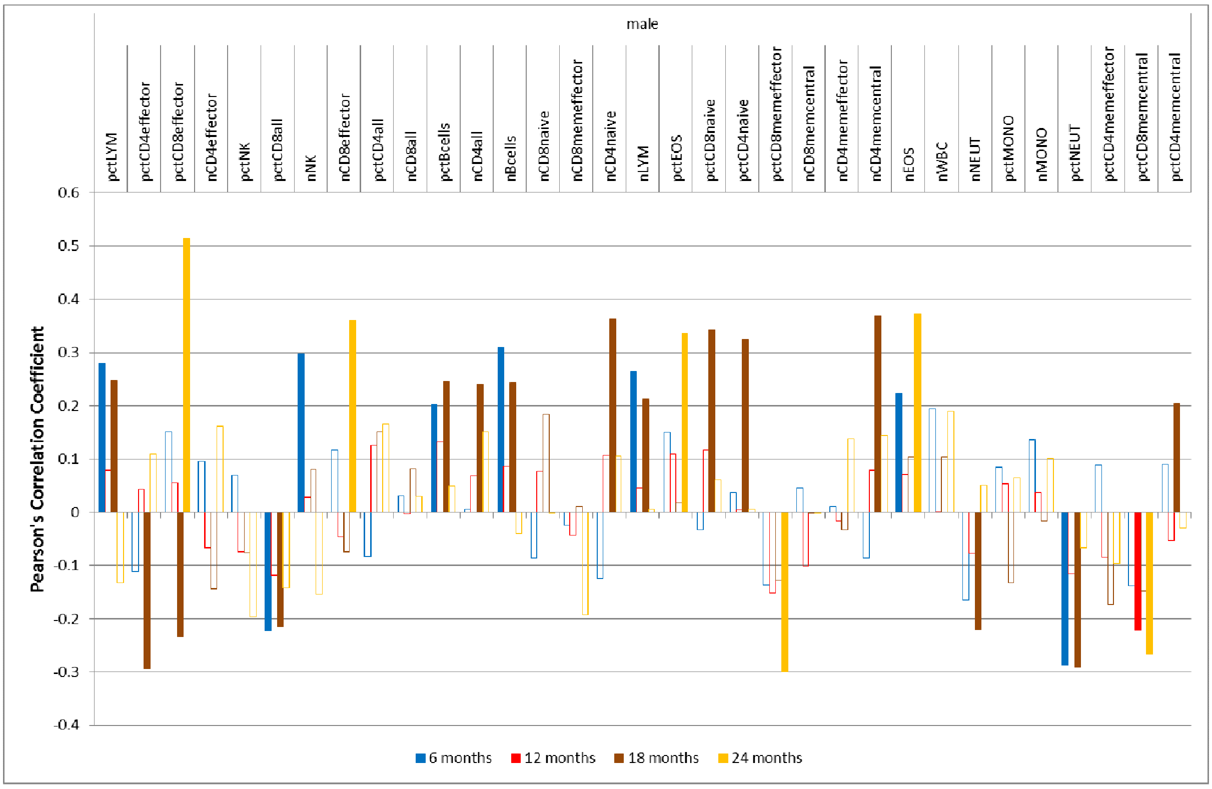

Suppl.II-Figure 8: Correlation Analysis on male leukocyte data (dataset: Petkova1); irrelevant values (<|0.2| or / and p-value < 0.05) are presented by open bars.

4 Yuan1 - IGF

4.1 Regression Analysis – Yuan1

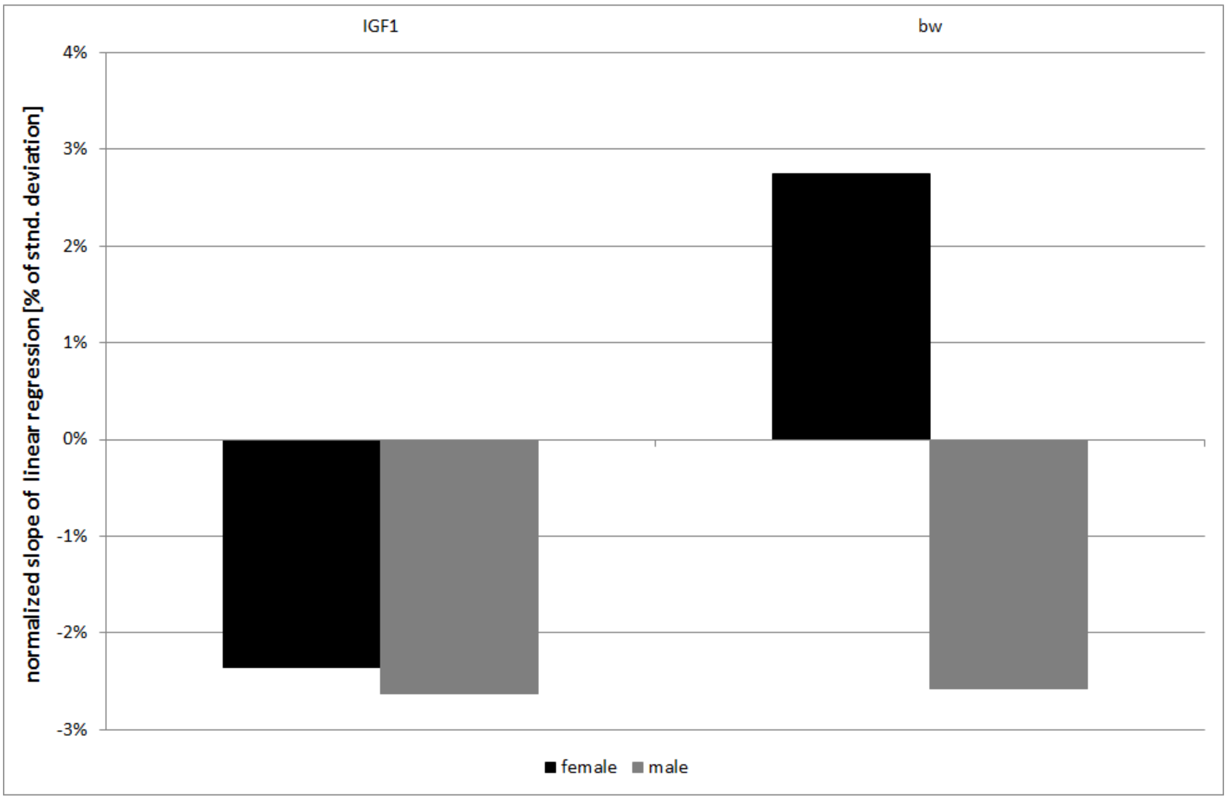

Suppl.II-Figure 9: Linear Regression on IGF-1 and bodyweight data (dataset: Yuan1); statistically insignificant values are presented by open bars.

4.2 Correlation Analysis – Yuan1

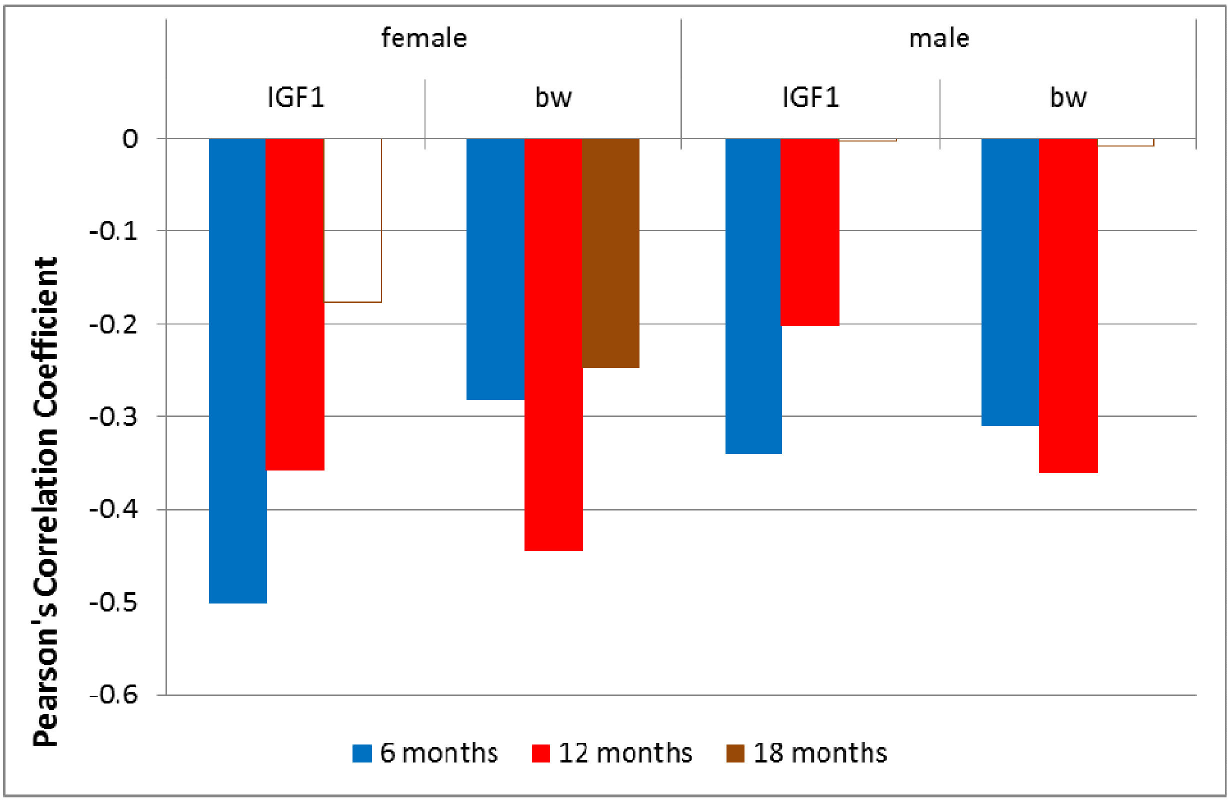

Suppl.II-Figure 10: Correlation Analysis on IGF-1 and bodyweight data (dataset: Yuan1); statistically insignificant values (<|0.2| or / and p-value < 0.05) are presented by open bars.

5 Yuan3 – Blood Chemistry

5.1 Regression Analysis – Yuan3

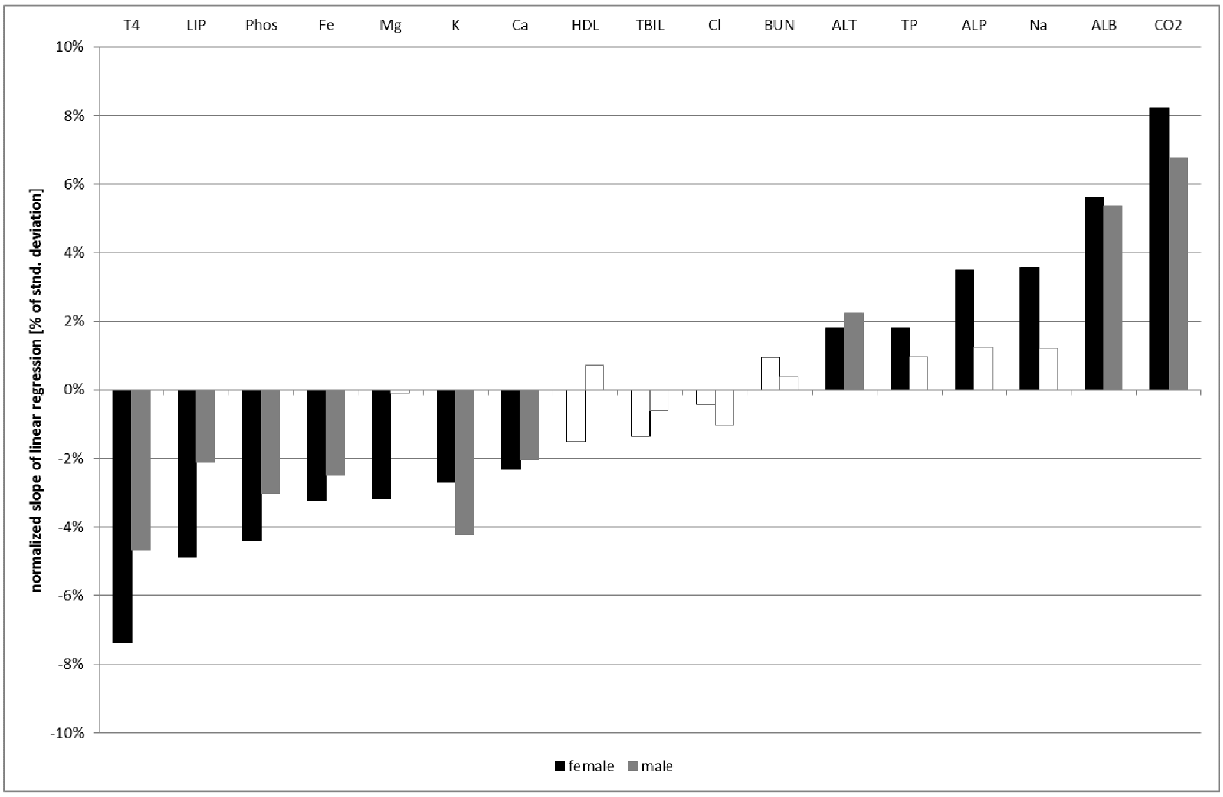

Suppl.II-Figure 11: Linear Regression on blood chemistry (dataset: Yuan3); statistically insignificant values are presented by open bars.

5.2 *Correlation Analysis – Yuan3*

5.2.1 Correlation Analysis – Female – Yuan3

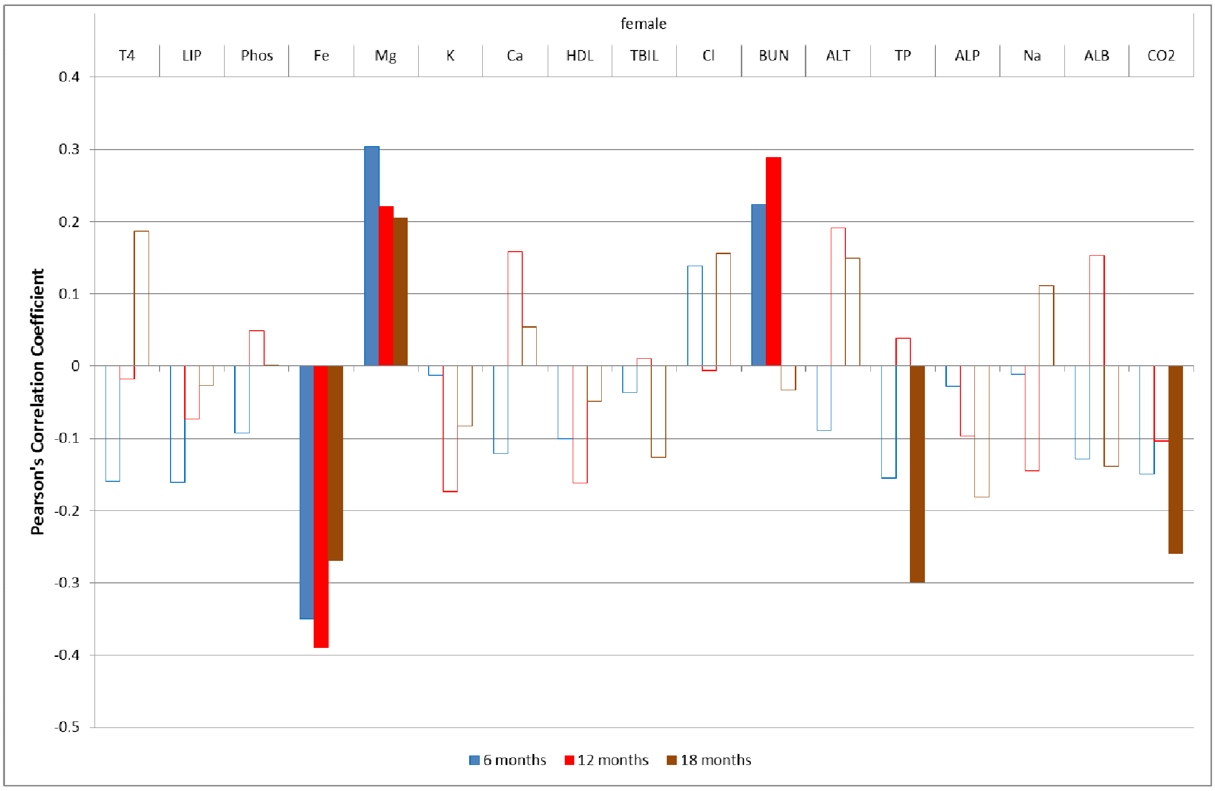

Suppl.II-Figure 12: Correlation Analysis on female blood chemistry (dataset: Yuan3); statistically insignificant values (<|0.2| or / and p-value < 0.05) are presented by open bars.

5.2.2 Correlation Analysis – Male – Yuan3

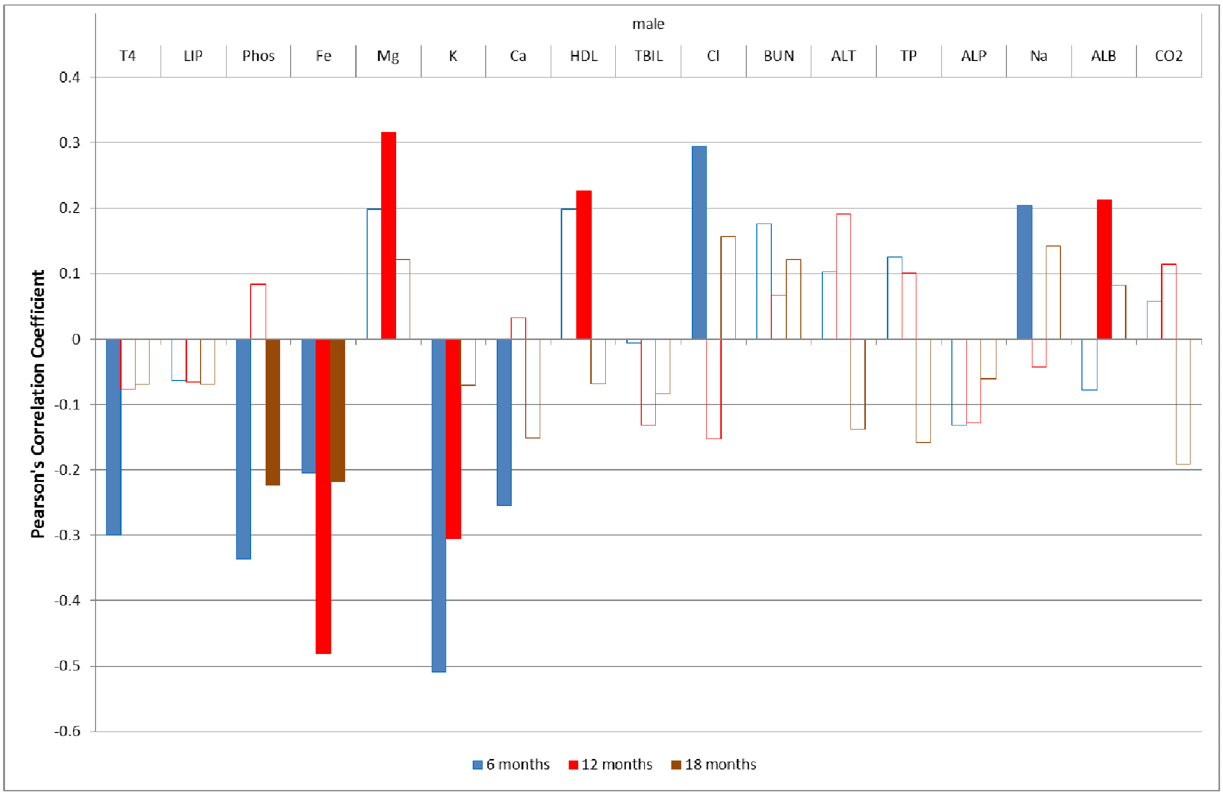

Suppl.II-Figure 13: Correlation Analysis on male blood chemistry (dataset: Yuan3); statistically insignificant values (<|0.2| or / and p-value < 0.05) are presented by open bars.
