# Supplementary material for: Inbred mouse strains reveal biomarkers that are pro-longevity, antilongevity or role switching
Source: Aging Cell. 2014 May 23;13(4):729–38. doi: 10.1111/acel.12226 (PMC4326954; doi:10.1111/acel.12226)
Supplement: Supplementary file 4 — Data S3 Comparison of longitudinal trends. [file acel0013-0729-sd4.pdf]

Supplement III

Comparison of slopes obtained from simple linear regression on short lived strains (life expectancy < 600 days) and long lived strains (life expectancy > 600 days)

1 Ackert1- Body Composition ____ 2

2 Peters4 – Blood Count ____ 3

3 Petkova1 - Leukocytes ____ 4

4 Yuan1 – IGF ____ 5

5 Yuan3 – Blood Chemistry ____ 6

1 Ackert1- Body Composition

strain	sex	BL	BMC	BMD	BMI	bone_area	BW	fatwt	LTM	pctfat	total_area	TTM
all of the below	female											
AKR/J	female											
MRL/MpJ	female											
PL/J	female											
SJL/J	female											
NZO/HILtJ	female											
all of the below	male											
AKR/J	male											
PL/J	male											
NZO/HILtJ	male											
BTBR T+ tf/J	male											
BUB/BnJ	male											
FVB/NJ	male											

faster trend
slower trend
opposite trend

2 Peters4 – Blood Count

strain	sex	BASO	CHr	EOS	HGB	LUC	LYMPH	MCH	MCHC	MCV	MONO	MPV	NEUT	pctBASO	pctEOS	pctHCT	pctLUC	pctLYMPH	pctMONO	pctNEUT	pctRetic	Plt	RBC	Retic	WBC
all of the below	female								faster		opposite														opposite
CAST/EiJ	female				slower				faster					slower	opposite	opposite	slower	slower		slower			opposite		
MRL/MpJ	female		faster			faster					opposite	faster			opposite		faster		opposite						
NZO/HILtJ	female		opposite	opposite								faster	opposite		opposite			slower		slower					
PL/J	female	opposite					faster		faster			opposite						faster	faster	faster		opposite			opposite
SJL/J	female						faster				opposite														opposite
all of the below	male		slower					opposite			slower												faster		
BTBR T+ tf/J	male					faster			opposite			faster							opposite						
BUB/BnJ	male																	opposite	slower	opposite		slower			
CAST/EiJ	male		opposite						opposite							opposite									
FVB/NJ	male							opposite		opposite	opposite	faster							opposite						
NZO/HILtJ	male								faster																
PL/J	male						faster											faster		faster					opposite
SJL/J	male	opposite		opposite		opposite	faster		faster			opposite													opposite

faster trend	(light green)
slower trend	(light yellow)
opposite trend	(blue)

3 Petkova1 - Leukocytes

strain	sex	nBcells	nCD4all	nCD4effector	nCD4memcentral	nCD4memeffector	nCD4naive	nCD8all	nCD8effector	nCD8memcentral	nCD8memeffector	nCD8naive	nEOS	nLYM	nMONO	nNEUT	nNK	nWBC	pctBcells	pctCD4all	pctCD4effector	pctCD4memcentral	pctCD4memeffector	pctCD4naive	pctCD8all	pctCD8effector	pctCD8memcentral	pctCD8memeffector	pctCD8naive	pctEOS	pctLYM	pctMONO	pctNEUT	pctNK
all of the below	female					faster										faster					faster	faster	faster											
CAST/EiJ	female																			faster	faster										opposite	opposite		slower
MRL/MpJ	female			slower								opposite				faster			faster		faster	faster		faster		faster			faster				faster	
NZO/HILtJ	female	opposite												opposite		faster		faster				slower	faster			opposite		faster	opposite					faster
PL/J	female		faster	faster			faster							faster					opposite	faster						opposite	opposite				faster		faster	opposite
SJL/J	female		faster	faster				faster	faster					faster	slower						faster	faster	faster			faster		faster						opposite
all of the below	male				faster	faster														opposite			faster			opposite	opposite							
AKR/J	male												faster	opposite				faster					faster						opposite					
BTBR T+ tf/J	male	opposite		faster	faster	faster	faster	opposite	faster	faster	opposite	opposite		opposite			opposite				faster	faster	faster	faster			slower	faster						
BUB/BnJ	male		opposite		faster	faster	faster					opposite			opposite				faster	opposite	faster			faster		faster		opposite	faster			opposite		
FVB/NJ	male		opposite			faster	faster		faster				faster	opposite			opposite		faster	opposite	faster	opposite		faster	opposite	faster				faster	opposite	opposite		
NZO/HILtJ	male	opposite					faster								faster				opposite						opposite									
PL/J	male		faster	faster	opposite		opposite	faster		opposite		faster		faster				opposite	opposite		opposite			opposite		opposite	opposite	faster	opposite		faster	faster	faster	slower
SJL/J	male			opposite						opposite					opposite		opposite		faster		opposite			opposite		opposite	opposite		opposite			opposite		

Legend	
faster trend	(light green)
slower trend	(light yellow)
opposite trend	(blue)

4 Yuan1 – IGF

strain	sex	bw	IGF1
all of the below	female		
CAST/EiJ	female		
MRL/MpJ	female		
NZO/HILtJ	female		
PL/J	female		
SJL/J	female		
all of the below	male		
BTBR T+ tf/J	male		
BUB/BnJ	male		
FVB/NJ	male		
NZO/HILtJ	male		
PL/J	male		
SJL/J	male		

faster trend
slower trend
opposite trend

5 Yuan3 – Blood Chemistry

strain	sex	ALB	ALP	ALT	BUN	Ca	Cl	CO2	Fe	HDL	K	LIP	Mg	Na	Phos	T4	TBIL	TP
all of the below	female																	
CAST/EiJ	female																	
MRL/MpJ	female																	
NZO/HILtJ	female																	
PL/J	female																	
SJL/J	female																	
all of the below	male																	
BTBR T+ tf/J	male																	
BUB/BnJ	male																	
FVB/NJ	male																	
NZO/HILtJ	male																	
PL/J	male																	
SJL/J	male																	

faster trend
slower trend
opposite trend
