# Supplementary material for: Inbred mouse strains reveal biomarkers that are pro-longevity, antilongevity or role switching
Source: Aging Cell. 2014 May 23;13(4):729–38. doi: 10.1111/acel.12226 (PMC4326954; doi:10.1111/acel.12226)
Supplement: Supplementary file 5 — Data S4 Supplementary figures: univariate Cox regression. [file acel0013-0729-sd5.pdf]

Supplementary Data IV

Supplementary Figures, Univariate Cox regression

1. Ackert1 - Bone mineral density and body composition 2
2. Korstanje1 - Urine albumin and creatinine 3
3. Mills1 - Chromosome instability and DNA Repair 4
4. Peters4 - Blood hematology in 30 inbred strains of mice 5
   - 4.1. Female mice 5
   - 4.2. Male mice 6
5. Petkova1 - Peripheral blood leukocytes (PBL profiles) in 32 inbred strains of mice 7
   - 5.1. Female mice 7
   - 5.2. Male mice 8
6. Xing1 - Electrocardiogram 9
7. Yuan1 - IGF-1 and bodyweight 10
8. Yuan3 - Blood chemistry for 32 inbred strains of mice 11
   - 8.1. Female mice 11
   - 8.2. Male mice 12

1. Ackert1 - Bone mineral density and body composition

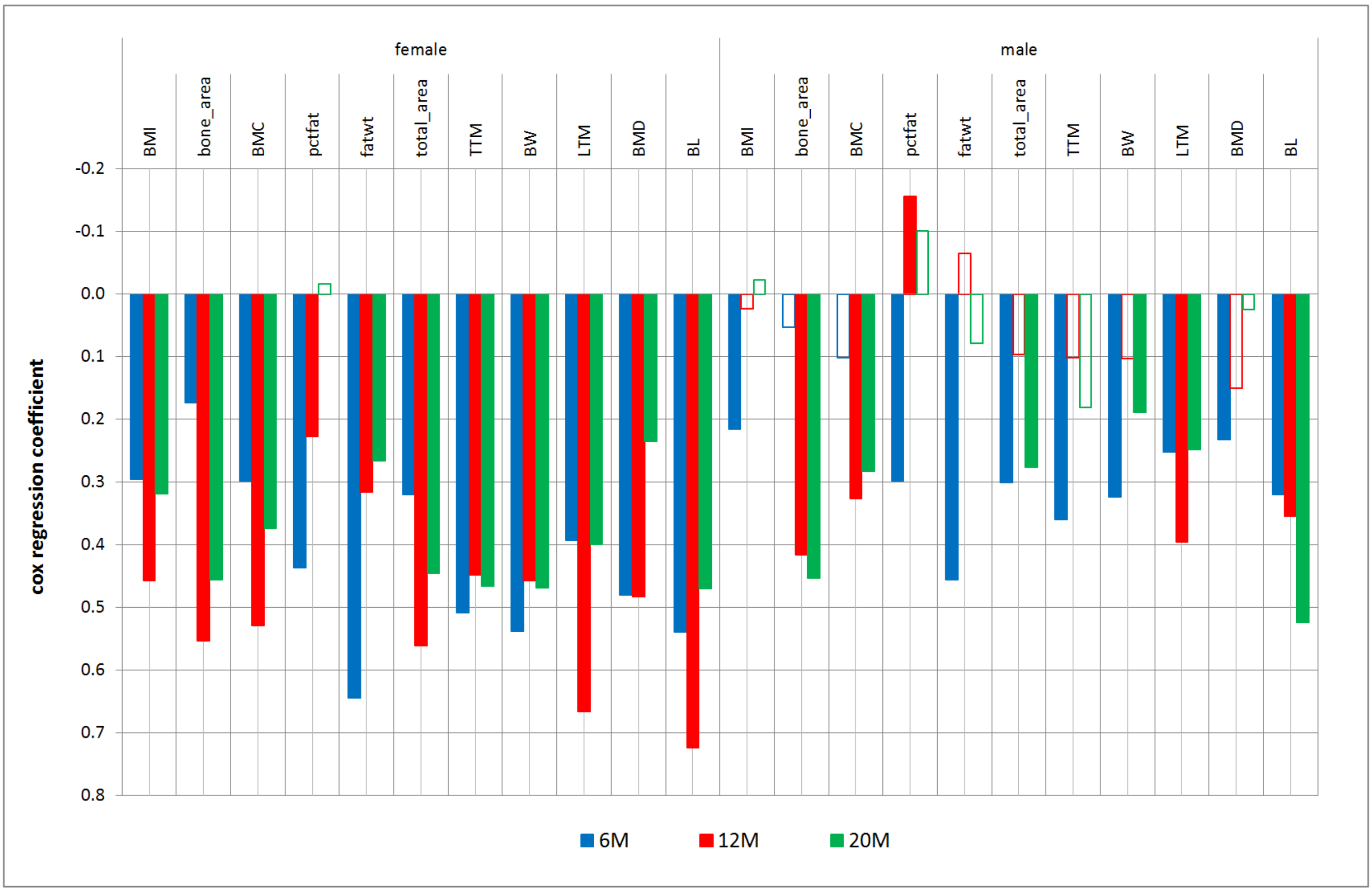

Supplementary Figure 1: Cox Regression on Bone mineral density and body composition (dataset: Ackert1) sorted from left to right by ascending female linear regression slopes; statistically insignificant values are presented by open bars. *Abbreviations of all features are indicated in Supplementary Table 2 of Supplement I.*

2. Korstanje1 - Urine albumin and creatinine

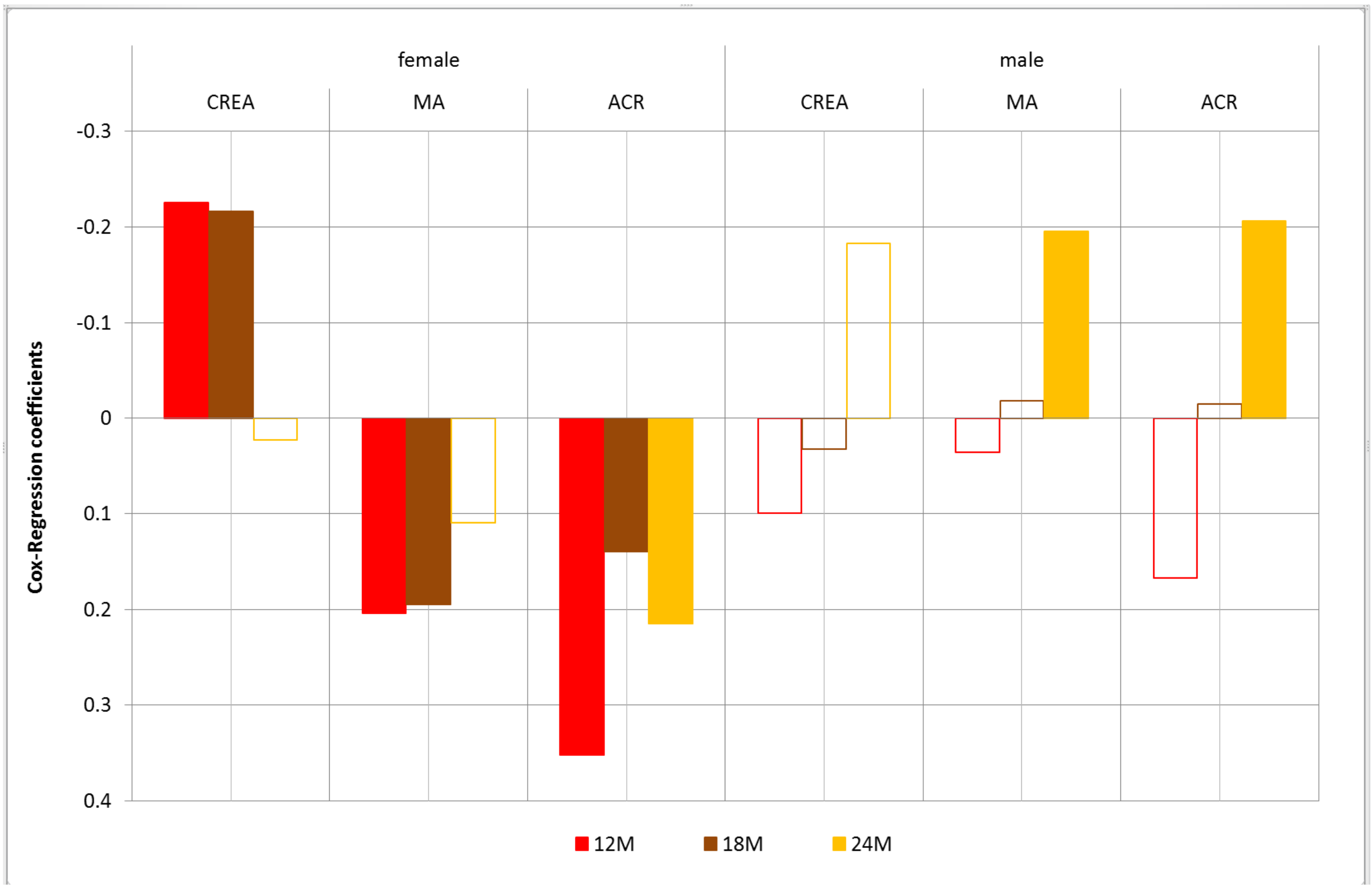

Supplementary Figure 2: Cox Regression on Urine Albumin and Creatinine (dataset: Korstanje1) sorted from left to right by ascending female linear regression slopes; statistically insignificant values are presented by open bars. Abbreviations of all features are indicated in Supplementary Table 2 of Supplement I.

3. Mills1 - Chromosome instability and DNA Repair

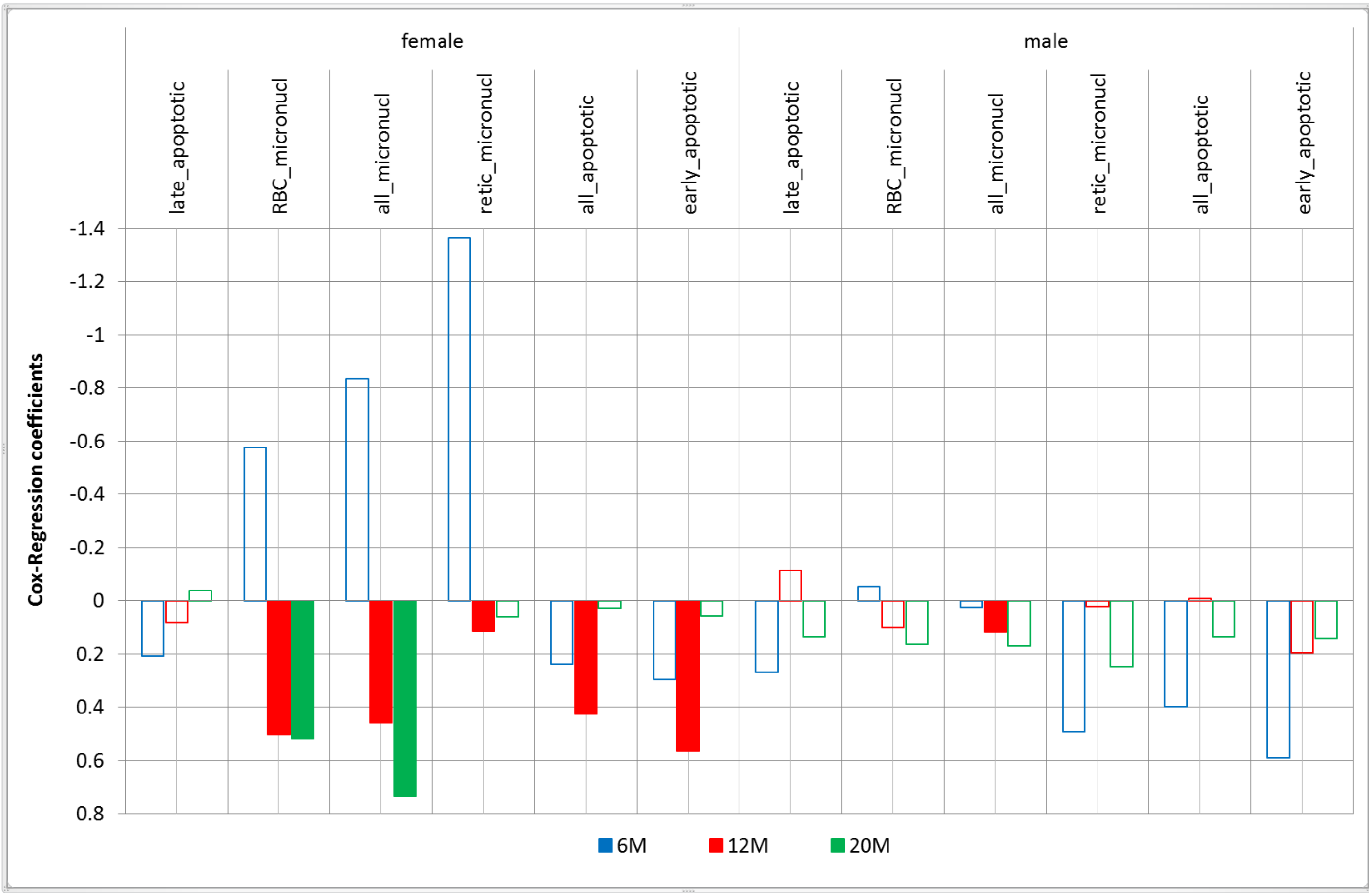

Supplementary Figure 3: Cox Regression on - Chromosome Instability and DNA Repair (dataset: Mills1) sorted from left to right by ascending female linear regression slopes; statistically insignificant values are presented by open bars. *Abbreviations of all features are indicated in Supplementary Table 2 of Supplement I.*

4. Peters4 - Blood hematology in 30 inbred strains of mice

4.1. Female mice

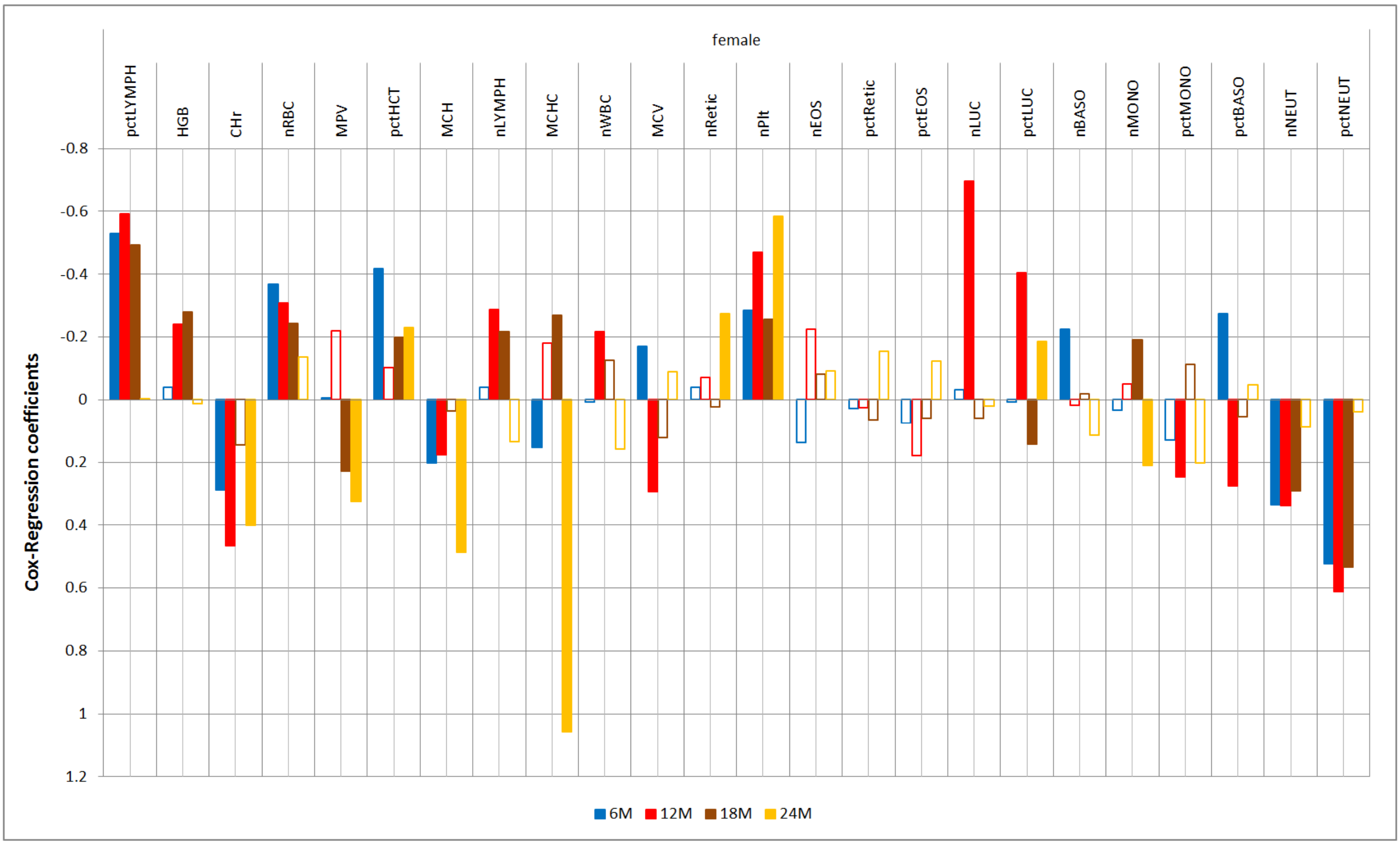

Supplementary Figure 4: Cox Regression on Blood cell count data (dataset: Peters4) sorted from left to right by ascending female linear regression slopes; statistically insignificant values are represented by open bars. *Abbreviations of all features are given in Supplementary Table 2 of Supplement I.*

4.2. Male mice

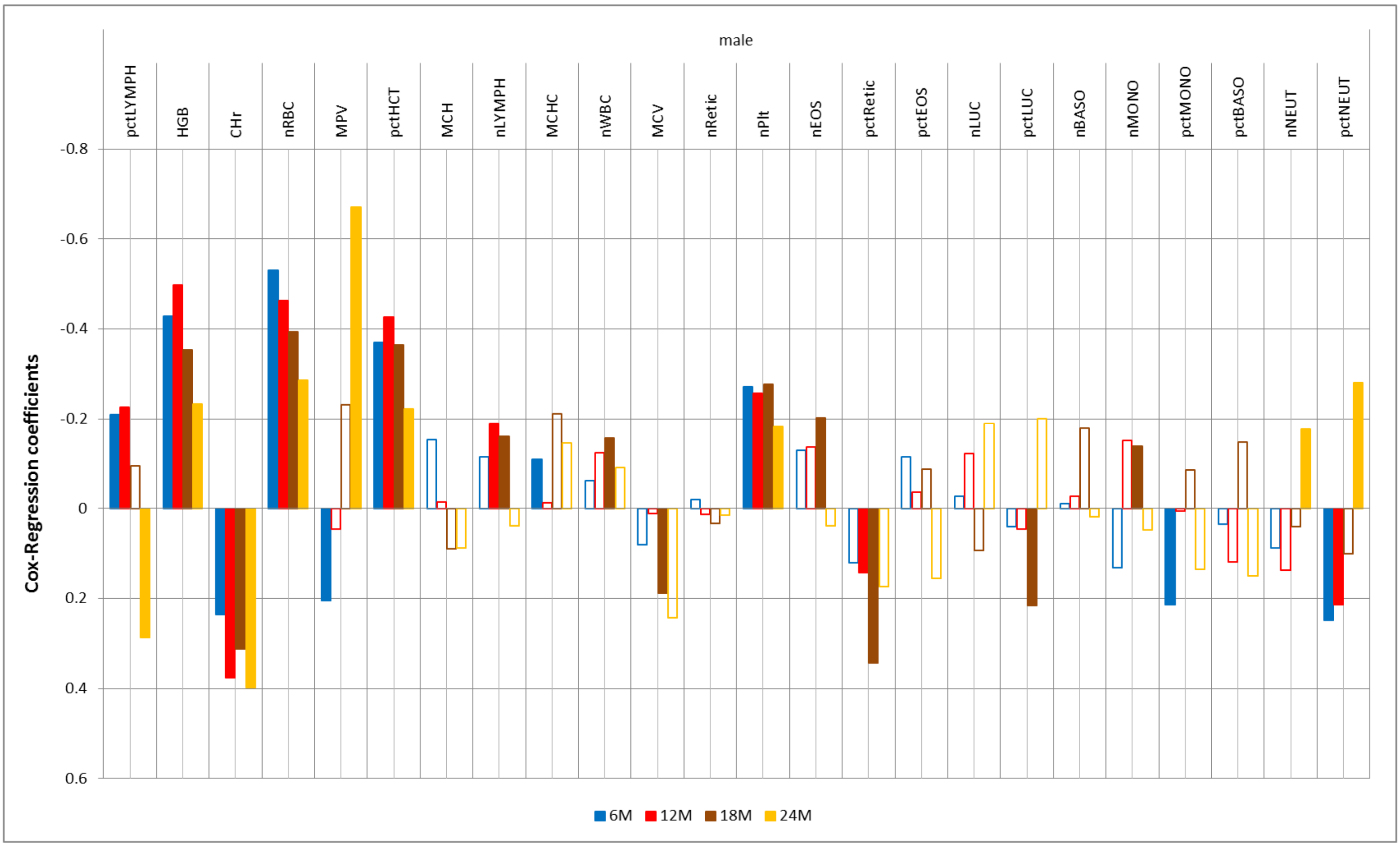

Supplementary Figure 5: Cox Regression on Blood cell count data (dataset: Peters4) sorted from left to right by ascending female linear regression slopes; statistically insignificant values are represented by open bars. *Abbreviations of all features are given in Supplementary Table 2 of Supplement I.*

5. Petkova1 - Peripheral blood leukocytes (PBL profiles) in 32 inbred strains of mice

5.1. Female mice

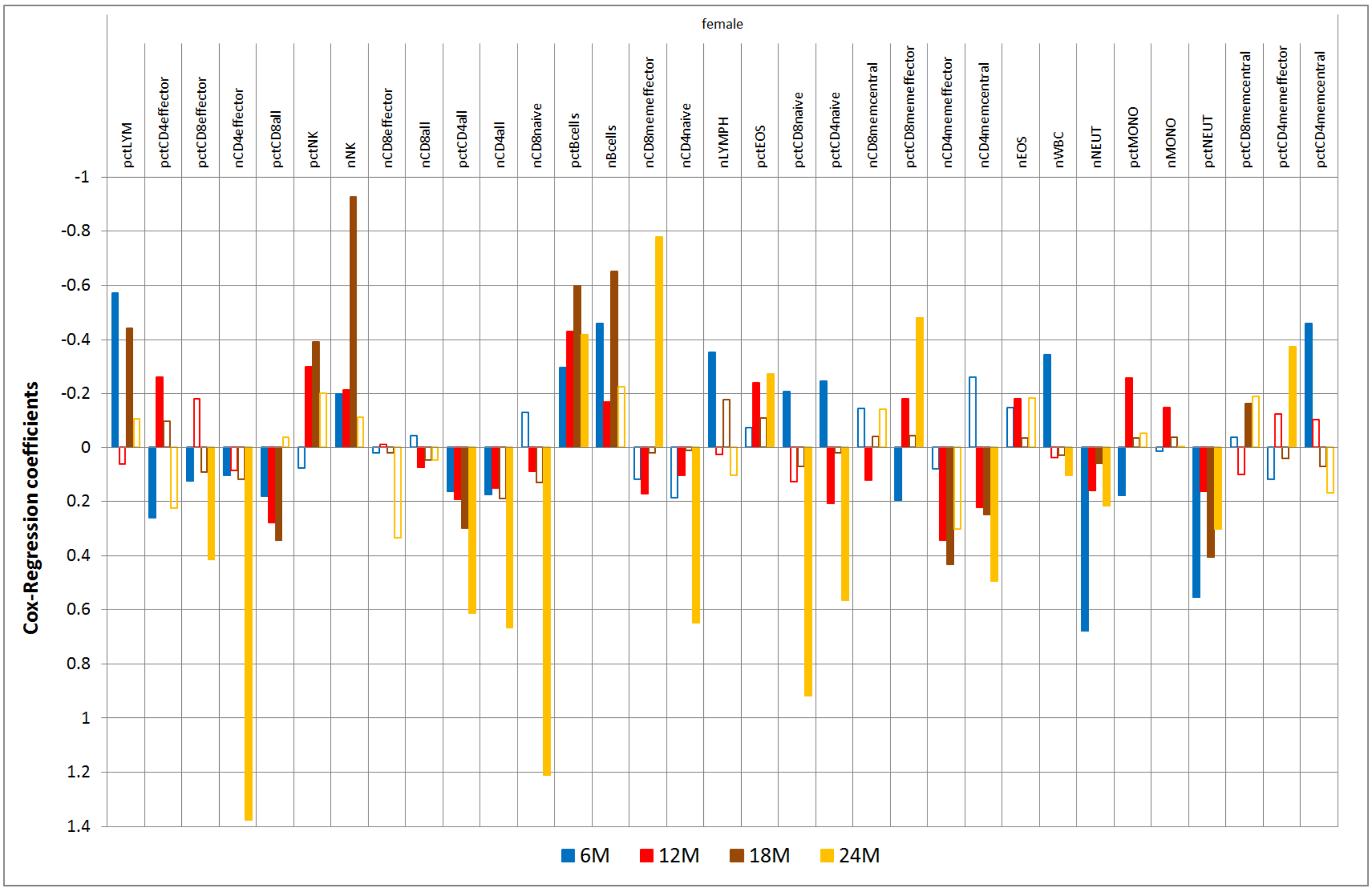

Supplementary Figure 6: Cox Regression on leukocyte data (dataset: Petkova1) sorted from left to right by ascending female linear regression slopes; statistically insignificant values are presented by open bars. *Abbreviations of all features are given in Supplementary Table 2 of Supplement I.*

5.2. Male mice

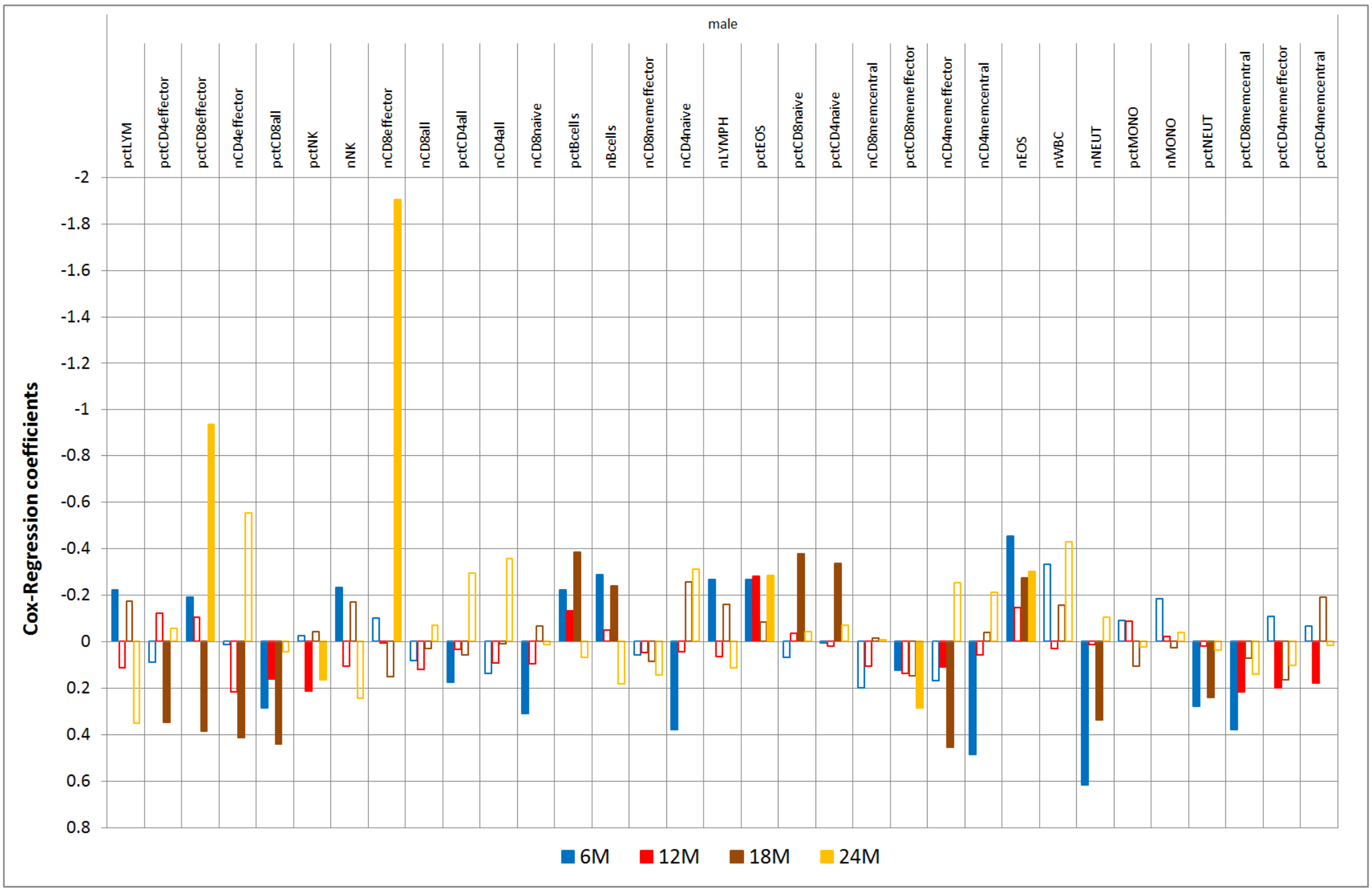

Supplementary Figure 7: Cox Regression on leukocyte data (dataset: Petkova1) sorted from left to right by ascending female linear regression slopes; statistically insignificant values are presented by open bars. *Abbreviations of all features are given in Supplementary Table 2 of Supplement I.*

6. Xing1 - Electrocardiogram

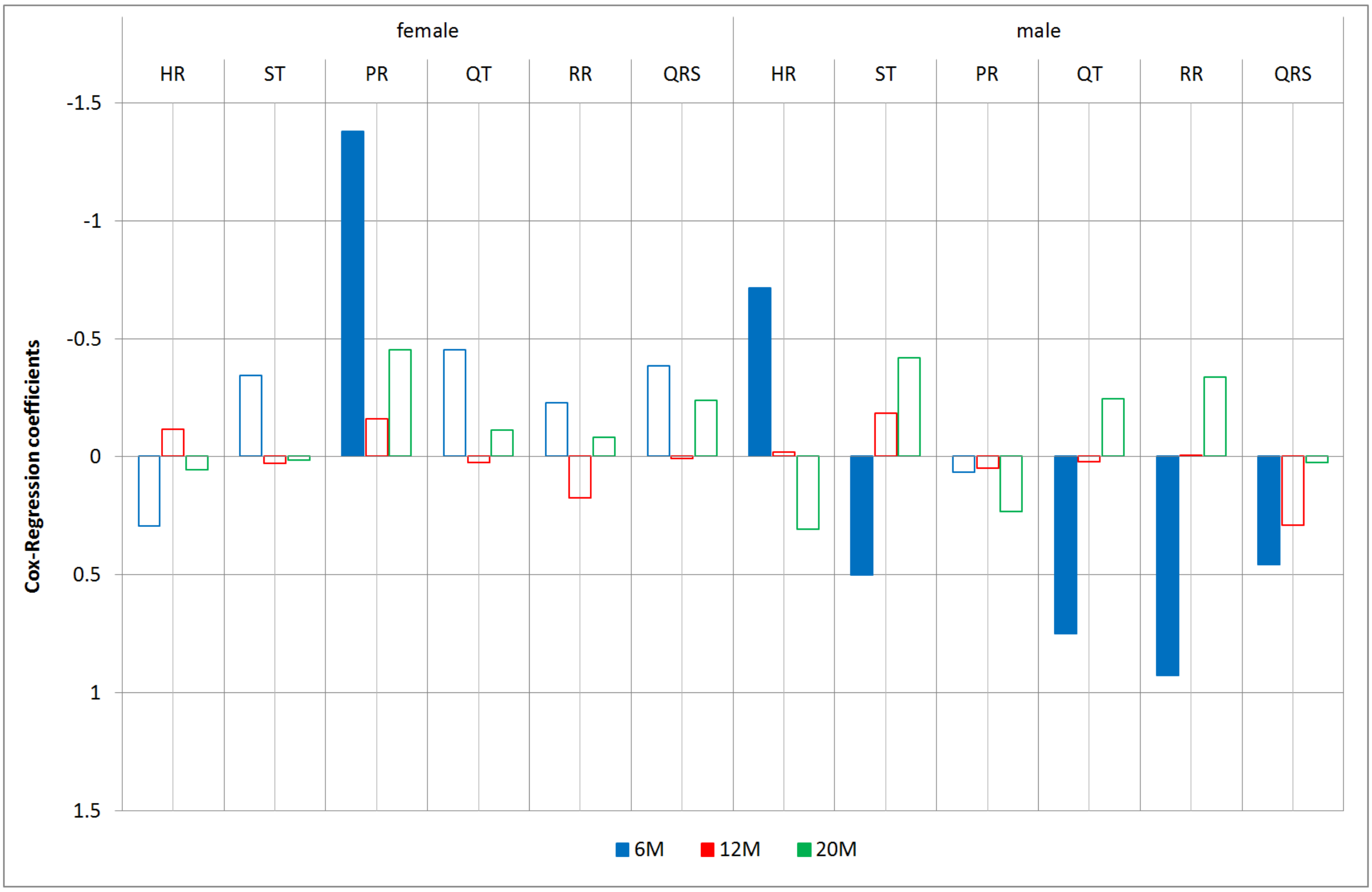

Supplementary Figure 8: Cox Regression on Electrocardiogram data (dataset: Xing1) sorted from left to right by ascending female linear regression slopes; statistically insignificant values are presented by open bars. Abbreviations of all features are indicated in *Supplementary Table 2 of Supplement I*.

7. Yuan1 - IGF-1 and bodyweight

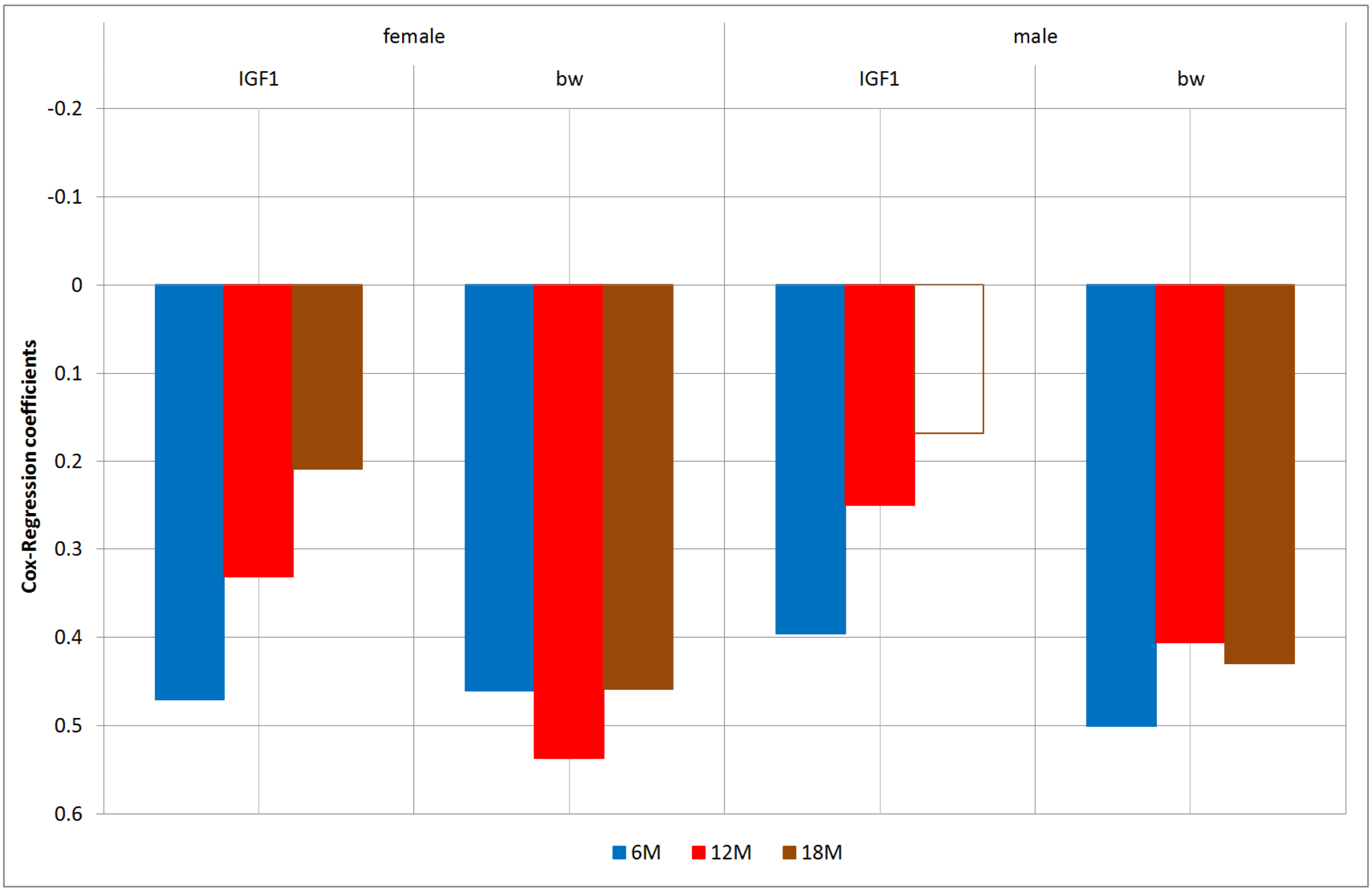

Supplementary Figure 9: Cox Regression on - IGF-1, bodyweight data (dataset: Yuan1) sorted from left to right by ascending female linear regression slopes; statistically insignificant values are presented by open bars. *Abbreviations of all features are indicated in Supplementary Table 2 of Supplement I.*

8. Yuan3 - Blood chemistry for 32 inbred strains of mice

8.1. Female mice

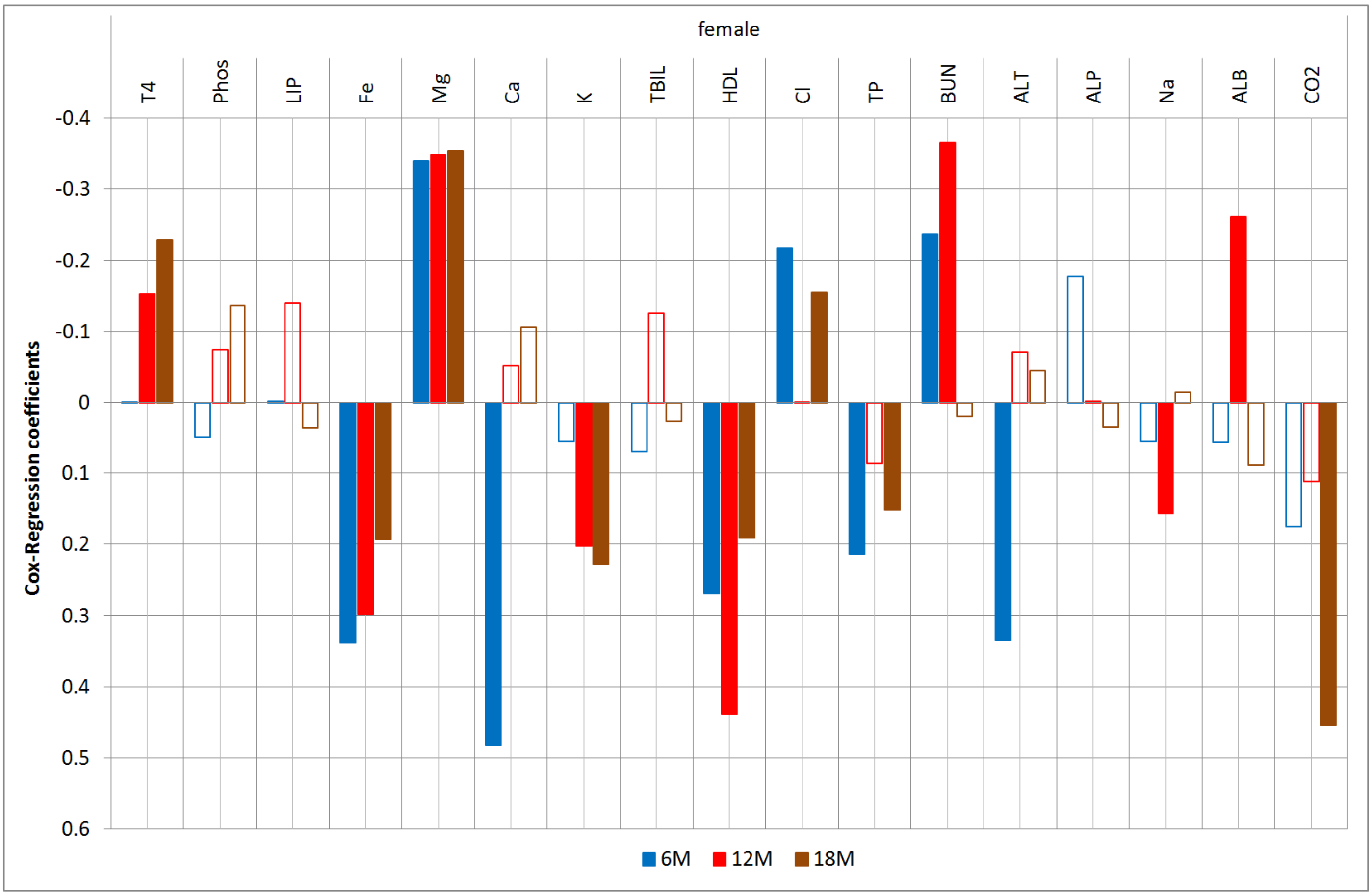

Supplementary Figure 10: Cox Regression on Blood Chemistry (dataset: Yuan3) sorted from left to right by ascending female linear regression slopes; statistically insignificant values are presented by open bars. *Abbreviations of all features are indicated in Supplementary Table 2 of Supplement I.*

8.2. Male mice

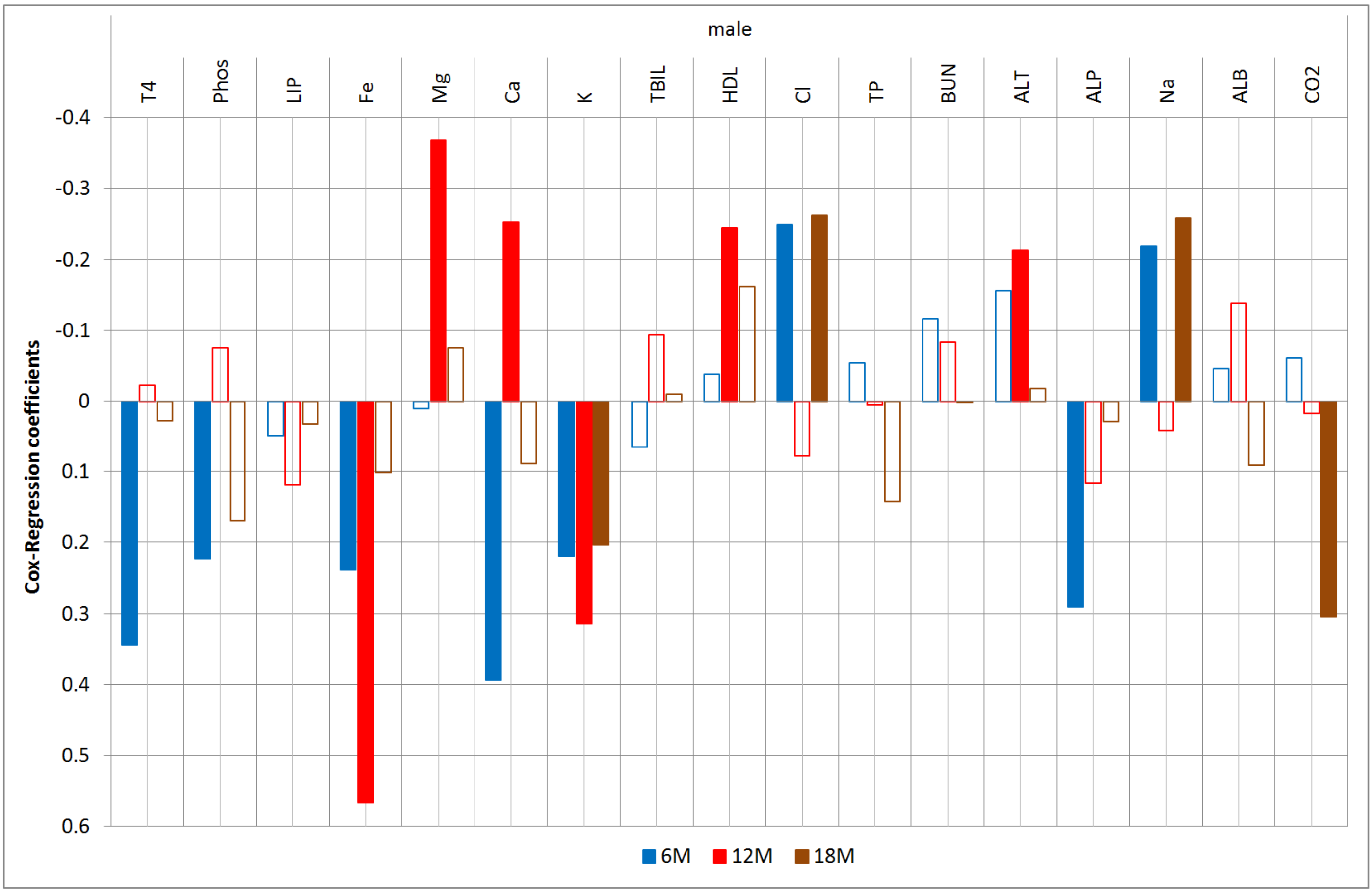

Supplementary Figure 11: Cox Regression on Blood Chemistry (dataset: Yuan3) sorted from left to right by ascending female linear regression slopes; statistically insignificant values are presented by open bars. *Abbreviations of all features are indicated in Supplementary Table 2 of Supplement I.*
