# Supplementary material for: Inbred mouse strains reveal biomarkers that are pro-longevity, antilongevity or role switching
Source: Aging Cell. 2014 May 23;13(4):729–38. doi: 10.1111/acel.12226 (PMC4326954; doi:10.1111/acel.12226)
Supplement: Supplementary file 6 — Data S5 Supplementary tables and figures: multivariate Cox regression. [file acel0013-0729-sd6.pdf]

Supplementary Data V

Supplementary Tables and Figures: Multivariate Cox regression.

1. Data set used for multivariate Cox regression 2

2. Redundancy / collinearity 3

3. Cox models 4

3.1 Female Mice 4

3.1.1 Age: 6M 4

3.1.2 Age: 12M 5

3.1.3 Age: 18M 6

3.1.4 Age: 24M 7

3.2 Male Mice 8

3.2.1 Age: 6M 8

3.2.2 Age: 12M 9

3.2.3 Age: 18M 10

3.2.4 Age: 24M 11

1. Data set used for multivariate Cox regression

For a multivariate Cox regression, we used the features in Table 1 of the main article, reproduced in Supplementary Table 1. Heart rate was excluded because it was only measured in 6 strains.

Feature	Abbreviation	Data Set
Body-Mass-Index	BMI	Ackert1
red blood cells	RBC	Peters4
hemoglobin	HGB	Peters4
hematocrit	pctHCT	Peters4
ret. corp. hemoglobin	CHr	Peters4
B-cells	nBCells	Petkova1
lymphocytes	nLYMPH	Petkova1
neutrophils	nNEUT	Petkova1
iron	Fe	Yuan3
thyroxine	T4	Yuan3
magnesium	Mg	Yuan3

Supplementary Table 1: Selected Features

No individual mice were used in more than one dataset. Data of all mice of a strain were therefore aggregated, so that the number of "individuals" in the multivariate Cox regression analysis is the number of strains. Cox regression using the 'survival' package in R allows no missing values. We thus assigned 20 months measurements in the data sets Ackert1 and Xing1 and 18 month values in the other data sets to the same age group. For the 24 months age group there were no measurements of BMI, Fe, Mg and T4, reducing the number of features from 11 to 7. Supplementary Table 2 lists the number of strains per age group and sex.

sex	6 months	12 months	18/20 months	24months
Female	27	24	21	11
Male	23	23	19	10

Supplementary Table 2: Remaining strains by age group and sex

2. Redundancy / collinearity

To quantify collinearity (i.e. in how far a linear relationship exists between two or more explanatory variables, rendering some of them redundant in case of perfect linear relationships), we used the variance inflation factor (VIF) as implemented in the R package “HH” (Statistical Analysis and Data Display by Heiberger and Holland), see Supplementary Table 3.

		explanatory variables										
sex	age	BMI	CHr	Fe	HGB	Mg	nBcells	nLYMPH	nNEUT	pctHCT	RBC	T4
Female	6M	2.0	2.2	2.1	1.9	1.6	2.1	1.8	1.7	2.4	3.2	1.4
	12M	3.6	5.1	1.5	9.2	1.8	4.5	4.3	2.6	6.1	10.8	2.0
	18M	1.8	7.8	4.0	26.8	2.2	3.7	2.8	2.3	19.1	13.6	1.8
	24M	N/A	23.8	N/A	150.8	N/A	7.8	4.0	3.3	51.2	112.4	N/A
Male	6M	2.3	2.4	1.9	7.0	2.0	4.2	3.4	3.1	9.1	4.0	1.3
	12M	2.9	9.1	3.9	13.7	2.4	8.5	8.7	3.2	6.8	17.0	1.6
	18M	7.9	9.7	3.3	8.5	1.4	5.8	5.1	2.0	28.2	21.7	5.9
	24M	N/A	4.9	N/A	30.7	N/A	4.2	4.8	3.8	48.5	113.3	N/A

Supplementary Table 3: Variance Inflation Faktors of explanatory variables. Collinearity that exceeds a threshold of 10.0 is marked in red. N/A: no data available.

The features Chr, HGB, RBC and pctHCT show the highest collinearity, reflecting their close association with erythrocytes.

3. Cox models

3.1 Female Mice

3.1.1 Age: 6M

R formula to calculate Cox model

coxph(formula = my.surv.object ~ BMI + CHr + Fe + HGB + Mg + nBcells + nLYMPH + nNEUT + pctHCT + RBC + T4, data)

Cox model

Feature	coef	exp(coef)	se(coef)	z	Pr(>|z|)	Signif.
BMI	0.6938	2.0014	0.4985	1.392	0.164	
CHr	0.1025	1.108	0.548	0.187	0.8516	
Fe	0.1922	1.2119	0.5031	0.382	0.7025	
HGB	-0.3236	0.7236	0.5584	-0.579	0.5623	
Mg	-0.1202	0.8867	0.3325	-0.361	0.7177	
nBcells	-1.2814	0.2777	0.5886	-2.177	0.0295	*
nLYMPH	0.869	2.3845	0.5254	1.654	0.0981	.
nNEUT	1.2907	3.6353	0.9828	1.313	0.1891	
pctHCT	-0.7445	0.475	0.6677	-1.115	0.2648	
RBC	0.646	1.9079	0.5207	1.241	0.2147	
T4	0.3431	1.4093	0.3024	1.135	0.2564	

Signif. codes: 0 ‘***’ 0.001 ‘**’ 0.01 ‘*’ 0.05 ‘.’ 0.1 ‘ ’ 1

Model quality Scores
Concordance = 0.729 (se = 0.068)
Rsquare = 0.502 (max possible= 0.992)
Likelihood ratio test = 18.81 on 11 df, p=0.06462
Wald test = 14.57 on 11 df, p=0.203
Score (logrank) test = 18.15 on 11 df, p=0.07811

3.1.2 Age: 12M

R formula to calculate Cox model

coxph(formula = my.surv.object ~ BMI + CHr + Fe + HGB + Mg + nBcells + nLYMPH + nNEUT + pctHCT + RBC + T4, data)

Cox model

Feature	coef	exp(coef)	se(coef)	z	Pr(>|z|)	Signif.
BMI	3.49019	32.79207	1.378277	2.532	0.011332	*
CHr	3.78147	43.88035	1.115887	3.389	0.000702	***
Fe	1.21741	3.378414	0.412895	2.948	0.003194	**
HGB	-5.2664	0.005162	2.171997	-2.425	0.015322	*
Mg	-1.7403	0.175468	0.822725	-2.115	0.034405	*
nBcells	0.53268	1.703491	0.592715	0.899	0.368806	
nLYMPH	-0.2591	0.771724	0.488387	-0.531	0.59571	
nNEUT	0.22235	1.249011	0.871836	0.255	0.798693	
pctHCT	1.85982	6.422597	1.718238	1.082	0.279074	
RBC	4.69251	109.127	1.552385	3.023	0.002505	**
T4	-1.2892	0.275495	0.538141	-2.396	0.016592	*

Signif. codes: 0 '***' 0.001 '**' 0.01 '*' 0.05 '.' 0.1 ' ' 1

Model quality Scores
Concordance = 0.888 (se = 0.073)
Rsquare = 0.835 (max possible= 0.99)
Likelihood ratio test = 43.26 on 11 df, p=9.775e-06
Wald test = 16.76 on 11 df, p=0.1151
Score (logrank) test = 31.2 on 11 df, p=0.001023

3.1.3 Age: 18M

R formula to calculate Cox model

coxph(formula = my.surv.object ~ BMI + CHr + Fe + HGB + Mg + nBcells + nLYMPH + nNEUT + pctHCT + RBC + T4, data)

Cox model

Feature	coef	exp(coef)	se(coef)	z	Pr(>|z|)	Signif.
BMI	0.0552	1.0568	0.7175	0.077	0.9387	
CHr	1.4367	4.2069	0.7751	1.854	0.0638	.
Fe	-0.2367	0.7893	0.375	-0.631	0.528	
HGB	-3.4359	0.0322	1.7949	-1.914	0.0556	.
Mg	0.4506	1.5692	0.3898	1.156	0.2477	
nBcells	-1.6638	0.1894	0.7197	-2.312	0.0208	*
nLYMPH	0.2598	1.2967	0.3427	0.758	0.4483	
nNEUT	0.3048	1.3564	0.2569	1.187	0.2354	
pctHCT	0.6344	1.8858	1.1995	0.529	0.5969	
RBC	2.5494	12.799	1.1272	2.262	0.0237	*
T4	-0.7734	0.4614	0.5105	-1.515	0.1298	

Signif. codes: 0 ‘***’ 0.001 ‘**’ 0.01 ‘*’ 0.05 ‘.’ 0.1 ‘ ’ 1

Model quality Scores
Concordance = 0.833 (se = 0.079)
Rsquare = 0.631 (max possible= 0.987)
Likelihood ratio test = 20.95 on 11 df, p=0.03386
Wald test = 13.11 on 11 df, p=0.2861
Score (logrank) test = 17.89 on 11 df, p=0.0842

3.1.4 Age: 24M

R formula to calculate Cox model

coxph(formula = my.surv.object ~ CHr + HGB + nBcells + nLYMPH + nNEUT + pctHCT + RBC, data)

Cox model

Feature	coef	exp(coef)	se(coef)	z	Pr(>|z|)	Signif.
CHr	-3.7	0.0	3.3	-1.133	0.257	
HGB	16.5	13870000.0	10.4	1.587	0.112	
nBcells	5.6	269.1	3.9	1.421	0.155	
nLYMPH	-0.4	0.7	1.3	-0.3	0.765	
nNEUT	-2.6	0.1	2.4	-1.077	0.282	
pctHCT	-14.7	0.0	9.1	-1.613	0.107	
RBC	-5.6	0.0	4.5	-1.232	0.218	

Signif. codes: 0 ‘***’ 0.001 ‘**’ 0.01 ‘*’ 0.05 ‘.’ 0.1 ‘ ’ 1

Model quality Scores
Concordance = 0.873 (se = 0.117)
Rsquare = 0.774 (max possible= 0.959)
Likelihood ratio test = 16.38 on 7 df, p=0.02185
Wald test = 6.57 on 7 df, p=0.4746
Score (logrank) test = 19.34 on 7 df, p=0.007188

3.2 Male Mice

3.2.1 Age: 6M

R formula to calculate Cox model

coxph(formula = my.surv.object ~ BMI + CHr + Fe + HGB + Mg + nBcells + nLYMPH + nNEUT + pctHCT + RBC + T4, data)

Cox model

Feature	coef	exp(coef)	se(coef)	z	Pr(>|z|)	Signif.
BMI	0.8524	2.3452	0.6802	1.253	0.2101	
CHr	-0.4096	0.6639	0.6101	-0.671	0.502	
Fe	0.368	1.4449	0.4767	0.772	0.4401	
HGB	0.303	1.3539	0.8077	0.375	0.7076	
Mg	0.57	1.7683	0.4146	1.375	0.1692	
nBcells	-1.2563	0.2847	0.7263	-1.73	0.0837	.
nLYMPH	1.749	5.7488	0.7716	2.267	0.0234	*
nNEUT	0.6383	1.8932	1.2675	0.504	0.6146	
pctHCT	-0.8297	0.4362	0.8563	-0.969	0.3326	
RBC	-0.5609	0.5707	0.698	-0.804	0.4216	
T4	0.7345	2.0845	0.3145	2.336	0.0195	*

Signif. codes: 0 ‘***’ 0.001 ‘**’ 0.01 ‘*’ 0.05 ‘.’ 0.1 ‘ ’ 1

Model quality Scores
Concordance = 0.747 (se = 0.075)
Rsquare = 0.565   (max possible= 0.989)
Likelihood ratio test = 19.15 on 11 df,   p=0.05848
Wald test = 16.23 on 11 df,   p=0.1328
Score (logrank) test = 20.55 on 11 df,   p=0.03833

3.2.2 Age: 12M

R formula to calculate Cox model

coxph(formula = my.surv.object ~ BMI + CHr + Fe + HGB + Mg + nBcells + nLYMPH + nNEUT + pctHCT + RBC + T4, data)

Cox model

Feature	coef	exp(coef)	se(coef)	z	Pr(>|z|)	Signif.
BMI	0.13282	1.14205	0.76651	0.173	0.8624	
CHr	0.90934	2.48268	0.8953	1.016	0.3098	
Fe	1.99205	7.33054	0.83178	2.395	0.0166	*
HGB	-1.57378	0.20726	1.11821	-1.407	0.1593	
Mg	-0.77992	0.45844	0.39889	-1.955	0.0506	.
nBcells	-0.07585	0.92695	0.81656	-0.093	0.926	
nLYMPH	0.65166	1.91872	1.09983	0.593	0.5535	
nNEUT	-1.08369	0.33835	0.74043	-1.464	0.1433	
pctHCT	-0.9417	0.38997	0.61783	-1.524	0.1275	
RBC	2.50466	12.23942	1.32616	1.889	0.0589	.
T4	-0.04378	0.95717	0.40069	-0.109	0.913	

Signif. codes: 0 ‘***’ 0.001 ‘**’ 0.01 ‘*’ 0.05 ‘.’ 0.1 ‘ ’ 1

Model quality Scores
Concordance = 0.822 (se = 0.075)
Rsquare = 0.684 (max possible= 0.989)
Likelihood ratio test = 26.47 on 11 df, p=0.005513
Wald test = 16.19 on 11 df, p=0.1344
Score (logrank) test = 22.14 on 11 df, p=0.02335

3.2.3 Age: 18M

R formula to calculate Cox model

coxph(formula = my.surv.object ~ BMI + CHr + Fe + HGB + Mg + nBcells + nLYMPH + nNEUT + pctHCT + RBC + T4, data)

Cox model

Feature	coef	exp(coef)	se(coef)	z	Pr(>|z|)	Signif.
BMI	-8.305	0.000247	3.392	-2.448	0.01436	*
CHr	0.5833	1.792	1.293	0.451	0.6519	
Fe	1.36	3.895	0.8945	1.52	0.12846	
HGB	-7.888	0.000375	2.415	-3.266	0.00109	**
Mg	0.6625	1.94	0.6339	1.045	0.29596	
nBcells	-3.038	0.04792	1.098	-2.766	0.00568	**
nLYMPH	1.837	6.281	0.9175	2.003	0.04521	*
nNEUT	2.821	16.8	1.248	2.26	0.0238	*
pctHCT	8.18	3569	3.818	2.143	0.03215	*
RBC	-2.333	0.09704	2.354	-0.991	0.32178	
T4	5.056	156.9	1.837	2.752	0.00593	**

Signif. codes: 0 ‘***’ 0.001 ‘**’ 0.01 ‘*’ 0.05 ‘.’ 0.1 ‘ ’ 1

```
Model quality Scores
Concordance          = 0.93  (se = 0.084 )
Rsquare              = 0.893   (max possible= 0.984 )
Likelihood ratio test = 42.42  on 11 df,   p=1.37e-05
Wald test            = 14.78  on 11 df,   p=0.1926
Score (logrank) test = 35.05  on 11 df,   p=0.0002432
```

3.2.4 Age: 24M

R formula to calculate Cox model

coxph(formula = my.surv.object ~ CHr + HGB + nBcells + nLYMPH + nNEUT + pctHCT + RBC, data = model.data.complete)

Cox model

Feature	coef	exp(coef)	se(coef)	z	Pr(>|z|)	Signif.
CHr	464.6	6.057E+201	8104	0.057	0.954	
HGB	-132.7	2.358E-58	3417	-0.039	0.969	
nBcells	-9.566	0.00007008	1177	-0.008	0.994	
nLYMPH	-108.9	5.247E-48	2116	-0.051	0.959	
nNEUT	-58.22	5.188E-26	958.5	-0.061	0.952	
pctHCT	-89.69	1.121E-39	4739	-0.019	0.985	
RBC	50.28	6.875E+21	5496	0.009	0.993	

Signif. codes: 0 '***' 0.001 '**' 0.01 '*' 0.05 '.' 0.1 ' ' 1

Model quality Scores
Concordance = 1 (se = 0.124)
Rsquare = 0.944 (max possible= 0.951)
Likelihood ratio test = 28.82 on 7 df, p=0.0001559
Wald test = 424.9 on 7 df, p=0
Score (logrank) test = 19.75 on 7 df, p=0.006134
